# Synthesis of a π‐Extended Phenazine Diimide Derivative and Characterization of Its Closed‐Shell Reduced States

**DOI:** 10.1002/chem.202503285

**Published:** 2026-02-15

**Authors:** Francesco Rigodanza, Beatrice Bartolomei, Ilaria Crea, Paolo Costa, Nicola Demitri, Marcella Bonchio, Maurizio Prato, Jacopo Dosso

**Affiliations:** ^1^ Department of Chemical Sciences, INSTM UdR Padova and Institute of Membrane Technology, ITM‐CNR UoS Padova University of Padova Padova Italy; ^2^ Department of Chemical and Pharmaceutical Sciences CENMAT Centre of Excellence for Nanostructured Materials INSTM UdR Trieste University of Trieste Trieste Italy; ^3^ Elettra—Sincrotrone Trieste Italy; ^4^ Basque Fdn Sci Ikerbasque Bilbao Spain; ^5^ Centre For Cooperative Research in Biomaterials (CIC BiomaGUNE) Basque Research and Technology Alliance (BRTA) Donostia San Sebastián Spain

**Keywords:** closed‐shell, dianions, Meisenheimer complex, phenazine, rylene diimide

## Abstract

In this work, a systematic synthesis of π‐extended phenazine diimide derivatives is reported along with an extensive characterization of the optoelectronic properties of these molecules. The synthetic approach, based on the functionalization of naphthalene imide derivatives, enables both the direct synthesis of phenazine diimides and the preparation of phenazine dianhydride, thus resulting in a great synthetic versatility. Also, a comprehensive computational and spectroscopic characterization of the closed‐shell reduced forms of phenazine diimides was carried out. The results indicate the differential formation of a dihydrophenazine or of an uncommon Meisenheimer complex, depending on the reduction conditions. These results highlight the great importance of structural design and reaction conditions in determining the nature of the reduced form of organic molecules, with important implications for the applications of these species as reagents and catalysts.

## Graphical abstract

In this work, the synthesis and characterization of novel π‐extended phenazine diimides is reported. The derivatives undergo differential reduction to a dihydrophenazine or Meisenheimer form depending on the reaction conditions employed. The outcome of this work highlights the paramount importance of structural design in determining the nature of the closed‐shell reduced states of rylene diimides.

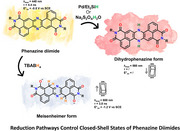

## 1 INTRODUCTION

**FIGURE 1:**
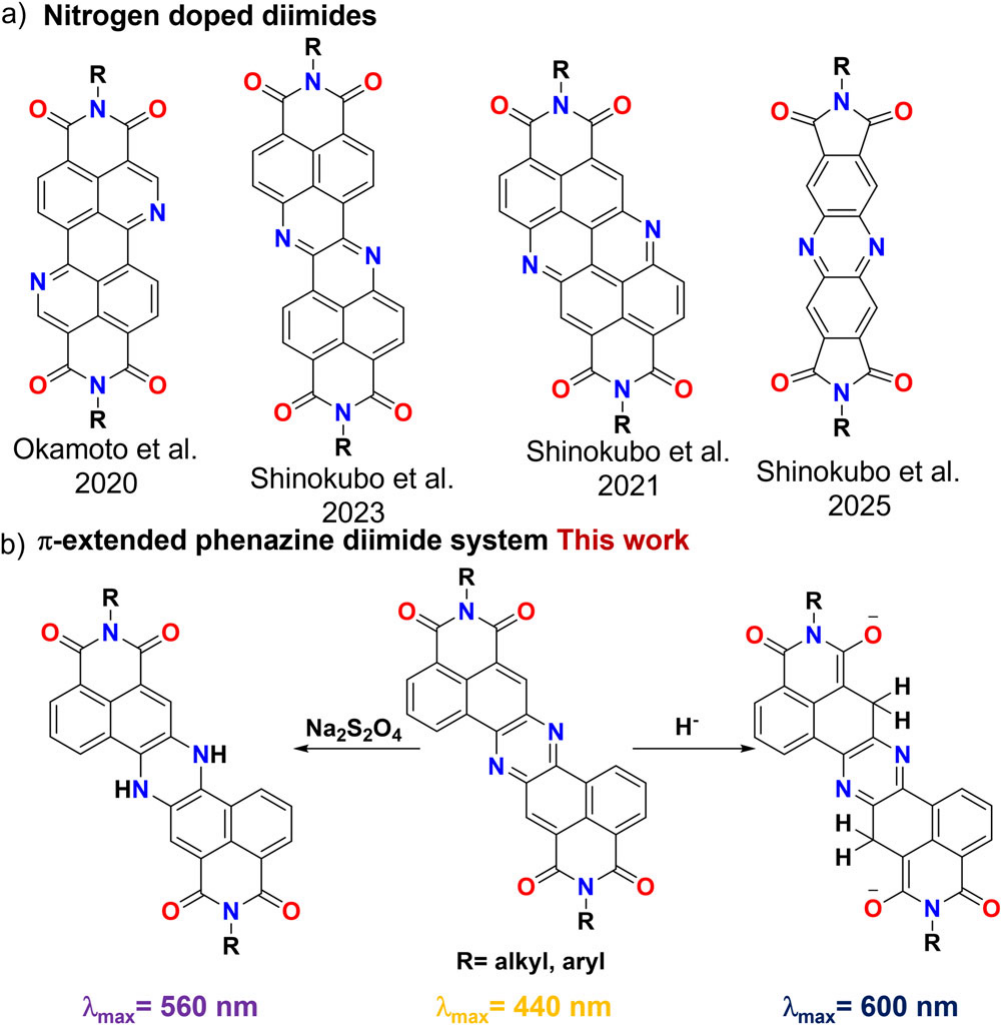
(a) Previously reported nitrogen‐doped diimide systems; (b) the differential reduction of the phenazine diimide system to dihydrophenazine (left) and Meisenheimer complex (right).

The preparation and study of rylene diimides is a thriving area in organic chemistry, mostly due to their marked electron affinities, high stability, and enhanced optoelectronic properties [1]. These characteristics are associated to potential applications in a wide range of fields, from semiconductors [2], to light harvesting [3], catalysis [4, 5, 6], and supramolecular assemblies [7]. Recently, along with the functionalization of perylene diimide (PDI) [8] and naphthalene diimide (NDI) [9], a lot of efforts have been devoted to preparing diimide derivatives presenting modified polyaromatic (PAH) backbones different from perylene and naphthalene. Particularly, diimide derivatives of pyrene [10], anthracene [11], and extended PAHs have been reported [12, 13]. Along with this approach, also the concomitant inclusion of heteroatoms such as O, N, B, and others has been exploited to induce modifications of the optoelectronic properties of diimide derivatives [14, 15, 16, 17, 18]. Among the different strategies, the inclusion of nitrogen is of great interest (Figure 1a). Indeed, when this element is introduced in the pyridinic form in the PAH backbone of rylene diimides, it results in a lowering of the LUMO energy levels, which coupled with π‐extension, can potentially result in an enhanced stabilization of the reduced forms of these molecules [19].

These aspects are particularly attractive from a theoretical and applicative point of view [16, 20, 21]. Indeed, recently, there has been significant interest in exploiting the nature and stability of the reduced forms of rylene diimides in photocatalysis, and particularly of closed‐shell dianion species, given their potential as powerful super reductants [22, 23, 24]. From this point of view, it has been demonstrated that the nature of the reduced species can differ dramatically depending on the structure of the system, going from dianionic states with aromaticity and charge redistribution as in the PDI [22], to more complex Meisenheimer systems as reported for naphthalene monoimides (NMI) [23] and electron poor benzene rings [25].

In this context, merging diimide systems with electron deficient heteroaromatic rings such as phenazine, could effectively lead to the stabilization of their reduced states, resulting in a highly promising pathway toward the development of a new class of tuneable systems with properties depending on the oxidation state and the nature of the reducing agent employed [26]. Indeed, phenazine derivatives are well known to undergo reversible reduction with common chemical agents such as Na_2_S_2_O_4_ [27] and SnCl_2_ [28] in protic conditions, resulting in the formation of dihydrophenazines, electron‐rich species that exhibit intriguing redox behavior [29, 30] and have been successfully employed in photocatalytic applications [31, 32, 33]. On the other hand, treatment of phenazine diimides with strong reducing agents in aprotic solvents might result either in the formation of Meisenheimer complexes or antiaromatic phenazine dianions, both of which represent highly reactive closed‐shell species [23, 34, 35]. From this point of view, the accessibility of these exotic states could be further improved by increasing the π‐extension of these systems, resulting in improved charge distribution and lessened Coulombic repulsion. Based on these premises, the synthesis of π‐extended phenazine diimides is reported in this paper, along with a comprehensive computational and spectroscopical characterization of their closed‐shell dihydrophenazine and Meisenheimer complex states. These results are highly important in disclosing the importance of doping patterns and reducing agents in the formation of rylene diimide reduced forms. In particular, the structural elucidation of a diimide Meisenheimer complex offers new insights into the reactivity and stabilization of closed‐shell reduced species, broadening the scope of rylene‐based materials for photocatalytic and redox‐driven applications.

## 2 Results and Discussion

**SCHEME 1:**
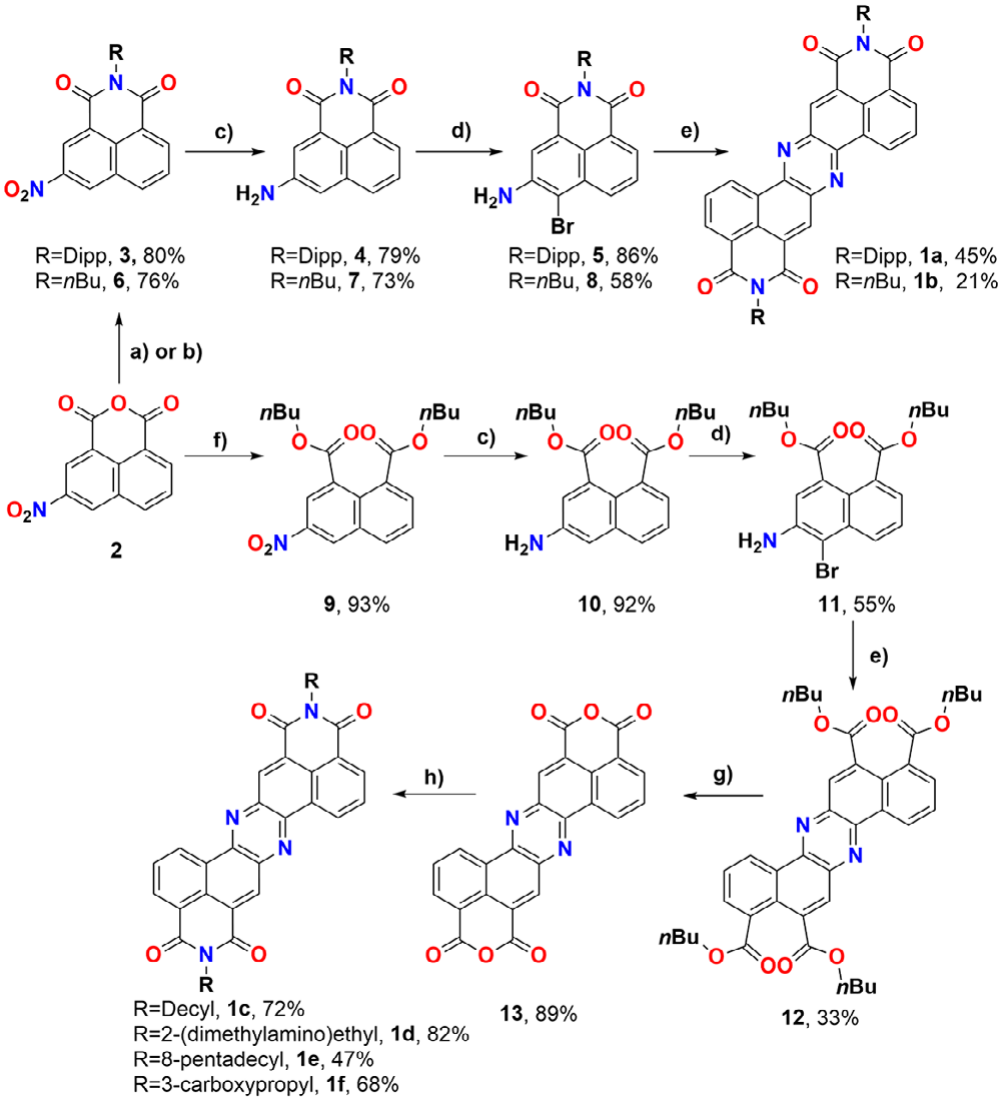
(a) 2,6‐Diisopropylaniline, AcOH, 110°C, 18 h; (b) *n*BuNH_2_, EtOH, 80°C, 18 h; (c) SnCl_2_∙2H_2_O, EtOAc/EtOH, 60°C, 18 h; (d) Br_2_, Dioxane, 80°C, 1 h; (e) Pd(OAc)_2_, SPhos, Cs_2_CO_3_, Dioxane, 110°C, 18 h; (f) *n*BuBr, *n*BuOH, DBU, DMF, 60°C, 3 h; (g) PTSA∙H_2_O, toluene, reflux, 18 h; and (h) amine, DMF, MW, 130°C, 30 min to 1 h.

**FIGURE 2:**
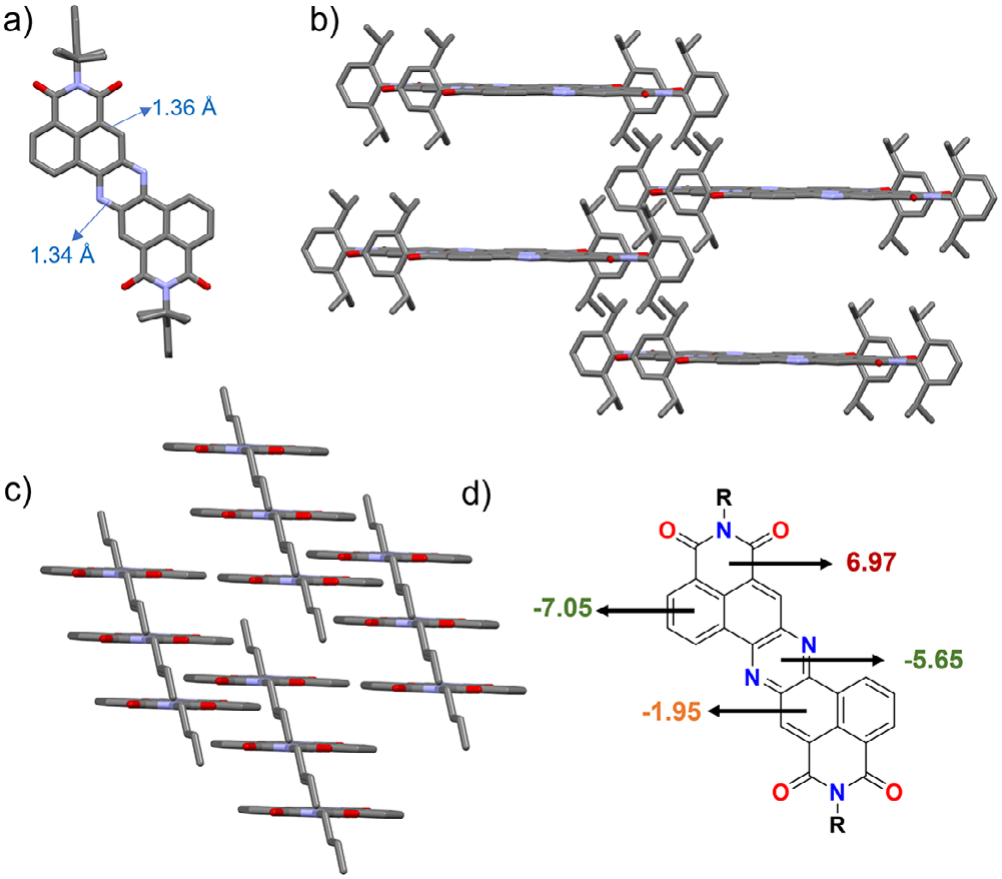
(a) Top view and (b) packing detail of **1a** space group: P‐1; (c) packing detail of **1b**, space group P‐1; crystals obtained from diffusion of MeOH in *o*DCB. Hydrogens omitted for clarity. O: red, N: blue, C: gray; (d) NICS(0) values for **1a** calculated at the (U)M062x/Def2TZVP/IEFPCM (THF) level of theory.

The first approach for the preparation of π‐extended phenazine diimide derivatives was carried out using functionalized diisopropyl phenyl (Dipp) naphthalene monoimide **5** as a building block, whose synthesis is detailed in Scheme 1 and in a previous contribution from our group [36]. The bromo amino derivative **5** was then reacted in the presence of Pd(OAc)_2_, SPhos and Cs_2_CO_3_ in dioxane at 110°C for 18 h, following the procedure reported by Winkler et al. [37]. After purification, the desired derivative **1a** was isolated as a bright yellow powder in a 45% yield, and thoroughly characterized via NMR spectroscopy, high resolution mass spectroscopy (HRMS), and single crystal X‐Ray diffraction (sc‐XRD, Figure 2a). The ^1^H‐NMR spectra highlighted the presence of a strongly deshielded set of aromatic signals (Figure S13) compared to precursor **5** [36], in line with the formation of an electron‐accepting π‐extended system. Also, ^13^C‐NMR, confirmed the presence of the imide system (signals at 164.5 and 163.9 ppm), which together with HRMS proved the formation of the desired molecule. Having confirmed the nature of **1a**, the same synthetic strategy was successfully employed to prepare derivative **1b** (Scheme 1) presenting a *n‐*Butyl (*n*Bu) chain in place of Dipp, which proved to be much less soluble than **1a**. Despite the efficient obtainment of derivatives **1a** and **1b**, when the synthetic strategy was extended to imide functional groups presenting polar moieties (1‐hydroxyethyl or 2‐(dimethylamino)ethyl) the desired products could be obtained only with very low yields. To provide a more general way to synthesize these derivatives, it was decided to attempt the synthesis of dianhydride derivative **13**. Aiming at this, **2** was converted to dibutyl ester **9** by treatment with DBU in presence of *n*BuOH and *n*BuBr, smoothly affording the desired product in an almost quantitative yield. As in the previous syntheses, **9** was reduced to **10** in a very good 92% yield, and the obtained amine derivative was in turn treated with bromine to give **11** in a 55% yield. Exploiting the same conditions used for **5**, tetrabutylester phenazine **12** was obtained in a 33% yield.

Treatment of **12** with *para*‐toluenesulfonic acid monohydrate (PTSA·H_2_O) in refluxing toluene afforded dianhydride **13** as a highly insoluble yellow powder, which could be isolated by centrifugation. Despite the derivative insolubility, treatment with amines (decylamine, *N,N*‐dimethylethylenediamine, or 8‐pentadecanamine) in DMF at 130°C in a MW reactor, resulted in the formation of derivatives **1c**, **1d**, and **1e** in good yields (72%, 82%, and 47%, respectively) and very short reaction times (30 min–1 h). Moreover, to achieve water‐soluble derivatives, dianhydride **13** was reacted in the same conditions with γ‐aminobutyric acid, resulting in the formation of derivative **1f** in a 68% yield, which proved to be soluble in aqueous 50 mM Cs_2_CO_3_.

To unveil the properties of this class of molecules, all derivatives were extensively characterized via NMR, UV–vis and HRMS spectroscopies; moreover, in the case of **1a** and **1b**, crystals for both molecules could be obtained by diffusion of MeOH in *ortho*‐dichlorobenzene (*o*DCB), providing structural confirmation for both derivatives (Figure 2a–c). In both cases, the π‐system is completely flat, and the C═N bonds are equalized with a value of 1.34 Å, which is consistent with those of a C═N aromatic double bond. Moreover, the C─C bond comprised between the imide system and the phenazine (Figure 2a) presents a slightly shorter length (1.36 Å) than that expected for an aromatic bond (1.40 Å), suggesting a tiny enhancement of double bond character. This is consistent with the presence of fully localized aromatic sextets on the pyrazine and the external rings of the naphthalene system, which present a more marked bond equalization. This fact seems to suggest that (in line with NICS calculations, Figure 2d) the pyrazine ring is conjugated and aromatic, indicating communication between the two naphthalene subunits. As expected, the presence of the Dipp substituent results in the disruption of the π‐stacking between aromatic systems, which present large spacing between molecules (Figure 2b). On the contrary, when *n*Bu substituents are in place, the crystal packing is organized in columnar stacks of molecules with an interlayer distance of 3.338 Å, which is fully consistent with the reduced solubility of linear alkyl chain substituted derivatives (Figure 2c). In both cases, the pyrazine nitrogen atoms are not involved in supramolecular interactions due to the steric hindrance provided by the neighboring phenyl rings of the naphthalene system.

We then investigated the structural and electronic properties of the phenazine diimides to uncover design principles relevant for their use in photocatalysis and redox‐active molecular systems.

**FIGURE 3:**
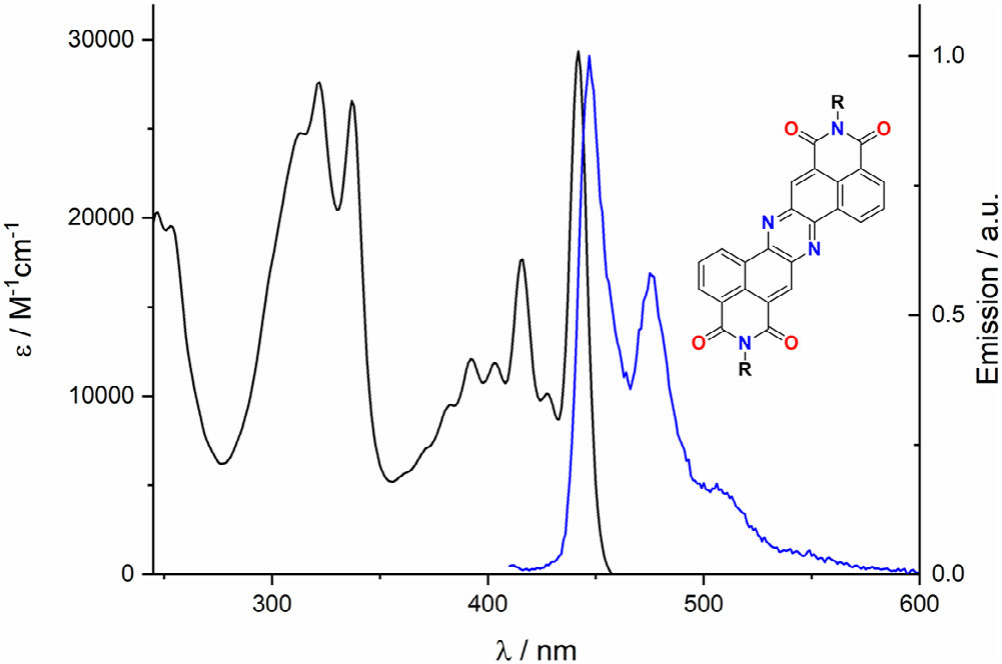
(a) Absorption (black) and fluorescence spectrum (blue) of **1a** in air equilibrated CHCl_3_ at room temperature. *λ*_ex_ = 400 nm. ***R*** = Dipp for **1a**.

Considering the UV–vis properties, all molecules present a structured absorption (*λ*_max_ = 442 nm) and emission (*λ*_em_ = 446 nm) in the visible region (Figure 3 for derivative **1a**), an *E*_00_ of 2.8 eV and a very narrow Stokes shift (ca. 0.025 eV) suggesting a highly rigid structure which is associated to a modest QY < 5%, in line with relat structures [20]. Moreover, in all derivatives, both absorption and emission are unaffected by the presence of a Dipp or alkyl chain bonded to the imide, suggesting a similar behavior to PDIs and in line with HOMO and LUMO calculations (Figure S70) in which a node is visible on the imide nitrogen.

**FIGURE 4:**
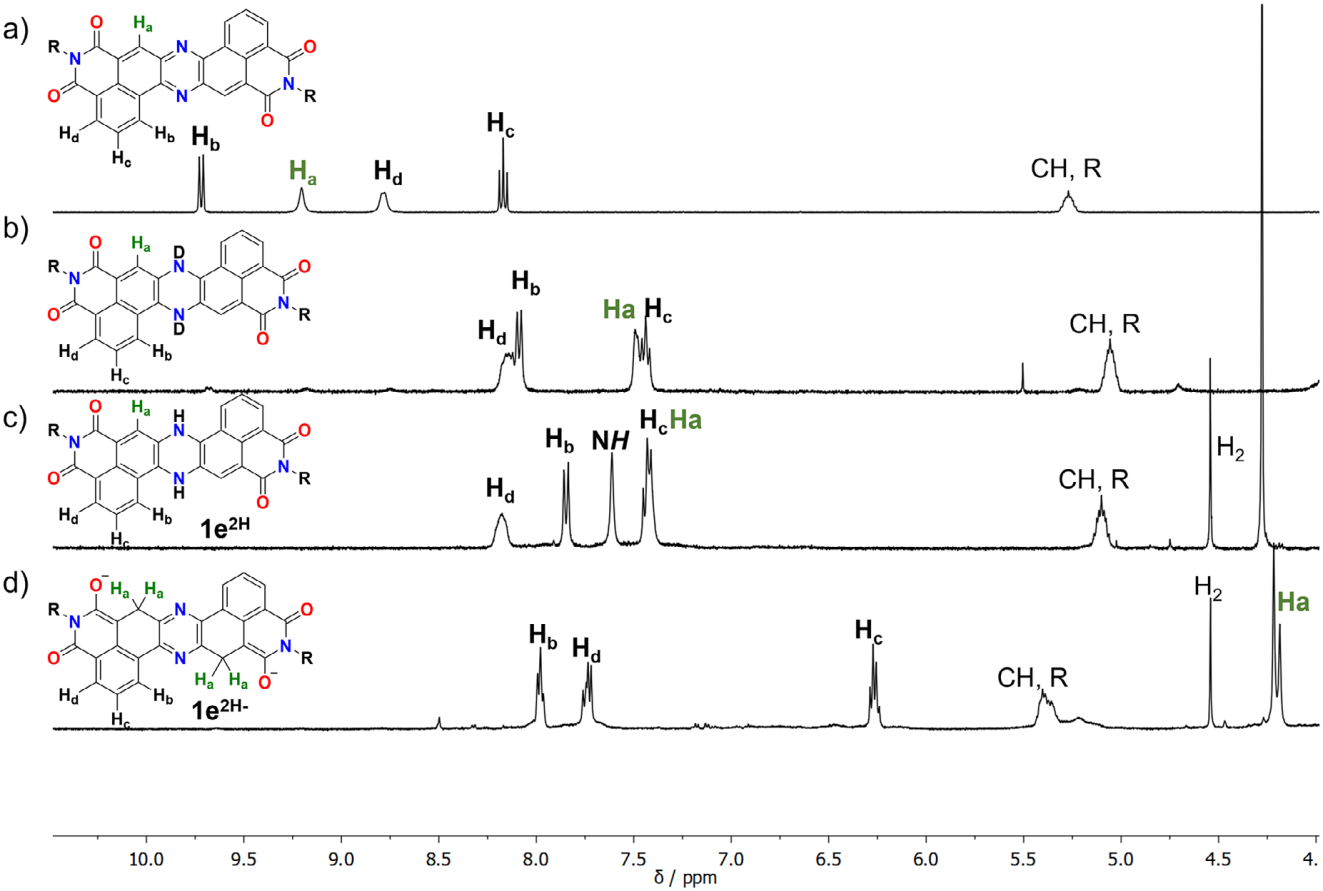
^1^H‐NMR in THF‐*d8* of (a) **1e**; (b) **1e**, Na_2_S_2_O_4_ excess and D_2_O; (c) **1e**, Et_3_SiH excess and Pd(OAc)_2_ catalytic; and (d) **1e**, TBABH_4_ excess. H_2_ is visible at 4.55 ppm [41].

At this point, derivative **1e** was studied via cyclic voltammetry (CV) measurements in THF (Figure S62). The choice of **1e** is mainly related to the superior solubility in organic solvents imparted by the branched chain. The CVs highlighted the presence of two well‐resolved reversible reduction waves with *E*_1/2_ of −0.51 and −1.04 V versus SCE, with a *E*_onset_ of −0.4 V, whereas no oxidation waves were detected in THF electrochemical window. From this set of measurements, the LUMO was estimated to be −4.15 eV [38], suggesting an electron‐accepting system as expected due to the presence of the phenazine and diimide. Given the relative accessibility of the reduced states highlighted by CV experiments, a chemical reduction of the system was then explored using NMR spectroscopy (Figure 4b). In a first attempt, **1e** was treated with a large excess of Na_2_S_2_O_4_ in a degassed 3/1 THF‐*d8*/D_2_O mixture, resulting in the appearance of a new set of up‐field shifted signals (Figure 4a,b). The observed signals were compatible with the formation of the dihydrophenazine system, which was also in agreement with the 2D‐COSY NMR spectra (Figure S40). Interestingly, the conversion proved to be reversible on multiple cycles by exposure to atmospheric oxygen and subsequent treatment with Na_2_S_2_O_4_ (See Figures S41 and S42). Despite this, the presence of D_2_O in the NMR tube prevented the observation of the diagnostic N*H* signal. To overcome this limitation, alternative reductive conditions were achieved employing catalytic Pd(OAc)_2_ in presence of an excess of Et_3_SiH [39], this time using degassed THF‐*d8* as sole solvent (Figure 4c). As a result, a very similar set of signals compared to the previous case was observed; however this time, the N*H* proton was clearly visible at 7.61 ppm, which is in line with the reduction of a less extended derivative [26]. This was further confirmed by the disappearance of the signal following addition of D_2_O (Figure S45) and by ^1^H‐^15^N HSQC measurements (Figure S46). Building on these results, we decided to use a strong hydride reducing agent such as tetrabutyl ammonium borohydride (TBABH_4_) to carry out the same reduction in pure degassed THF‐*d8* to assess whether this would result in the formation of an antiaromatic dihydrophenazine dianion, a closed shell dianion (like in the case of PDI) or in a Meisenheimer complex (as in the case of naphthalene monoimide). Upon addition of TBABH_4_ a deep blue color was observed and from the ^1^H‐NMR spectra (Figure 4d), it was possible to see that only three main aromatic signals were present, while two sets of singlets appeared at 4.20 ppm, similar to the one reported by Nocera [23]. From 2D‐HSQC and HMBC studies it emerged that this signal is due to a CH_2_ group that correlates directly with ^13^C signals at 33.75 and 34.24 ppm (the splitting of the ^13^C signals is ascribable to the hindered rotation of the solubilizing chain resulting in different rotamers as observed in **1e** and in other swallowtailed diimide derivatives [40]), and on multiple bonds correlates with aromatic signals (140.44, 145.92, and 149.32 ppm) and more importantly with signals associated with the enolate system (83.23 and 84.24 ppm). Altogether these data prompted us to postulate the Meisenheimer complex structure **1e^2H−^** reported in Figure 4d. This, in turn, led us to hypothesize that reduction in absence of hard nucleophiles favors the formation of the dihydrophenazine system, consistent with the well‐known phenazine reduction, while the use of hydride reducing agents promotes nucleophilic attack to yield the Meisenheimer complex. The formation of the latter is plausibly addressed by the stabilization arising from the delocalization of the negative charge between the imide and phenazine system, and also by the relatively limited aromaticity of the ring subjected to the nucleophilic attack (Figure 2d), which is not a full sextet in the Clar resonance structure.

**FIGURE 5:**
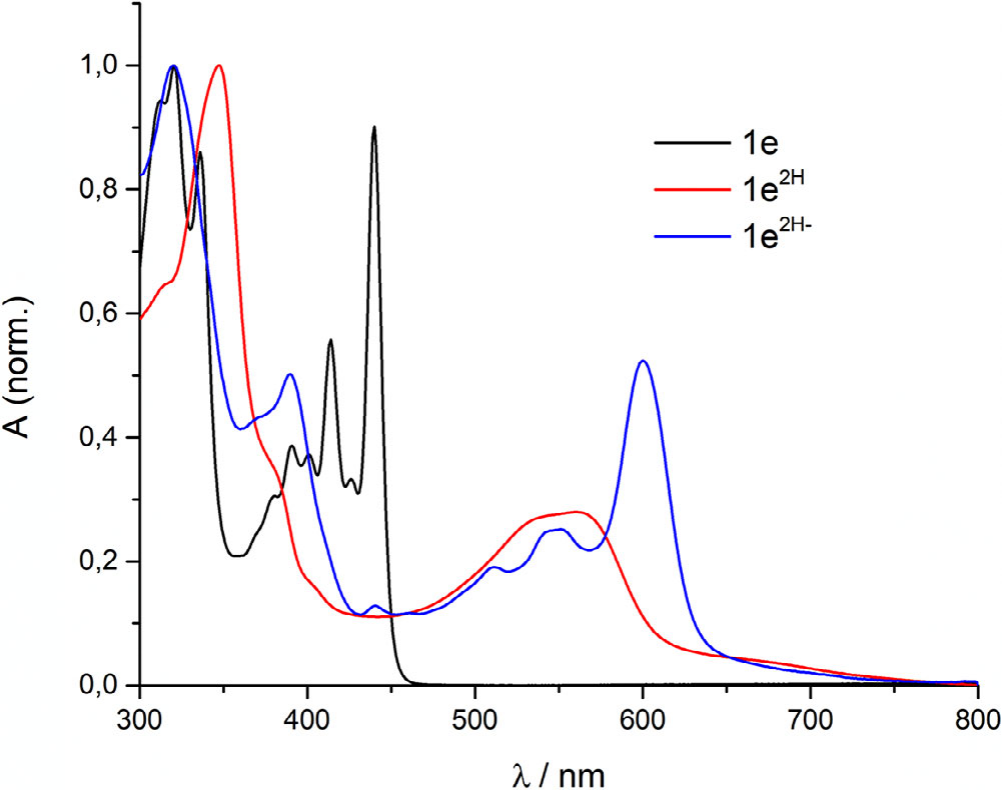
Normalized absorption in THF of **1e** (black); **1e^2H^**, Et_3_SiH excess and Pd(OAc)_2_ catalytic (red); and **1e^2H−^**, TBABH_4_ excess (blue).

To further study the nature of these reduced forms, UV–vis characterization was carried out in THF under inert atmosphere (Figure 5) using Pd(OAc)_2_‐Et_3_SiH and TBABH_4_. After chemical reduction with the mild Pd(OAc)_2_/Et_3_SiH system, the initially yellow solution turned violet and a broad, featureless absorption band centered at 560 nm appeared. TD–DFT predicts for the dihydrophenazine **1e^2H^** a lowest‐energy transition at 470 nm (*f* = 0.3), in reasonable agreement with this new band and confirming assignment to the two‐electron/proton reduced species (Figure S68). In contrast, treatment with the stronger hydride donor TBABH_4_ caused an immediate color change from yellow to deep blue and the emergence of a structured absorption in the red region with *λ*_max_ ≈ 600 nm, matching the TD–DFT prediction for the Meisenheimer anion **1e^2H−^** (calculated 537 nm, *f* = 0.55; Figure S69). The residual blue‐shift of the computed values relative to experiment is typical for TD–DFT in polar media and likely reflects the simplified continuum solvation model and the neglect of explicit ion pairing and vibronic coupling [42].

Moreover, the Meisenheimer complex **1e^2H−^** displays a distinct luminescence with a maximum emission at 636 nm, a fluorescence lifetime of 3.8 ns, and an estimated *E*_00_ of 2.0 eV (Figures S58 and S59). The narrow Stokes shift and nanosecond‐scale lifetime indicate emission from a singlet‐excited state. CV of **1e^2H−^** reveals a reversible reduction at −1.18 V and an anodic oxidation at +0.90 V versus SCE, defining a broad > 2 V window (Figures S64 and S65). For comparison, the hydride Meisenheimer [NMI(H)]^−^ displays an oxidation near –0.3 V versus SCE, that is, ∼1.2 V more cathodic than **1e^2H−^** [23]. This substantial anodic shift highlights the decreased reducing nature of our π‐extended phenazine–diimide framework, possibly implying higher persistence under turnover and greater compatibility with oxidative or biased conditions. While the less exergonic excited‐state donation suggests a milder—hence potentially more selective—photoreductant than other Meisenheimer structures used for photocatalysis [43], the red‐shifted optical addressability (visible‐red excitation) and broader electrochemical stability make **1e^2H−^** a potential platform for photoinduced electron transfer and photocatalytic applications.

## 3 Conclusions

In this work, we established a modular synthetic access to π‐extended phenazine diimide derivatives and elucidated how their molecular structure controls the nature and properties of their closed‐shell reduced states. Single‐crystal X‐ray diffraction reveals a rigid and planar π‐framework, while NICS analysis evidences a nonuniform aromaticity distribution across the phenazine–naphthalene scaffold.

The neutral phenazine diimides display structured absorption and emission in the visible region (*λ*_max_ ≈ 442 nm, *E*_00_ ≈ 2.8 eV) with very small Stokes shifts, reflecting a rigid conjugated backbone. Electrochemical measurements reveal two reversible reductions (*E*_1/2_ = −0.51 and −1.04 V versus SCE), indicating ready access to reduced states. A central outcome of this study is that reduction conditions selectively define the electronic structure of the closed‐shell products. Mild proton‐coupled reduction affords a neutral dihydrophenazine, identified by a broad absorption band centered at ≈ 560 nm, whereas strong hydride donors promote nucleophilic attack to yield a rare rylene–diimide Meisenheimer complex. The latter exhibits red‐shifted absorption (*λ*_max_ ≈ 600 nm), emission at 636 nm, a singlet excited‐state lifetime of 3.8 ns, and a wide electrochemical window exceeding 2 V (*E*_red_ ≈ −1.18 V, *E*_ox_ ≈ +0.90 V versus SCE).

The formation of the Meisenheimer complex is rationalized by the limited aromatic stabilization of the attacked ring establishing a direct link between local aromaticity and reduction pathway. These findings define clear structure–reactivity relationships in phenazine diimides and provide quantitative benchmarks for the design of π‐extended rylene systems with controllable closed‐shell redox states.

## Conflicts of Interest

The authors declare no conflict of interest.

## Supporting information




**Supporting File 1**: Supporting Information for this article is available free of charge at https://onlinelibrary.wiley.com/. Deposition Numbers 2280881 (for **1a** at 100K), 2280882 (for **1a** at 298K), 2280883 (for **1b** at 100K) contain the supplementary crystallographic data for this paper. These data are provided free of charge by the joint Cambridge Crystallographic Data Centre and Fachinformationszentrum Karlsruhe http://www.ccdc.cam.ac.uk/structures.

**Supporting File 2**: 1aLT.cif.

**Supporting File 3**: 1aRT.cif.

**Supporting File 4**: 1b.cif.
