# Supplementary material for: Synthesis of a π‐Extended Phenazine Diimide Derivative and Characterization of Its Closed‐Shell Reduced States
Source: Chemistry. 2026 Feb 15;32(17):e03285. doi: 10.1002/chem.202503285 (PMC13150043; doi:10.1002/chem.202503285)
Supplement: Supplementary file 1 — Supporting File 1: Supporting Information for this article is available free of charge at https://onlinelibrary.wiley.com/. Deposition Numbers 2280881 (for 1a at 100K), 2280882 (for 1a at 298K), 2280883 (for 1b at 100K) contain the supplementary crystallographic data for this paper. These data are provided free of charge by the joint Cambridge Crystallographic Data Centre and Fachinformationszentrum Karlsruhe http://www.ccdc.cam.ac.uk/structures. [file CHEM-32-e03285-s002.docx]

Synthesis of a π-Extended Phenazine Diimide Derivatives and Characterization of its Closed-shell Reduced States

**Supporting Information**

Francesco Rigodanza*^e^, Beatrice Bartolomei^a^, Ilaria Crea^e^, Paolo Costa^e^, Nicola Demitri^d^, Marcella Bonchio^*e,f^, Maurizio Prato^a,b,c^, Jacopo Dosso*^,a^

[a] Department of Chemical and Pharmaceutical Sciences, CENMAT, Centre of Excellence for Nanostructured Materials, INSTM UdR Trieste, University of Trieste, via Licio Giorgieri 1, 34127 Trieste, Italy.

[b] Centre for Cooperative Research in Biomaterials (CIC BiomaGUNE), Basque Research and Technology Alliance (BRTA), Paseo de Miramón 194, 20014, Donostia San Sebastián, Spain

[c] Basque Fdn Sci, Ikerbasque, 48013 Bilbao, Spain.

[d] Elettra—Sincrotrone, Trieste S.S. 14 Km 163.5, Area Science Park, 34149 Basovizza, Trieste (Italy)

[e] Department of Chemical Sciences, University of Padova,Via Marzolo 1,35131 Padova, Italy;

[f] Interuniversity Consortium on Materials Science and Technology, INSTM UdR Padova and Institute of Membrane Technology, ITM-CNR UoS Padova, Via Marzolo 1 Padova, 35131 Padova, Italy;

Summary

[**1.** **General Remarks** 2](#_Toc220420080)

[**1.1** **Instrumentation** 2](#_Toc220420081)

[**1.2** **Materials and methods** 3](#_Toc220420082)

[**2** **Synthetic procedures and spectral data** 4](#_Toc220420083)

[**2.0** **Synthesis of 2** 4](#_Toc220420084)

[**2.1** **Synthesis of 6** 4](#_Toc220420085)

[**2.2** **Synthesis of 7** 5](#_Toc220420086)

[**2.3** **Synthesis of 8** 5](#_Toc220420087)

[**2.4** **Synthesis of 1b** 6](#_Toc220420088)

[**2.5** **Synthesis of 1a** 7](#_Toc220420089)

[**2.6** **Synthesis of 9** 8](#_Toc220420090)

[**2.7** **Synthesis of 10** 8](#_Toc220420091)

[**2.8** **Synthesis of 11** 9](#_Toc220420092)

[**2.9** **Synthesis of 12** 10](#_Toc220420093)

[**2.10** **Synthesis of 13** 10](#_Toc220420094)

[**2.11** **Synthesis of 1c** 11](#_Toc220420095)

[**2.12** **Synthesis of 1d** 12](#_Toc220420096)

[**2.13** **Synthesis of 1e** 13](#_Toc220420097)

[**2.14** **Synthesis of 1f** 14](#_Toc220420098)

[**3** **NMR and HRMS spectroscopic characterization** 15](#_Toc220420099)

[**3.1** **Characterization of 6** 15](#_Toc220420100)

[**3.2** **Characterization of 7** 17](#_Toc220420101)

[**3.3** **Characterization of 8** 18](#_Toc220420102)

[**3.4** **Characterization of 1b** 20](#_Toc220420103)

[**3.5** **Characterization of 1a** 22](#_Toc220420104)

[**3.6** **Characterization of 9** 24](#_Toc220420105)

[**3.7** **Characterization of 10** 25](#_Toc220420106)

[**3.8** **Characterization of 11** 27](#_Toc220420107)

[**3.9** **Characterization of 12** 28](#_Toc220420108)

[**3.10** **Characterization of 1c** 30](#_Toc220420109)

[**3.11** **Characterization of 1d** 31](#_Toc220420110)

[**3.12** **Characterization of 1e** 32](#_Toc220420111)

[**3.13** **Characterization of 1f** 34](#_Toc220420112)

[**4** **Reduced states NMR characterization** 35](#_Toc220420113)

[**5** **Photophysical characterization** 43](#_Toc220420114)

[**6** **Electrochemical characterizations** 46](#_Toc220420115)

[**7** **Crystallographic characterizations** 48](#_Toc220420116)

[**8** **DFT calculations** 52](#_Toc220420117)

[**9** **References** 58](#_Toc220420118)

# **1. General Remarks**

## **1.1 Instrumentation**

***Thin layer chromatography*** (TLC) was conducted on Sigma Aldrich pre-coated aluminium sheets (0.25 mm layer thickness, 60 Å porosity and fluorescent indicator GF254) and were visualized using 254 or 365 nm light. Flash column chromatography was carried out using Merck Gerduran silica gel 60 (particle size 40 63 μm). ***Melting points*** (M.P.) were measured on a Gallenkamp apparatus. All of melting points have been measured in open capillary tubes and have not been corrected. ***Nuclear magnetic resonance*** (NMR) ^1^H, and ^13^C spectra were obtained on Varian Inova spectrometer (500 MHz ^1^H and 126 MHz ^13^C), Varian 400 MHz NMR spectrometer (400 MHz ^1^H and 101 MHz ^13^C) or Bruker Avance III™ HD 600 MHz (^1^H-^15^N HSQC). Chemical shifts were reported in ppm according to tetramethylsilane using the solvent residual signal as an internal reference (CDCl_3_: δ_H_ = 7.26 ppm, δ_C_ = 77.16 ppm, CD_2_Cl_2_: δ_H_ = 5.32 ppm, δ_C_ = 53.84 ppm, MeOD: δ_H_ = 3.31 ppm, δ_C_ = 49.00 ppm, CD_3_CN: δ_H_ = 1.94 ppm, δ_C_ = 1.32, 118.26 ppm, DMSO-*d6*: δ_H_ = 2.50 ppm, δ_C_ = 39.51 ppm, THF-*d8*: δ_H_ = 3.58 and 1.72 ppm, δ_C_ = 67.21 and 25.31 ppm, TFA-*d1*: δ_H_ = 11.50 ppm, δ_C_ = 164.20 and 116.60 ppm,). Coupling constants (*J*) were given in Hz and were averaged. Resonance multiplicity was described as s (singlet), d (doublet), t (triplet), m (multiplet), br (broad signal), dd (doublet of doublets), dt (doublet of triplets). Carbon spectra were acquired with a complete decoupling for the proton, unless specified. All spectra were recorded at 25 °C unless specified. ***Infrared spectra*** (IR) were recorded on a Shimadzu IR Affinity 1S FTIR spectrometer in ATR mode with a diamond mono-crystal. Selected absorption bands are reported in wavenumber (cm^-1^). ***ESI-High resolution mass spectrometry*** (ESI-HRMS). ESI-HRMS was performed at University of Trieste Chemistry department, High resolution mass spectra (HRMS) were obtained on Bruker micrOTOF-Q (ESI-TOF). ***MALDI-TOF mass analysis*** were performed at CIC-Biomagune (Donostia/San Sebastian, Spain) on a UltrafleXtreme III MALDI-TOF-MS (Bruker, Bremen, Germany). Sample plate: Bruker MALDI Plate MTP polished steel 384. Laser: Solid stated ND:YAG at 355 nm – SMART BEAM II – Freq 1kHz. The sample was prepared using the dried-droplet method. Briefly, 0.5 µL of the sample was deposited onto the MALDI plate, followed by the addition of 0.5 µL of matrix solution (10 mg/mL DCTB in THF). The spot was allowed to dry completely prior to analysis. ***Photophysical analysis***: Absorption spectra of compounds were recorded on air equilibrated solutions at room temperature with an Agilent Cary 5000 UV-Vis-NIR double beam spectrophotometer, using quartz cells with path length of 1.0 cm. Emission measurements were performed on an Edinburgh instruments FS5 spectrofluorometer using a 150 W CW Ozone-free xenon arc lamp as source and a Photomultiplier R928P (spectral coverage 200 nm – 900 nm, cooled and stabilised) as detector. Quantum yields were performed using the integrating sphere setup SC-30 on a sample solution in a quartz cuvette and using the same solvent in another cuvette as reference. Fluorescence decay dynamics studies have been performed using 635 nm laser pulses on a FLS1000 by Edinburgh Instruments equipped with a PMT-980 detector. 10 mm path length Hellma Analytics sealable quartz cuvettes have been used. ***Cyclic Voltammetry (CV****):* the electrochemical characterizations were carried out in THF/0.1 M tetrabutylammonium hexafluorophosphate (TBAPF_6_), at room temperature, on an Autolab 302 N electrochemical workstation (Metrohm, The Netherlands) in a glass cell from CH Instruments (10 mL, CHI220). A typical three-electrode cell was employed, which was composed of glassy carbon (GC) working electrode (3 mm diameter), a platinum wire as counter electrode and a saturated calomel electrode (SCE) as reference electrode (RE). Oxygen was removed by purging the THF solution with Argon. The GC electrode was polished twice before use with 0.05 and 0.1 colloidal silica polishing suspension; Pt counter electrode was polished on a flame to remove organic materials. ***X-Ray Data***: CCDC **2280881**, **2280882** and **2280883** contain the supplementary crystallographic data for **1a** at 100K, **1a** at 298K and **1b** at 100K, respectively. Related files can be obtained free of charge from The Cambridge Crystallographic Data Centre via <https://www.ccdc.cam.ac.uk/structures>.

## **1.2 Materials and methods**

Chemicals were purchased from Sigma Aldrich, TCI, Alfa Aesar and Fluorochem and were used as received unless otherwise stated. Solvents were purchased from Sigma Aldrich and Alfa Aesar, while deuterated solvents from Eurisotop and Sigma Aldrich. Anhydrous conditions were achieved by repeated cycles of flaming with a heat gun under vacuum and purging with Argon (Ar). The inert atmosphere was maintained using Argon-filled balloons equipped with a syringe and needle that was used to penetrate the silicon stoppers used to close the flask’s necks. Additions of liquid reagents were performed using plastic syringes. Degassing of solutions was performed by bubbling argon in the reaction under sonication for at least 10 minutes. Dry solvents were obtained commercially. MilliQ water was obtained from a Millipore Milli-Q Plus 185 apparatus and presented a resistivity of 18.2 MΩcm. MilliQ water was always used unless otherwise specified. Derivatives **2**-**5** were synthesised according to a published literature procedure.^[1]^

# **2 Synthetic procedures and spectral data**

## **2.0 Synthesis of 2**

In a round bottom flask, naphthalene-1,3-monoanydride (3.0 g, 15.1 mmol) was suspended in H_2_SO_4_ (30 mL) with vigorous stirring. NaNO_3_ (1.3 g, 15.3 mmol) was then added, and the reaction was heated to 100°C and stirred for 3h. After cooling to rt., the reaction was dropped in a mixture of ice and water followed by filtration on a glass frit filter. The resulting beige solid was washed extensively with water (200 mL) followed by ACN (30 mL) and Et_2_O (30 mL). The resulting solid (3.42 g) was then suspended in 150 mL of ACN and crystallized. Filtration afforded **2** as a beige crystalline material (1.9 g, 52%).

^1^H NMR (400 MHz, DMSO-*d6*) δ: 9.54 (d, *J* = 2.0 Hz, 1 H), 8.94 (d, *J* = 2.1 Hz, 1 H), 8.84 (d, *J* = 8.3 Hz, 1 H), 8.71 (d, *J* = 7.3 Hz, 1 H), 8.09 (t, *J* = 7.8 Hz, 1 H). ^13^C NMR (101 MHz, DMSO-*d6*) δ: 159.8, 159.6, 145.8, 137.2, 135.4, 131.7, 130.9, 130.6, 129.5, 124.2, 121.4, 119.9. Corresponding to literature reports.^[2]^

## **2.1 Synthesis of 6**

In a round bottom flask, 2-nitronaphthalene-1,3-monoanydride **2** (3.5 g, 14.4 mmol) was added and suspended in EtOH (80 mL). *n*BuNH_2_ (1.42 mL, 14.4 mmol) was then added, and the resulting suspension heated at 80°C for 18 h. The brown solution was then allowed to cool to r.t. and filtered on a glass frit filter followed by washing with EtOH to give **6** as a beige solid (3.26 g, 76%).

^1^H NMR (400 MHz, CDCl_3_) δ: 9.32 (d, *J* = 2.2 Hz, 1 H), 9.13 (d, *J* = 2.2 Hz, 1 H), 8.78 (dd, *J* = 7.3, 1.1 Hz, 1 H), 8.42 (d, *J* = 8.3 Hz, 1 H), 7.96-7.92 (m, 1 H), 4.23-4.19 (m, 2 H), 1.77-1.69 (m, 2 H), 1.46 (sext, *J* = 7.5 Hz, 2 H), 0.99 (t, *J* = 7.4 Hz, 3 H). ^13^C NMR (101 MHz, CDCl_3_) δ: 163.2, 162.6, 146.5, 135.6, 134.5, 131.1, 130.3, 129.2, 129.0, 124.9, 124.3, 123.4, 40.8, 30.3, 20.5, 13.9. IR (ATR) ν (cm^-1^): 3088, 2967, 2936, 2876, 1705, 1661, 1597, 1547, 1508, 1462, 1454, 1435, 1418, 1344, 1325, 1267, 1204, 1167, 1140, 1119, 1088, 1069, 1044, 1026, 999, 958, 916, 802. ESI-LRMS: [M+Na]^+^ calc. for [C_16_H_14_N_2_O_4_Na]^+^: 321.0846; found 321.0999. Corresponding to literature reports.^[3]^

## **2.2 Synthesis of 7**

In a round bottom flask, **6** (1.0 g, 3.35 mmol) was added and dissolved in a 3/1 mixture of EtOAc/EtOH (90 and 30 mL respectively). The reaction was then degassed by argon bubbling and sonication for 5 min. and SnCl_2_∙2H_2_O (3.18 g, 16.75 mmol) was then added, followed by further 5 min. degassing. The reaction was then heated at 60°C for 18 h under Ar atmosphere. The bright yellow solution was then concentred under reduced pressure and a sat. solution of K_2_CO_3_ added. The resulting suspension was extracted with EtOAc (3 × 100 mL) and the organic layer washed with H_2_O (2 × 100 mL) and brine (100 mL). The EtOAc solution was the dried over Na_2_SO_4_, filtered and evaporated under reduced pressure to afford a yellow solid which was further purified using a silica gel plug (eluent: CH_2_Cl_2_ to CH_2_Cl_2_/EtOAc 95/5) to give **7** as a bright yellow solid (652 mg, 73%).

^1^H NMR (400 MHz, CDCl_3_) δ: 8.30 (dd, *J* = 7.3, 1.1, Hz, 1 H), 8.02 (d, *J* = 2.4 Hz, 1 H), 7.91 (dd, *J* = 8.3, 0.7, Hz, 1 H), 7.59 (dd, *J* = 8.2, 7.3, Hz, 1 H), 7.29 (d, *J* = 2.4 Hz, 1 H), 4.16 (m, 2 H), 3.95 (bs, 2H), 1.74-1.66 (m, 2 H), 1.44 (sext, *J* = 7.5 Hz, 2 H), 0.97 (t, *J* = 7.4 Hz, 3 H). ^13^C NMR (101 MHz, CD_2_Cl_2_) δ: 164.8, 164.6, 146.2, 134.0, 132.0, 127.6, 127.5, 124.3, 123.2, 122.9, 122.3, 114.1, 40.6, 30.8, 21.0, 14.3. IR (ATR) ν (cm^-1^): 3485, 3445, 3412, 3356, 2957, 2928, 2868, 1690, 1651, 1614, 1597, 1580, 1516, 1464, 1447, 1435, 1387, 1344, 1300, 1273, 1258, 1234, 1217, 1186, 1138, 1026, 974, 947, 920, 903, 880, 858, 820. ESI-LRMS: [M+H]^+^ calc. for [C_16_H_17_N_2_O_2_]^+^: 269.1285; found 269.1506. Corresponding to literature reports.^[4]^

## **2.3 Synthesis of 8**

In a round bottom flask, **7** (500 mg, 1.86 mmol) was added and dispersed in dioxane (40 mL). The resulting yellow suspension was heated to 80°C until complete dissolution of **7**. Br_2_ (116 μL, 2.26 mmol) was then added dropwise at the same temperature resulting in the formation of a faint yellow precipitate. After 2 h the reaction was concentred under vacuum and then diluted with CH_2_Cl_2_ (50 mL). The resulting suspension was then washed with an aq. solution of K_2_CO_3_ (2 g in 100 mL) followed by a sodium thiosulfate one (1 g in 100 mL), H_2_O (50 mL) and brine (50 mL). The organic layers were then dried over Na_2_SO_4_, filtered and evaporated. The resulting solid was purified on a silica gel plug (CH_2_Cl_2_) to give **8** as a dark yellow powder (373 mg, 58%).

^1^H NMR (400 MHz, CDCl_3_) δ: 8.37 (dd, *J* = 7.3, 1.1 Hz, 1 H), 8.33 (dd, *J* = 8.5, 1.1 Hz, 1 H), 8.08 (s, 1 H), 7.72 (dd, *J* = 8.5, 7.3 Hz 1 H), 4.68 (bs, 2 H), 4.17-4.13 (m, 2 H), 1.74-1.66 (m, 2 H), 1.44 (sext, *J* = 7.4 Hz, 2 H), 0.97 (t, *J* = 7.4 Hz, 3 H). ^13^C NMR (101 MHz, DMSO-*d6*) δ: 163.3, 163.0, 145.4, 131.6, 129.8, 128.4, 125.8, 122.3, 122.0, 121.8, 121.2, 106.6, 39.4, 29.6, 19.8, 13.7. IR (ATR) ν (cm^-1^): 3462, 3356, 3061, 2955, 2932, 2872, 1692, 1649, 1632, 1614, 1599, 1566, 1557, 1537, 1510, 1503, 1485, 1470, 1452, 1418, 1379, 1350, 1337, 1287, 1260, 1231, 1206, 1196, 1175, 1150, 1115, 1078, 1057, 1042, 1003, 937, 924, 899, 860, 826. ESI-LRMS: [M+H]^+^ calc. for [C_16_H_16_BrN_2_O_2_]^+^: 347.0390; found 347.0475. Corresponding to literature reports.^[5]^

## **2.4 Synthesis of 1b**

In a Schlenk tube **8** (100 mg, 0.29 mmol) was added along with Pd(OAc)_2_ (3.2 mg, 0.014 mmol), SPhos (12 mg, 0.03 mmol) and Cs_2_CO_3_ (189 mg, 0.58 mmol). The solids were then placed under vacuum and the Schlenk tube filled with Ar. Anhydrous dioxane (10 mL) was then added and the resulting suspension degassed with Ar bubbling and sonication for 10 min. The reaction mixture was then placed in a preheated silicon oil bath at 110°C and stirred for 18 h. After cooling down, the solution was diluted with H_2_O (30 mL) and extracted with CH_2_Cl_2_ (3 × 50 mL). The organic layers were then washed with H_2_O (50 mL), brine (50 mL), dried over Na_2_SO_4_, filtered and evaporated under reduced pressure. The black residue was then purified using a silica plug (CH_2_Cl_2_ 100% to EtOAc 100%) followed by reprecipitation/centrifugation (6000 rpm 10 min.) from MeOH (3×) and CH_2_Cl_2_/MeOH (2×) to give **1b** as a yellow powder (16 mg, 21%).

M.P.:>300 °C. ^1^H NMR (400 MHz, CDCl_3_) δ: 9.72 (d, *J* = 8.0 Hz, 2 H), 9.29 (s, 2 H), 8.83 (d, *J* = 7.0 Hz, 2 H), 8.13 (t, *J* = 7.8 Hz, 2 H), 4.31-4.21 (m, 4 H), 1.84-1.77 (m, 4 H), 1.57-1.45 (m, 4 H), 1.03 (t, *J* = 7.3, 6 H). ^13^C NMR (126 MHz, CDCl_3_) δ: 163.8, 163.2, 143.7, 142.3, 134.0, 132.4, 131.0, 129.9, 129.3, 128.2, 127.0, 123.4, 40.9, 30.4, 20.6, 14.0. The spectrum presents a low signal to noise ratio due to poor solubility in common organic solvents. IR (ATR) ν (cm^-1^): 2957, 2930, 2870, 1703, 1659, 1622, 1601, 1505, 1468, 1441, 1360, 1339, 1290, 1273, 1231, 1217, 1194, 1171, 1155, 1132, 1113, 1092, 1065, 1044, 1011, 936, 918, 885, 855, 837. ESI-HRMS: [M+H]^+^ calc. for [C_32_H_27_N_4_O_4_]^+^ : 531.2027; found 531.2023.

## **2.5 Synthesis of 1a**

In a Schlenk tube **5** (1.0 g, 2.22 mmol) was added along with Pd(OAc)_2_ (25 mg, 0.11 mmol), SPhos (91 mg, 0.22 mmol) and Cs_2_CO_3_ (1.45 g, 4.44 mmol). The solids were then placed under vacuum and the Schlenk tube filled with Ar. Anhydrous dioxane (30 mL) was then added and the resulting suspension degassed with Ar bubbling and sonication for 10 min. The reaction mixture was then placed in a preheated silicon oil bath at 110°C and stirred for 18 h. After cooling down, the solution was diluted with H_2_O (80 mL) and extracted with CH_2_Cl_2_ (3 × 100 mL). The organic layers were then washed with H_2_O (100 mL), brine (100 mL), dried over Na_2_SO_4_, filtered and evaporated under reduced pressure. The black residue was then purified using a silica plug (CH_2_Cl_2_) followed by reprecipitation/filtration from MeOH and washing with Et_2_O to give **1a** as a yellow powder (368 mg, 45%).

M.P.:>300 °C. ^1^H NMR (400 MHz, CHCl_3_) δ: 9.85 (dd, *J* = 8.1, 1.2 Hz, 2 H), 9.48 (s, 2 H), 8.92 (dd, *J* = 7.5, 1.2 Hz, 2 H), 8.21 (t, *J* = 7.8 Hz, 2 H), 7.53 (t, *J* = 7.8 Hz, 2 H), 7.39 (d, *J* = 7.8 Hz, 4 H), 2.81 (sept, *J* = 6.8 Hz, 4 H), 1.21 (dd, *J* = 6.8, 4.2 Hz, 24 H). ^13^C NMR (101 MHz, CD_2_Cl_2_) δ 164.5, 163.9, 146.6, 144.5, 142.9, 135.0, 133.2, 131.7, 131.5, 130.7, 130.2, 130.0, 129.3, 127.5, 124.7, 123.8, 29.8, 24.32, 24.28. IR (ATR) ν (cm^-1^): 3073, 2965, 2930, 2870, 1713, 1672, 1624, 1601, 1504, 1468, 1439, 1383, 1362, 1341, 1285, 1258, 1242, 1217, 1198, 1150, 1138, 1111, 1076, 1057, 1018, 937, 920, 912, 820, 804. ESI-HRMS: [M+H]^+^ calc. for [C_48_H_43_N_4_O_4_]^+^ : 739.3279; found 739.3288. λ_abs(max)_= 442 nm, λ_em(max)_= 446 nm (exc = 400 nm) in CHCl_3_.

## **2.6 Synthesis of 9**

In a round bottom flask, 2-nitronaphthalene-1,3-monoanydride **2** (1.0 g, 4.1 mmol) was suspended in DMF (20 mL) and then *n*-BuOH (3.0 mL, 32.9 mmol) added followed by DBU (2.45 mL, 16.4 mmol). The reaction was then heated at 60°C for 30 min. during which the solution turns red. *n*-BuBr (3.55 mL, 32.9 mmol) was then added dropwise and the reaction further stirred at 60 °C for 3h. The reaction was diluted with CH_2_Cl_2_ (50 mL), washed with water (3 × 50 mL) and brine (1 × 100 mL). The organic layers were then dried over Na_2_SO_4_, filtered and evaporated. The resulting dark oil was purified on a short silica plug (PE/CH_2_Cl_2_ 1/1) to give **9** (1.42 g, 93%) as a waxy solid.

^1^H NMR (400 MHz, CDCl_3_) δ: 8.92 (d, *J* = 2.4 Hz, 1 H), 8.71 (d, *J* = 2.4 Hz, 1 H), 8.20-8.18 (m, 2 H), 7.72 (t, *J* = 7.7 Hz, 1 H), 4.36 (t, *J* = 6.1, Hz, 2 H), 4.33 (t, *J* = 6.0, Hz, 2 H), 1.83-1.73 (m, 4 H), 1.53-1.44 (m, 4 H), 0.99 (t, *J* = 7.3 Hz, 3 H), 0.98 (t, *J* = 7.4 Hz, 3 H). ^13^C NMR (101 MHz, CDCl_3_) δ: 168.1, 167.3, 144.6, 134.0, 133.6, 133.4, 132.7, 131.1, 130.2, 127.8, 127.6, 123.0, 66.2, 65.8, 30.71, 30.69, 19.36, 19.35, 13.9. IR (ATR) ν (cm^-1^): 3107, 3078, 2957, 2934, 2895, 2872, 1732, 1721, 1603, 1537, 1504, 1476, 1464, 1456, 1387, 1373, 1358, 1337, 1273, 1204, 1182, 1144, 1107, 1090, 1061, 1024, 999, 978, 964, 945, 935, 907, 893, 847, 822. ESI-HRMS: [M+Na]^+^ calc. for [C_20_H_23_NO_6_Na]^+^ : 396.1418; found 396.1448.

## **2.7 Synthesis of 10**

In a round bottom flask, **9** (1.4 g, 3.8 mmol) was added and dissolved in a mixture of EtOAc/EtOH (100 and 40 mL respectively). The reaction was then degassed by argon bubbling and sonication for 5 min. and SnCl_2_∙2H_2_O (3.6 g, 19.0 mmol) was then added, followed by further 5 min. degassing. The reaction was then heated at 60°C for 18 h under Ar atmosphere. The resulting bright yellow solution was then concentred under reduced pressure and a sat. solution of K_2_CO_3_ added. The resulting suspension was extracted with EtOAc (3 × 100 mL) and the resulting organic layer washed with H_2_O (2 × 100 mL) and brine (100 mL). The EtOAc solution was the dried over Na_2_SO_4_, filtered and evaporated under reduced pressure to afford **10** as a yellowish waxy solid (1.2 g, 92%).

^1^H NMR (400 MHz, CDCl_3_) δ: 7.73-7.70 (m, 2 H), 7.44 (d, *J* = 2.4 Hz, 1 H), 7.39 (t, *J* = 8.1, 1 H), 7.08 (d, *J* = 2.3 Hz, 1 H), 4.28 (t, *J* = 6.9 Hz, 4 H), 4.00 (bs, 2 H), 1.77-1.69 (m, 4 H), 1.49-1.39 (m, 4 H), 0.96 (t, *J* = 7.4 Hz, 3 H), 0.96 (t, *J* = 7.4 Hz, 3 H). ^13^C NMR (151 MHz, CDCl_3_) δ: 169.4, 168.95, 143.5, 136.3, 131.6, 130.5, 130.2, 126.8, 125.7, 122.2, 121.9, 112.3, 65.3, 65.2, 30.8, 30.7, 19.38, 19.36, 13.9. IR (ATR) ν (cm^-1^): 3451, 3362, 3244, 2959, 2932, 2895, 2872, 1694, 1641, 1614, 1576, 1512, 1474, 1466, 1433, 1396, 1375, 1308, 1281, 1265, 1244, 1173, 1150, 1126, 1082, 1063, 1026, 997, 974, 961, 937, 916, 901, 872, 845. ESI-HRMS: [M+H]^+^ calc. for [C_20_H_26_NO_4_Na]^+^ : 366.1676; found 366.1718.

## **2.8 Synthesis of 11**

In a round bottom flask, **10** (1.2 g, 3.49 mmol) was added and dispersed in dioxane (20 mL). To the resulting solution Br_2_ (188 μL, 3.67 mmol) was then added dropwise resulting in the formation of a faint yellow precipitate after ca. 30 min. The reaction was then diluted with PE, filtered and washed with PE. The faint yellow solid was then dispersed in CH_2_Cl_2_ (100 mL) and washed with a sat. aq. solution of K_2_CO_3_ followed by a sodium thiosulfate one (1 g in 100 mL), H_2_O (50 mL) and brine (50 mL). The organic layers were then dried over Na_2_SO_4_, filtered and evaporated. The obtained oily product was further purified by a short silica plug (PE/EtOAc 9/1 to 4/1) to give **11** as a brown viscous liquid (807 mg, 55%).

^1^H NMR (500 MHz, CDCl_3_) δ: 8.27 (dd, *J* = 8.6, 1.2 Hz, 1 H), 7.78 (dd, *J* = 7.2, 1.2 Hz, 1 H), 7.53 (dd, *J* = 8.6, 7.2 Hz, 1 H), 7.51 (s, 1 H), 4.28 (t, *J* = 6.8 Hz, 2 H), 4.27 (t, *J* = 6.8 Hz, 2 H), 1.76-1.69 (m, 4 H), 1.48-1.39 (m, 4 H), 0.96 (t, *J* = 7.4 Hz, 3 H), 0.95 (t, *J* = 7.4 Hz, 3 H). ^13^C NMR (126 MHz, CDCl_3_) δ: 169.0, 168.4, 141.7, 134.2, 130.8, 130.6, 129.5, 127.00, 126.99, 122.7, 121.4, 107.6, 65.5, 65.4, 30.8, 30.7, 19.38, 19.35, 13.9. IR (ATR) ν (cm^-1^): 3472, 3366, 2957, 2932, 2872, 1713, 1618, 1566, 1501, 1464, 1406, 1339, 1323, 1267, 1244, 1200, 1180, 1148, 1084, 1061, 1036, 937, 843, 816. ESI-HRMS: [M+Na]^+^ calc. for [C_20_H_24_NO_4_BrNa]^+^ : 444.0781; found 444.0782.

## **2.9 Synthesis of 12**

In a Schlenk tube **11** (750 mg, 1.78 mmol) was added along with Pd(OAc)_2_ (20 mg, 0.09 mmol), SPhos (74 mg, 0.18 mmol) and Cs_2_CO_3_ (1.16 g, 3.56 mmol). The solids were then placed under vacuum and the Schlenk tube filled with Ar. Anhydrous dioxane (25 mL) was then added and the resulting suspension degassed with Ar bubbling and sonication for 10 min. The reaction mixture was then placed in a preheated silicon oil bath at 110°C and stirred for 18 h. After cooling down, the solution was diluted with H_2_O (80 mL) and extracted with CH_2_Cl_2_ (3 × 100 mL). The organic layers were then washed with H_2_O (100 mL), brine (100 mL), dried over Na_2_SO_4_, filtered and evaporated under reduced pressure. The black residue was then purified using a silica plug (CH_2_Cl_2_ 100% to CH_2_Cl_2_/EtOAc 4/1) followed by reprecipitation/filtration from MeOH to give **12** as an amorphous yellow solid (198 mg, 33%).

^1^H NMR (400 MHz, CDCl_3_) δ: 9.71 (dd, *J* = 8.2, 1.4 Hz, 2 H), 8.69 (s, 2 H), 8.26 (dd, *J* = 7.4, 1.4 Hz, 2 H), 7.91 (dd, *J* = 8.1, 7.4 Hz, 2 H), 4.42 (t, *J* = 6.8 Hz, 4 H), 4.37 (t, *J* = 6.8 Hz, 4 H), 1.89-1.77 (m, 8 H), 1.60-1.46 (m, 8 H), 1.03 (t, *J* = 7.4 Hz, 6 H), 1.00 (t, *J* = 7.4 Hz, 6 H). ^13^C NMR (101 MHz, CDCl_3_) δ 168.8, 168.0, 143.1, 141.3, 134.6, 132.6, 132.1, 131.7, 131.3, 128.7, 127.9, 127.7, 65.9, 65.7, 30.81, 30.78, 19.44, 19.42, 13.98, 13.95. IR (ATR) ν (cm^-1^): 2959, 2934, 2909, 2874, 2847, 1713, 1489, 1464, 1425, 1393, 1356, 1298, 1277, 1250, 1207, 1194, 1157, 1142, 1094, 1078, 1059, 1018, 999, 962, 943, 901, 872, 831. ESI-HRMS: [M+H]^+^ calc. for [C_40_H_45_N_2_O_8_]^+^ : 681.3170; found 681.3113.

## **2.10 Synthesis of 13**

In a round bottom flask, **12** (100 mg, 0.147 mmol) was added and suspended in toluene (5 mL). PTSA∙H_2_O (140 mg, 0.73 mmol) was then added, and the resulting suspension refluxed for 18 h. The resulting yellow suspension was then allowed to cool to r.t., diluted with MeOH and centrifuged. After removing the liquids this procedure was repeated once more resulting in **13** as a yellow insoluble powder (55 mg, 89%).

IR (ATR) ν (cm^-1^): 3067, 1775, 1751, 1624, 1601, 1506, 1433, 1381, 1358, 1344, 1277, 1221, 1196, 1161, 1144, 1113, 1082, 1038, 993, 947, 918, 883, 864, 843.

## **2.11 Synthesis of 1c**

In a microwave vial, **13** (25 mg, 0.06 mmol) was added and suspended in DMF (1.0 mL). 1-Decanamine (26 μL, 0.131 mmol) was then added, and the resulting suspension heated under MW irradiation at 130°C for 30 min. The resulting suspension was the diluted with MeOH and centrifuged. After removal of the liquid phase the solids were again dispersed in MeOH and the procedure repeated twice to give **1c** as a yellow solid (30 mg, 72%).

M.P.:285-286 °C. ^1^H NMR (500 MHz, CDCl_3_) δ: 9.72 (d, *J* = 8.1 Hz, 2 H), 9.29 (s, 2 H), 8.83 (d, *J* = 7.2 Hz, 2 H), 8.12 (t, *J* = 7.8 Hz, 2 H), 4.27-4.24 (m, 4 H), 1.84-1.78 (m, 4 H), 1.51-1.45 (m, 4 H), 1.43-1.37 (m, 4 H), 1.34-1.27 (m, 20 H), 0.88 (t, *J* = 6.7 Hz, 6 H). Molecule is not soluble enough to record ^13^C NMR. IR (ATR) ν (cm^-1^): 3057, 2951, 2916, 2847, 1703, 1661, 1622, 1599, 1557, 1504, 1466, 1456, 1443, 1427, 1391, 1358, 1339, 1288, 1279, 1265, 1246, 1238, 1227, 1217, 1198, 1171, 1157, 1132, 1094, 1067, 1049, 1034, 1024, 999, 984, 974, 951, 920, 912, 887, 854, 839. MALDI-LRMS: [M+H]^+^ calc. for [C_44_H_51_N_4_O_4_]^+^ : 699.391; found 699.492. λ_abs(max)_= 441 nm, λ_em(max)_= 447 nm (exc = 400 nm) in CHCl_3_.

## **2.12 Synthesis of 1d**

In a microwave vial, **13** (10 mg, 0.024 mmol) was added and suspended in DMF (1.0 mL). N,N-dimethylethylendiamine (5.7 μL, 0.052 mmol) was then added, and the resulting suspension heated under MW irradiation at 130°C MW for 30 min. The resulting suspension was the diluted with Et_2_O and centrifuged. After removal of the liquid phase the solids were again dispersed in Et_2_O and the procedure repeated twice to give **1d** as a yellow solid (11 mg, 82%).

MP.: >300°C. ^1^H NMR (500 MHz, TFA-*d*1) δ: 9.88 (d, *J* = 8.2 Hz, 2 H), 9.50 (s, 2 H), 8.93 (d, *J* = 7.6 Hz, 2 H), 8.29 (t, *J* = 7.6 Hz, 2 H), 4.81-4.76 (m, 4 H), 3.79-3.74 (m, 4 H), 3.19 (s, 12 H). ^13^C NMR (126 MHz, TFA-*d*1) δ: 168.4, 167.3, 144.8, 143.0, 136.8, 135.5, 135.1, 133.1, 130.6, 130.4, 129.6, 124.1, 60.4, 46.3, 38.7. IR (ATR) ν (cm^-1^): 3092, 2951, 2818, 2764, 1699, 1659, 1622, 1599, 1504, 1443, 1422, 1383, 1369, 1360, 1335, 1283, 1240, 1161, 1138, 1105, 1082, 1069, 1053, 1022, 991, 935, 914, 883, 833. ESI-HRMS: [M+H]^+^ calc. for [C_32_H_29_N_6_O_4_]^+^ : 561.2245; found 561.2248.

## **2.13 Synthesis of 1e**

In a microwave vial, **13** (50 mg, 0.12 mmol) was added and suspended in DMF (5.0 mL). Pentadecan-8-amine (81 mg, 0.36 mmol) was then added, and the resulting suspension heated under MW irradiation at 130°C for 1 h. The resulting suspension was the diluted with MeOH and centrifuged. After removal of the liquid phase the solids were again dispersed in MeOH, and the procedure repeated. The resulting material was purified on a short silica plug (CH_2_Cl_2_/PE 3/7) to give **1e** as an amorphous yellow solid (47 mg, 47%).

^1^H NMR (400 MHz, CDCl_3_) δ: 9.66 (d, *J* = 8.1 Hz, 2 H), 9.27 (bs, 2 H), 8.79 (bs, 2 H), 8.09 (t, *J* = 7.8 Hz, 2 H), 5.28-5.20 (m, 2 H), 2.32-2.26 (m, 4 H), 1.94-1.87 (m, 4 H), 1.40-1.22 (m, 40 H), 0.82 (m, 12 H). ^13^C NMR (101 MHz, CDCl_3_) δ: 165.1, 164.4, 164.0, 163.2, 143.6, 142.3, 134.4, 133.7, 132.9, 132.1, 130.7, 129.7, 129.2, 128.3, 127.5, 126.9, 124.0, 123.3, 55.3, 32.5, 31.9, 29.8, 29.7, 29.4, 27.1, 22.8, 14.2.*More carbon signals are present due to the presence slowed C-N rotation in swallowtailed diimide derivatives resulting in the presence of different rotamers.^[6]^ IR (ATR) ν (cm^-1^): 2953, 2922, 2853, 1705, 1661, 1624, 1601, 1505, 1466, 1439, 1398, 1360, 1331, 1294, 1283, 1234, 1217, 1167, 1144, 1126, 1099, 1069, 1032, 918, 785, 747, 721. ESI-HRMS: [M+H]^+^ calc. for [C_54_H_71_N_4_O_4_]^+^ : 839.5470; found 839.5470. λ_abs(max)_= 442 nm, _em(max)_= 446 nm (exc = 400 nm) in THF.

**2.13a Reduction procedure of 1e using Na_2_S_2_O_4_**

In a glass vial with a pierceable rubber cap, fresh THF-*d8* and D_2_O were added (0.6 + 0.2 mL) together with **1e** (5 mg, 0.006 mmol) and a large excess of Na_2_S_2_O_4_ (to saturation). The resulting biphasic system was then degassed for 1 min. with Ar bubbling and sonication (the solution turns immediately purple). Separately an NMR tube was filled with Ar using a balloon and a 12 cm needle, and maintaining Ar insufflation the solution was quickly transferred from the vial to the NMR tube. After 1 more minute of Ar insufflation the NMR tube was sealed with a plastic cap and parafilm. The sample was then employed for the reported experiments. **1e^2H^** is sensitive to air and gets oxidized to **1e** in minutes if exposed to air in absence of excess reductant.

**2.13b Reduction procedure of 1e using Et_3_SiH and Pd(OAc)_2_**

In a glass vial with a pierceable rubber cap, fresh THF-*d8* was added (0.75 mL) together with **1e** (5 mg, 0.006 mmol) and a catalytic amount of Pd(OAc)_2_ (touching the tip of a Pasteur pipette in the catalyst and then placing it in the solution is sufficient). The resulting solution was then degassed for 1 min. with Ar bubbling and sonication followed by addition of an excess of Et_3_SiH (15 eq, ca. 15 μL) (the solution turns immediately purple), the vial immediately sealed and further degassed with Ar and sonication for 1 min. (beware of THF and Et_3_SiH evaporation). Separately an NMR tube was filled with Ar using a balloon and a 12 cm needle, and maintaining Ar insufflation the solution was quickly transferred with a syringe and needle from the vial to the NMR tube. After 1 more minute of Ar insufflation the NMR tube was sealed with a plastic cap and parafilm. The sample was then employed for the reported experiments. **1e^2H^** is sensitive to air and gets oxidized to **1e** in minutes if exposed to air in absence of excess reductant.

**2.13c Reduction procedure of 1e using TBABH_4_**

In a glass vial with a pierceable rubber cap, fresh THF-*d8* was added (1.0 mL) together with **1e** (10 mg, 0.012 mmol) and the resulting solution degassed for 1 min. with Ar bubbling and sonication. At this point TBABH_4_ (9 mg, 0.035 mmol) was added in one portion (the solution turns immediately deep blue), the vial immediately sealed and degassed with Ar and sonication for further 1 min. (beware of THF evaporation). Separately an NMR tube was filled with Ar using a balloon and a 12 cm needle, and maintaining Ar insufflation the solution was quickly transferred from the vial to the NMR tube. After 1 more minute of Ar insufflation the NMR tube was sealed with a plastic cap and parafilm. The sample was then employed for the reported experiments. **1e^2H-^** is highly sensitive to air and gets degraded immediately if exposed to air.

## **2.14 Synthesis of 1f**

In a microwave vial, **13** (25 mg, 0.06 mmol) was added along with g-amino butyric acid (14 mg, 0.13 mmol), NEt_3_ (42 μL, 0.3 mmol) and suspended in DMF (1.0 mL). The resulting suspension was then heated under MW irradiation at 130°C for 1 h. The resulting suspension was the diluted with 50 mM Cs_2_CO_3_, the solution acidified with HCl 3M until precipitation occurs and then centrifuged. After removal of the liquid phase the solids were again centrifuged from H_2_O, MeOH and Et_2_O. The resulting material was dried to give **1f** as an amorphous yellow solid (24 mg, 68%).

^1^H NMR (400 MHz, D_2_O/MeOD 1/1 Cs_2_CO_3_ 50 mM) δ: 8.41 (bs, 2 H), 8.09 (bs, 2 H), 7.68-7.63 (m, 4 H), 3.80 (bs, 4 H), 2.31 (t, *J* = 7.5 Hz, 4 H), 1.91-1.87 (m, 4 H). Not soluble enough for ^13^C NMR. ESI-HRMS: [M-H]^-^ calc. for [C_32_H_21_N_4_O_8_]^-^: 589.1365; found 589.1363. λ_abs(max)_= 422 nm in H_2_O Cs_2_CO_3_ 50 mM.

# **3 NMR and HRMS spectroscopic characterization**

## **3.1 Characterization of 6**

**
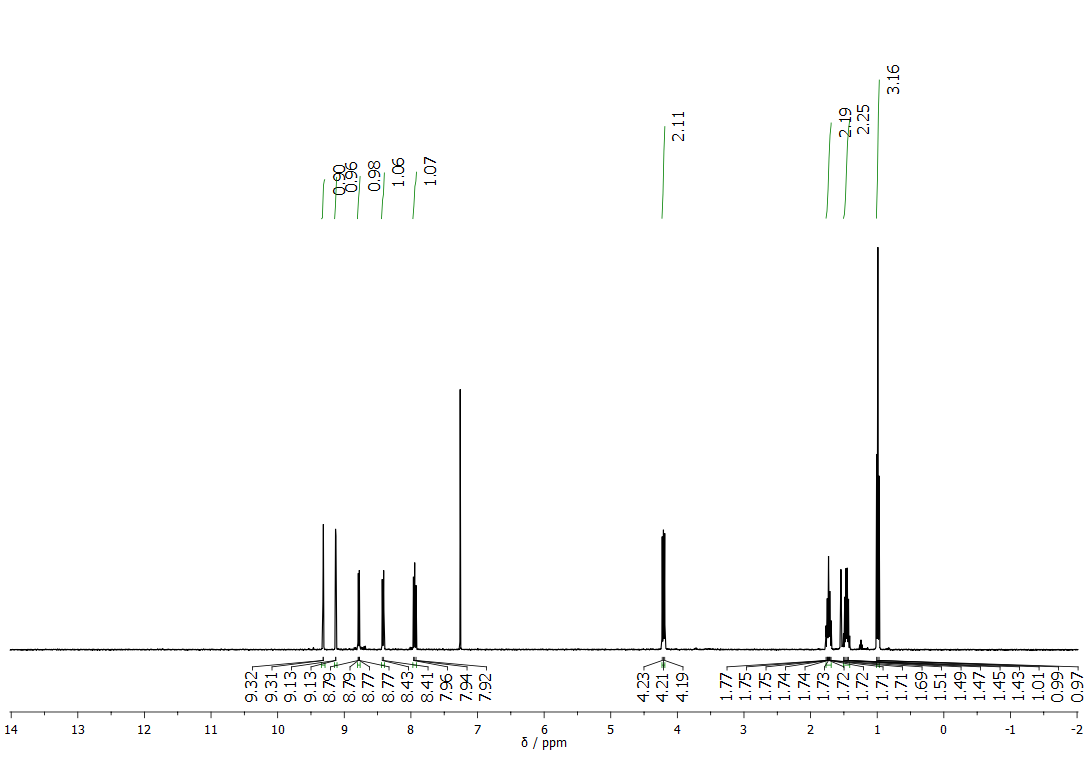
**

**Figure S1**. ^1^H NMR 400 MHz of **6** in CDCl_3_.


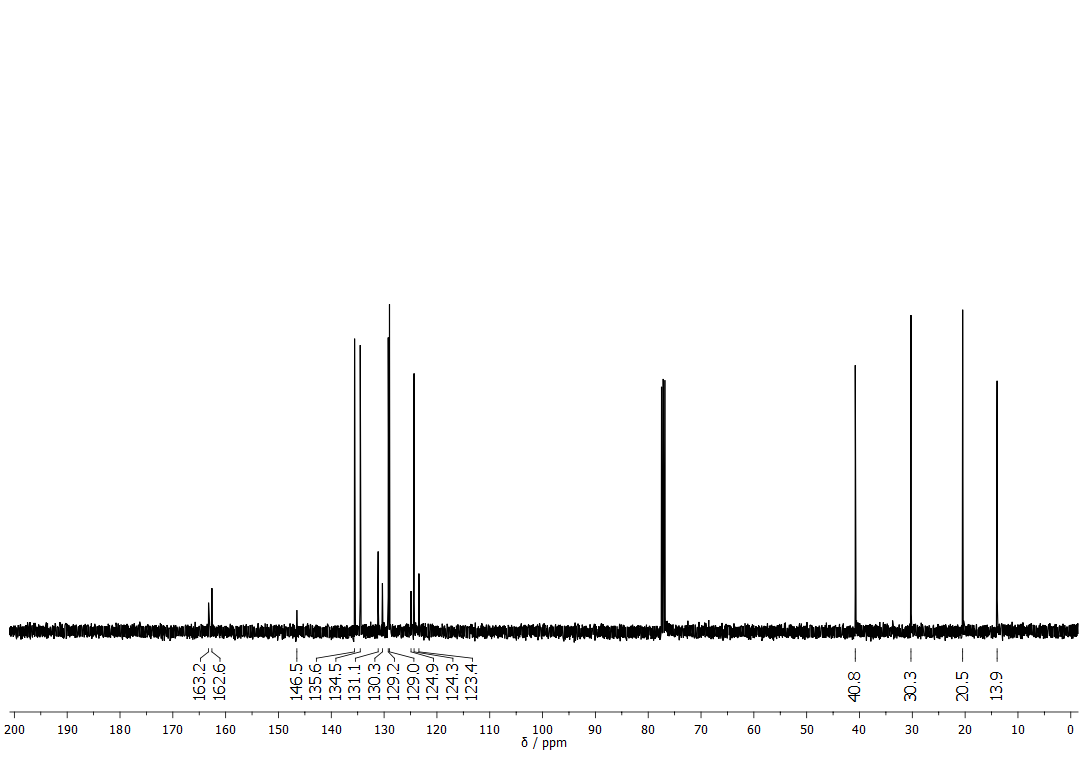


**Figure S2**. ^13^C NMR 101 MHz of **6** in CDCl_3_.


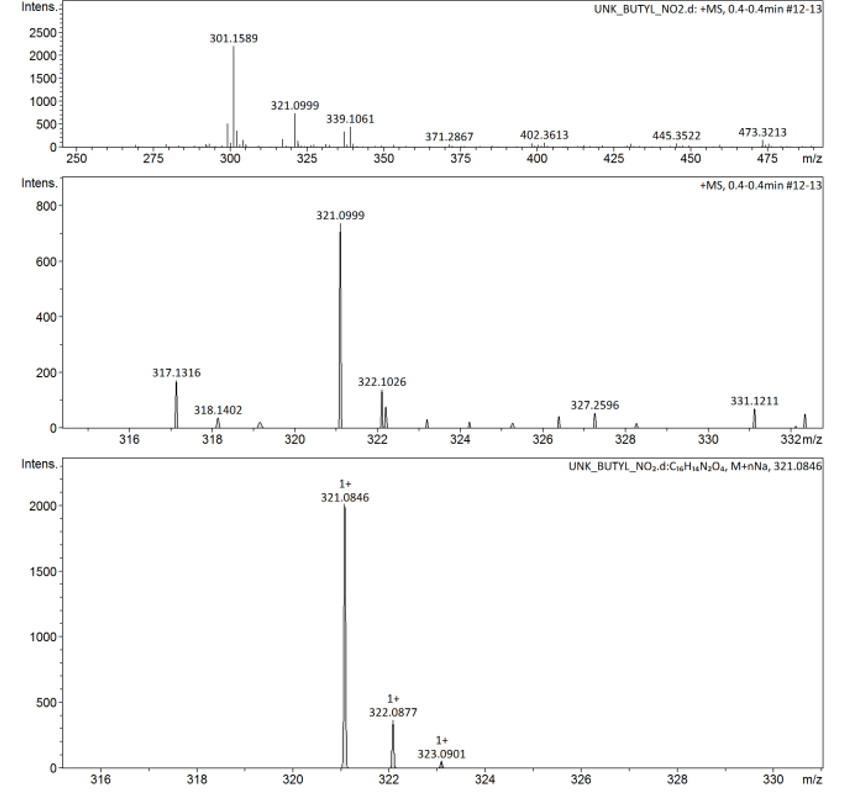


**Figure S3**. ESI-HRMS of **6**, top) experimental spectra, middle) zoom and bottom) simulated spectra.

## **3.2 Characterization of 7**


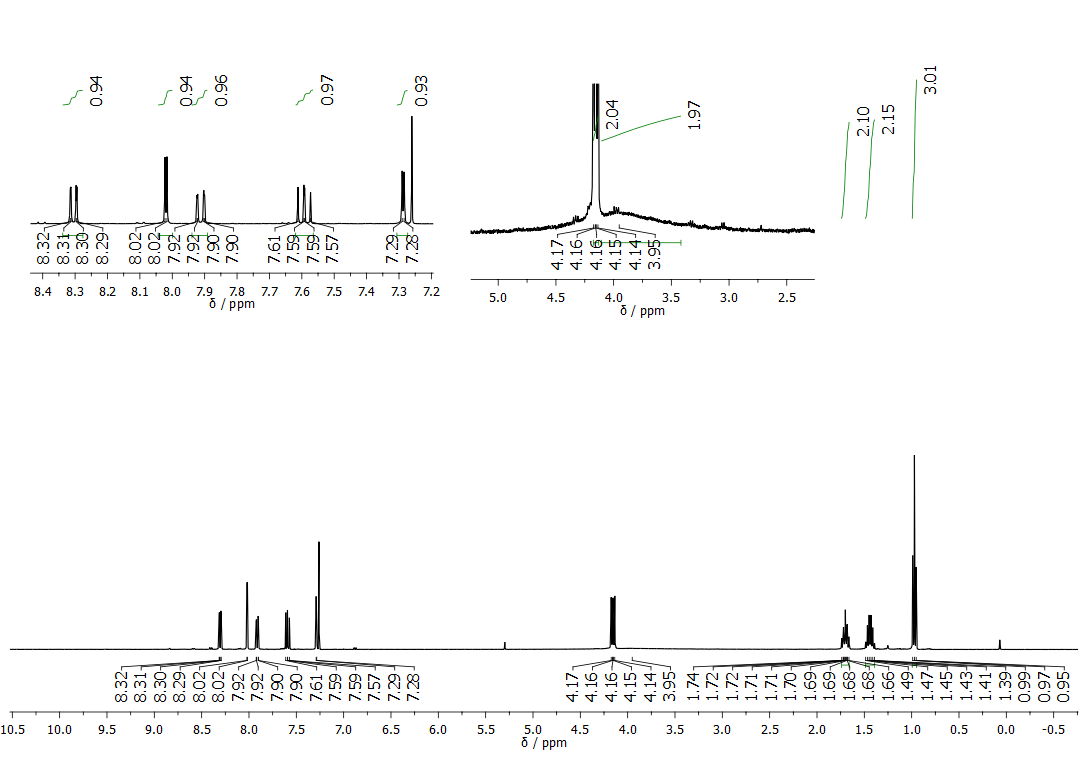


**Figure S4**. ^1^H NMR 400 MHz of **7** in CDCl_3_.


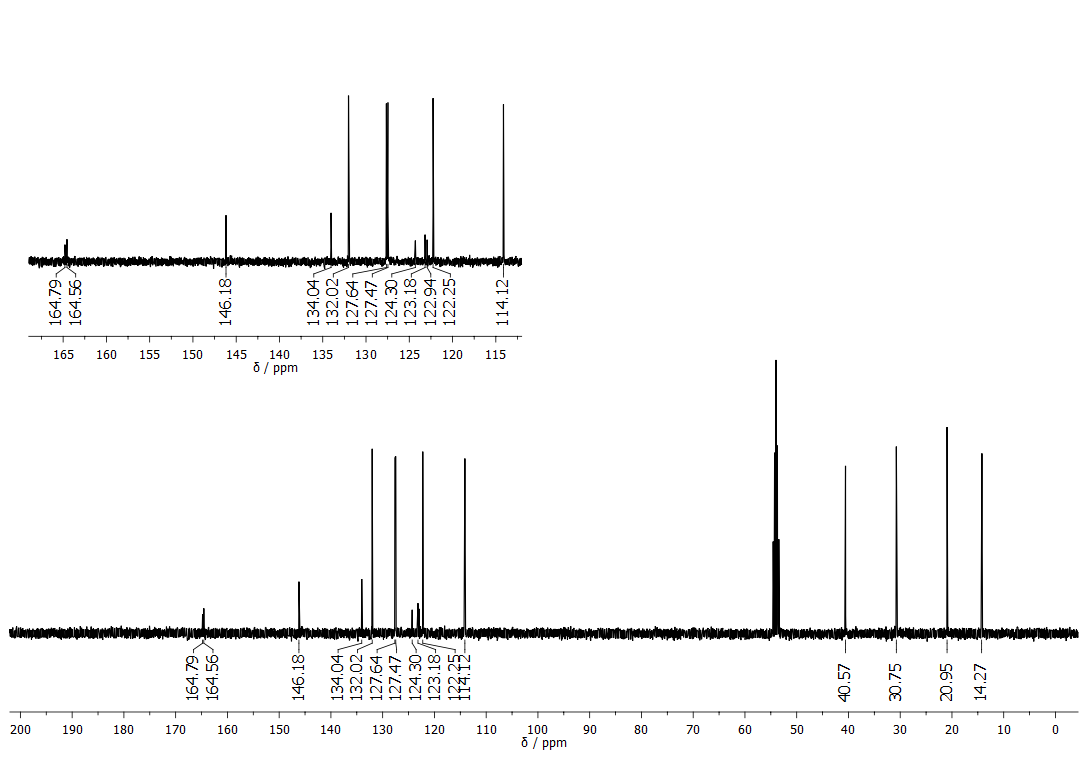


**Figure S5**. ^13^C NMR 101 MHz of **7** in CD_2_Cl_2_.


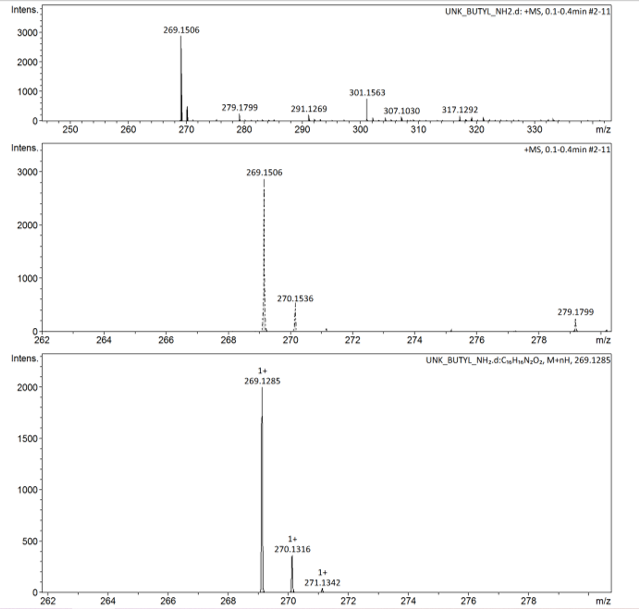


**Figure S6**. ESI-HRMS of **7,** top) experimental spectra, middle) zoom and bottom) simulated spectra.

## **3.3 Characterization of 8**


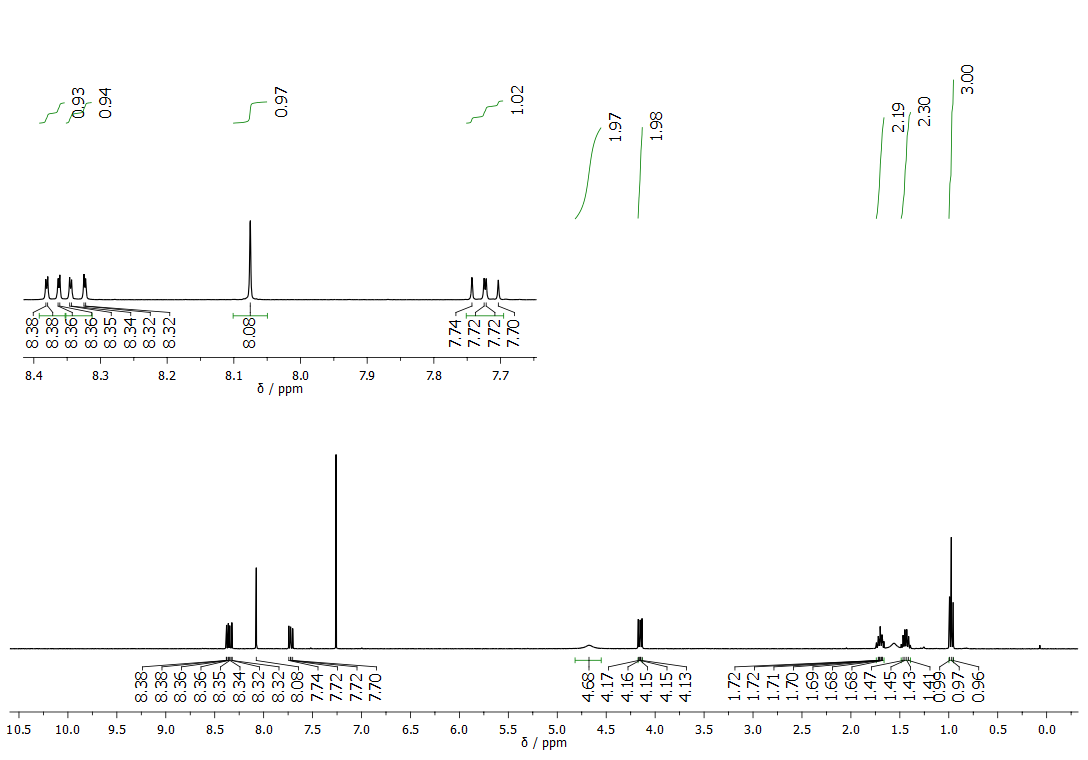


**Figure S7**. ^1^H NMR 400 MHz of **8** in CDCl_3_.

**
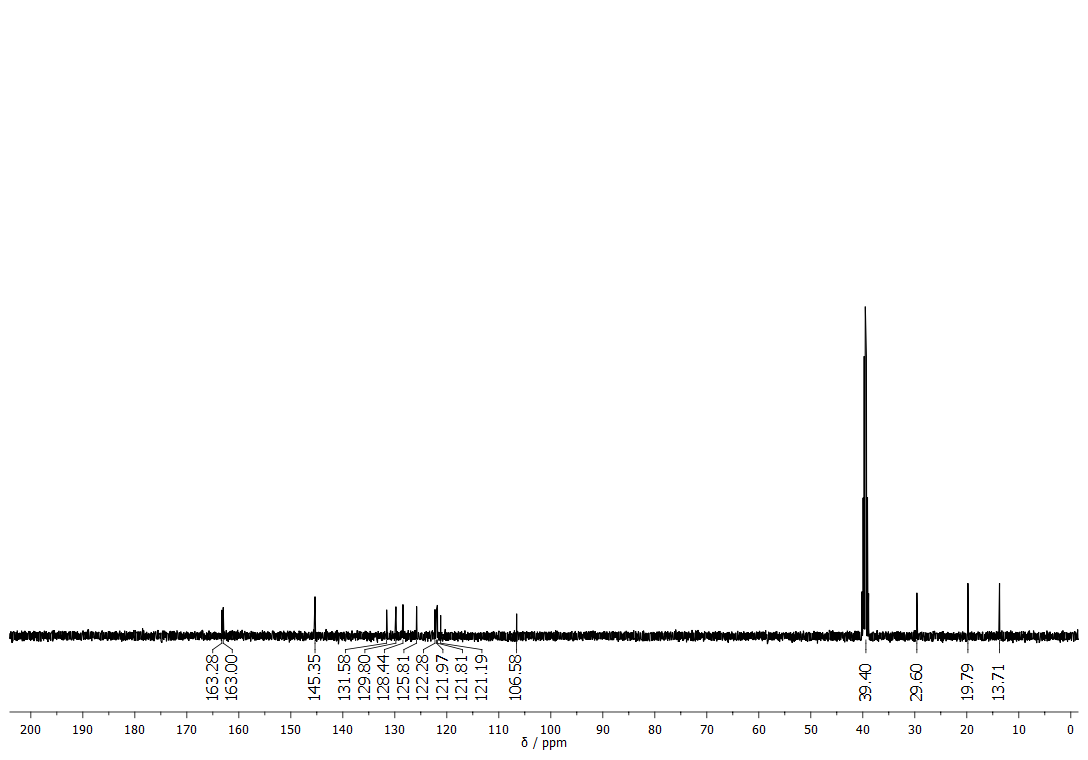
**

**Figure S8**. ^13^C NMR 101 MHz of **8** in DMSO.

**
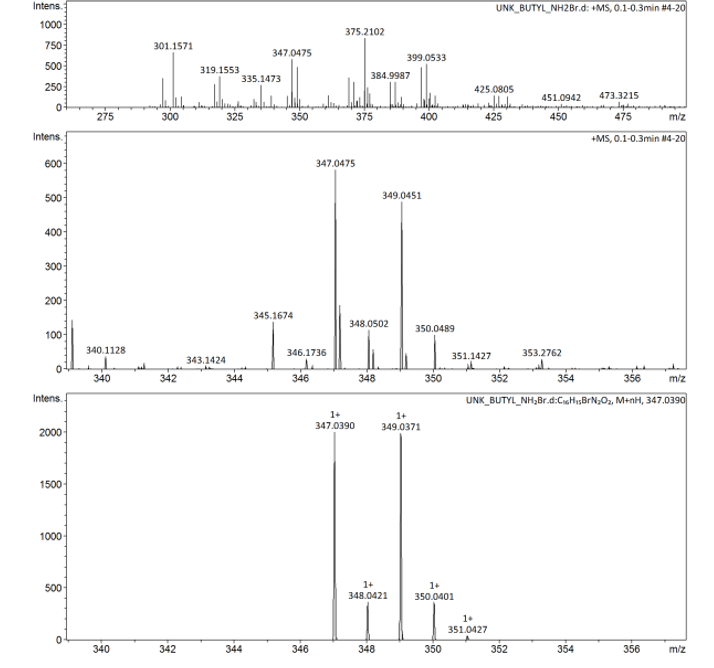
**

**Figure S9**. ESI-HRMS of **8**, top) experimental spectra, middle) zoom and bottom) simulated spectra.

## **3.4 Characterization of 1b**


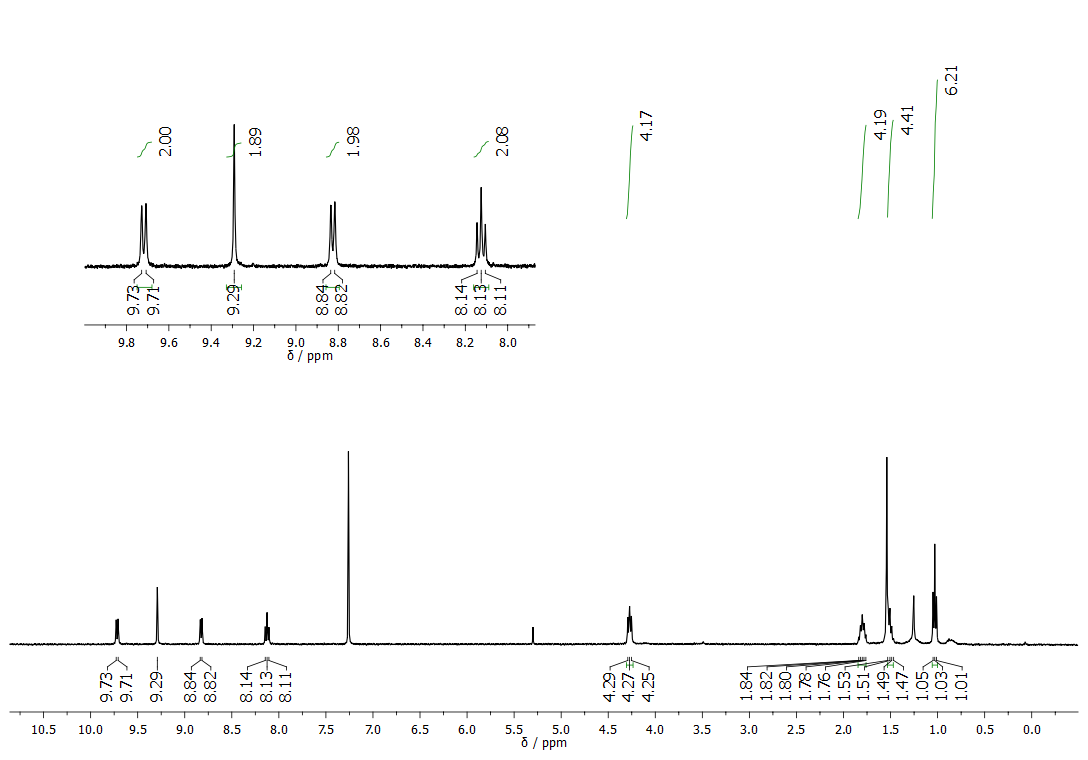


**Figure S10**. ^1^H NMR 400 MHz of **1b** in CDCl_3_ (signal at 5.30 is due to residual CH_2_Cl_2_ traces, signal at 1.25 is ascribable to grease residuals).


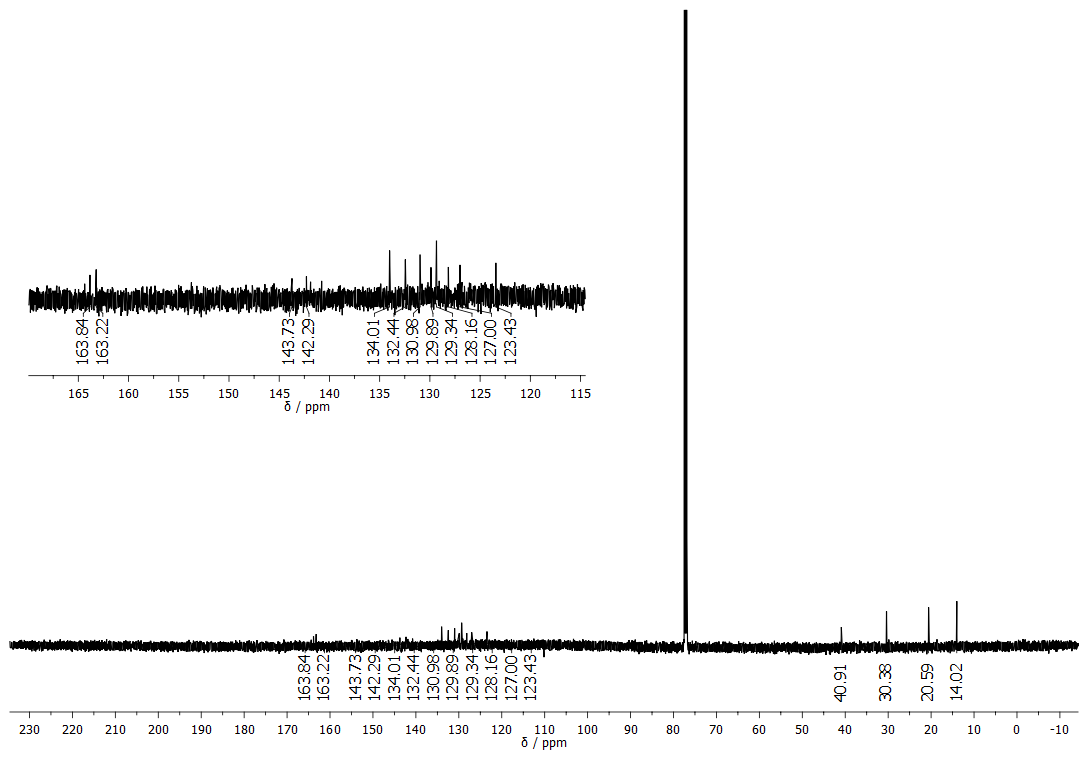


**Figure S11**. ^13^C NMR 126 MHz of **1b** in CDCl_3._

_
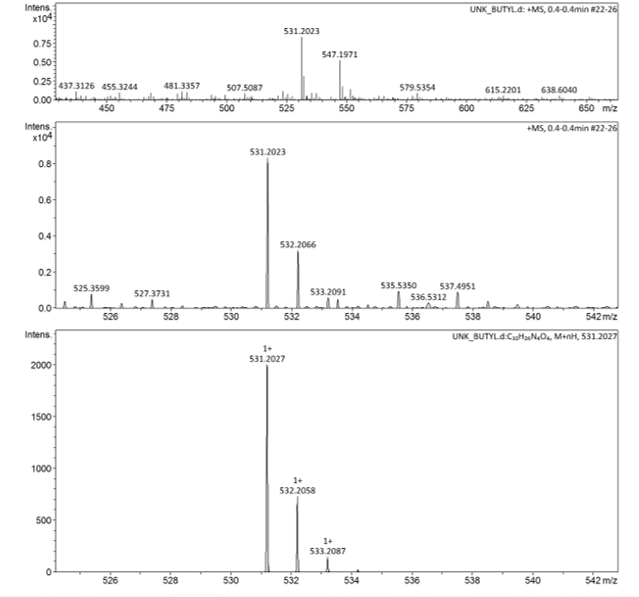
_

**Figure S12**. ESI-HRMS of **1b**, top) experimental spectra, middle) zoom and bottom) simulated spectra.

## **3.5 Characterization of 1a**


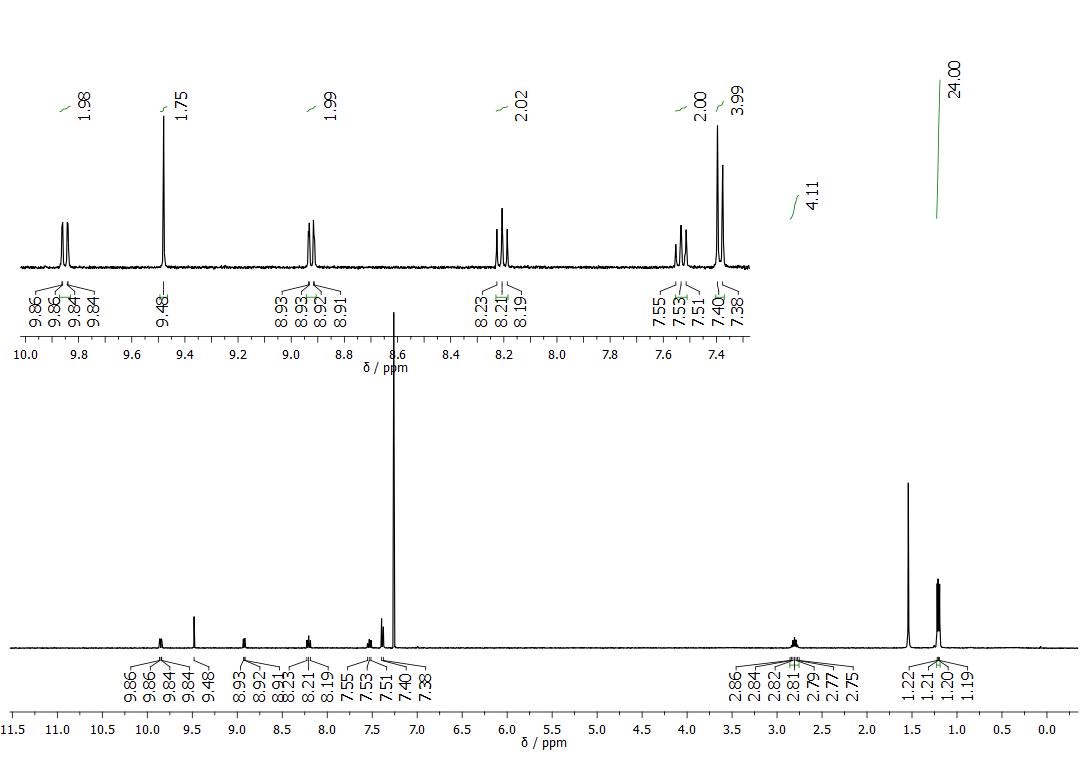


**Figure S13**. ^1^H NMR 400 MHz of **1a** in CDCl_3_


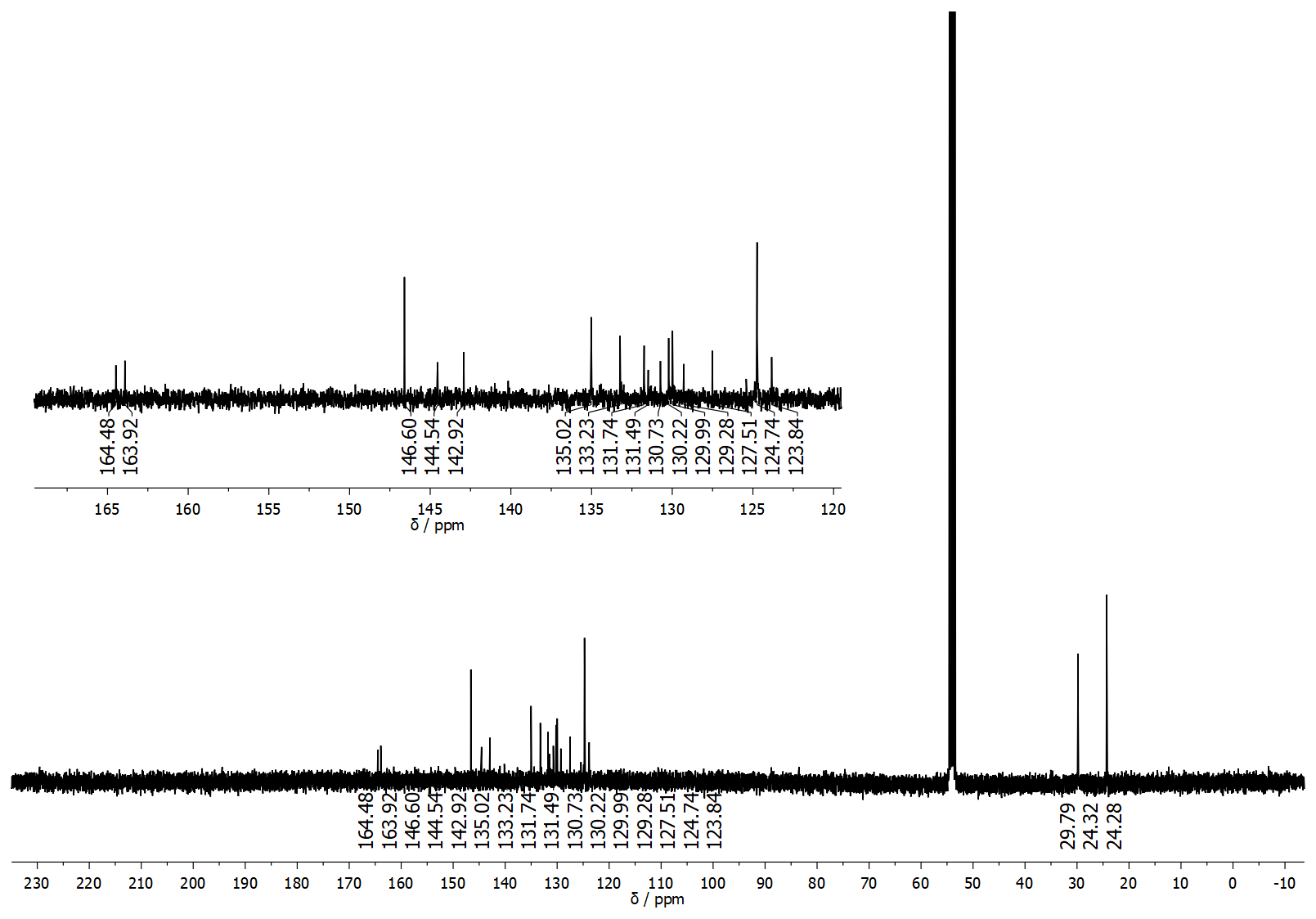


**Figure S14**. ^13^C NMR 101 MHz of **1a** in CD_2_Cl_2._


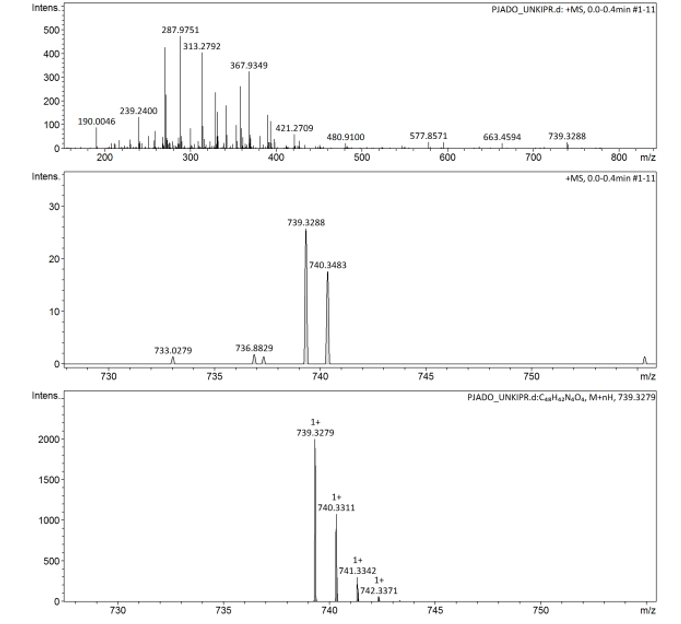


**Figure S15**. ESI-HRMS of **1a**, top) experimental spectra, middle) zoom and bottom) simulated spectra. Low intensity signal is due to reduced solubility in ESI solvents.

## **3.6 Characterization of 9**


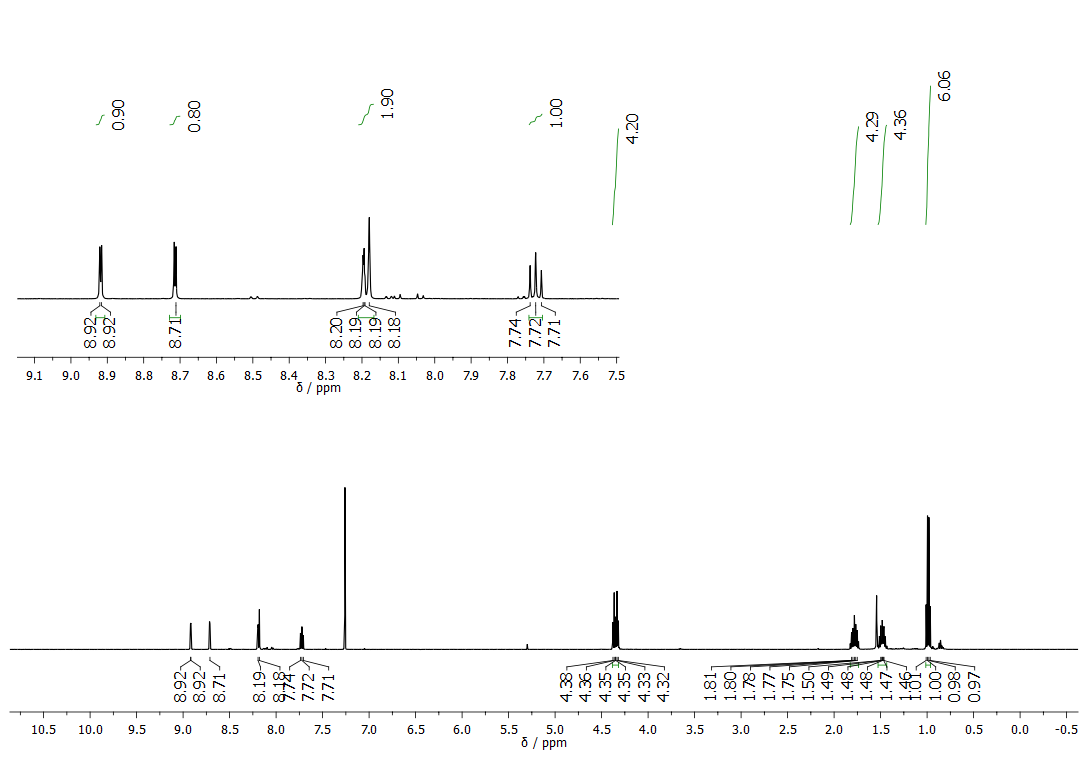


**Figure S16**. ^1^H NMR 500 MHz of **9** in CDCl_3_.


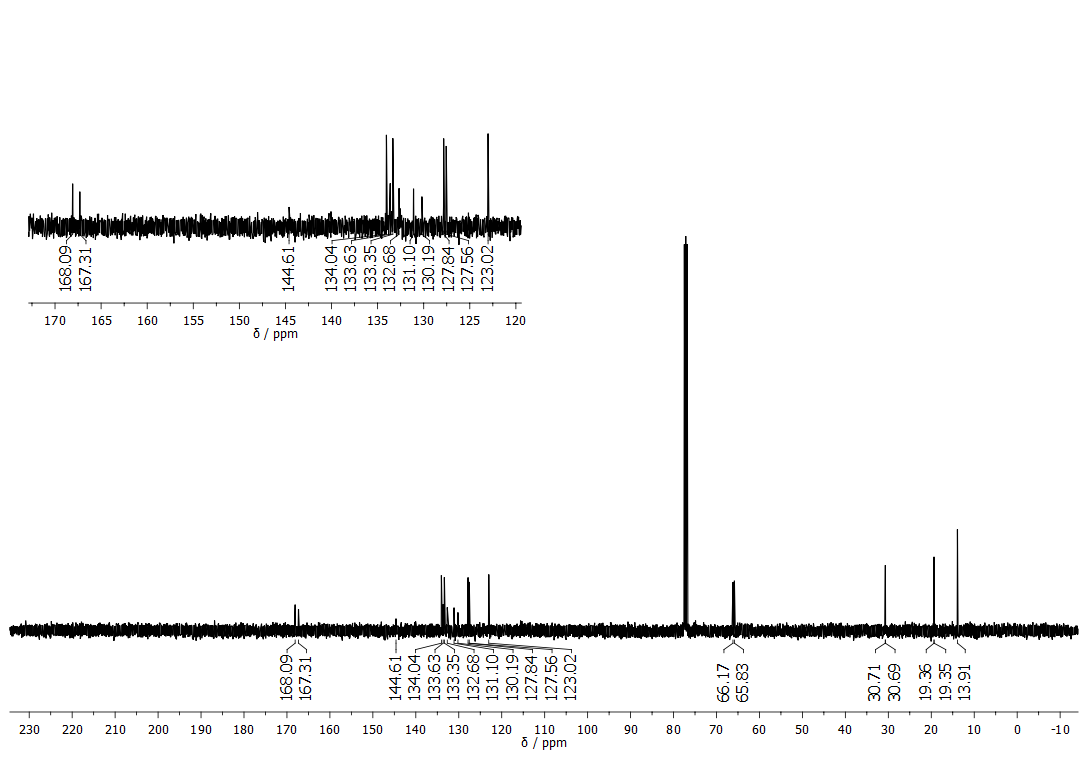


**Figure S17**. ^13^C NMR 101 MHz of **9** in CDCl_3._

_
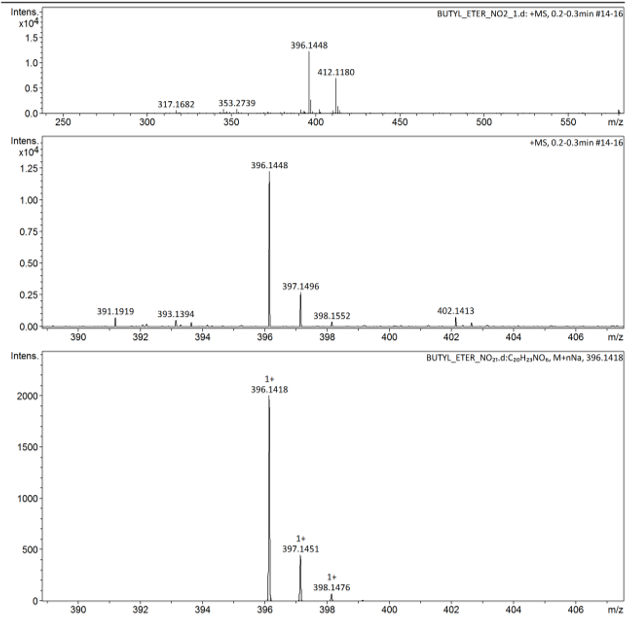
_

**Figure S18**. ESI-HRMS of **9**, top) experimental spectra, middle) zoom and bottom) simulated spectra.

## **3.7 Characterization of 10**


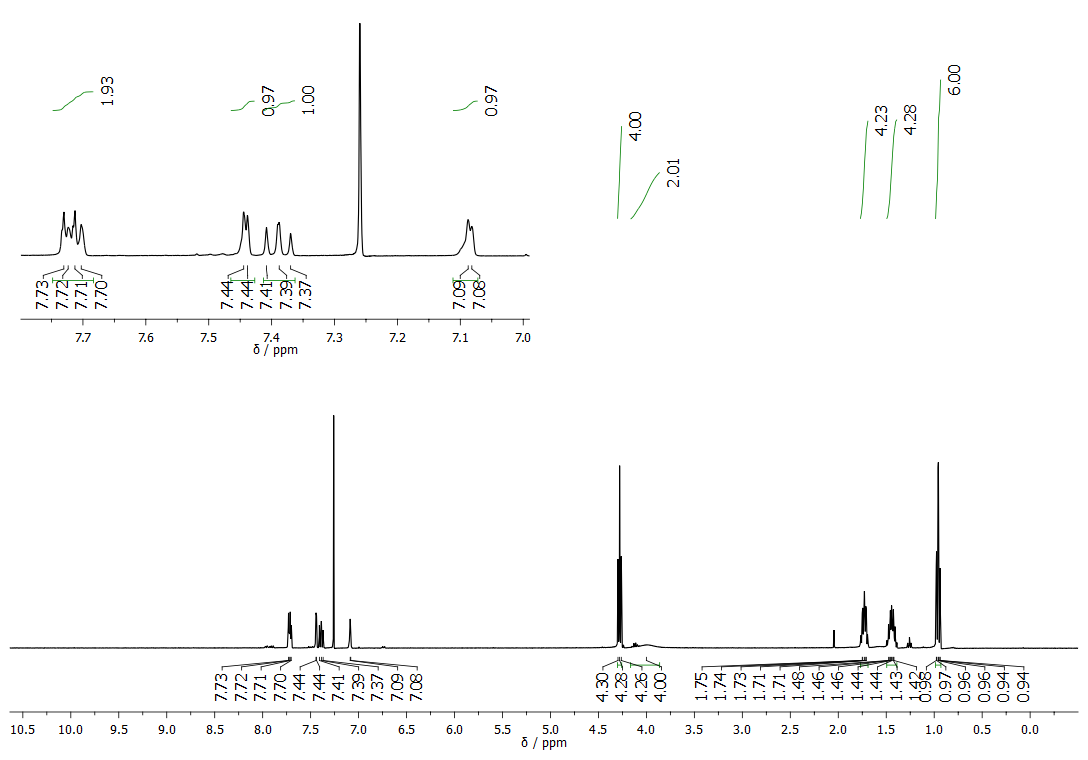


**Figure S19**. ^1^H NMR 400 MHz of **10** in CDCl_3_.


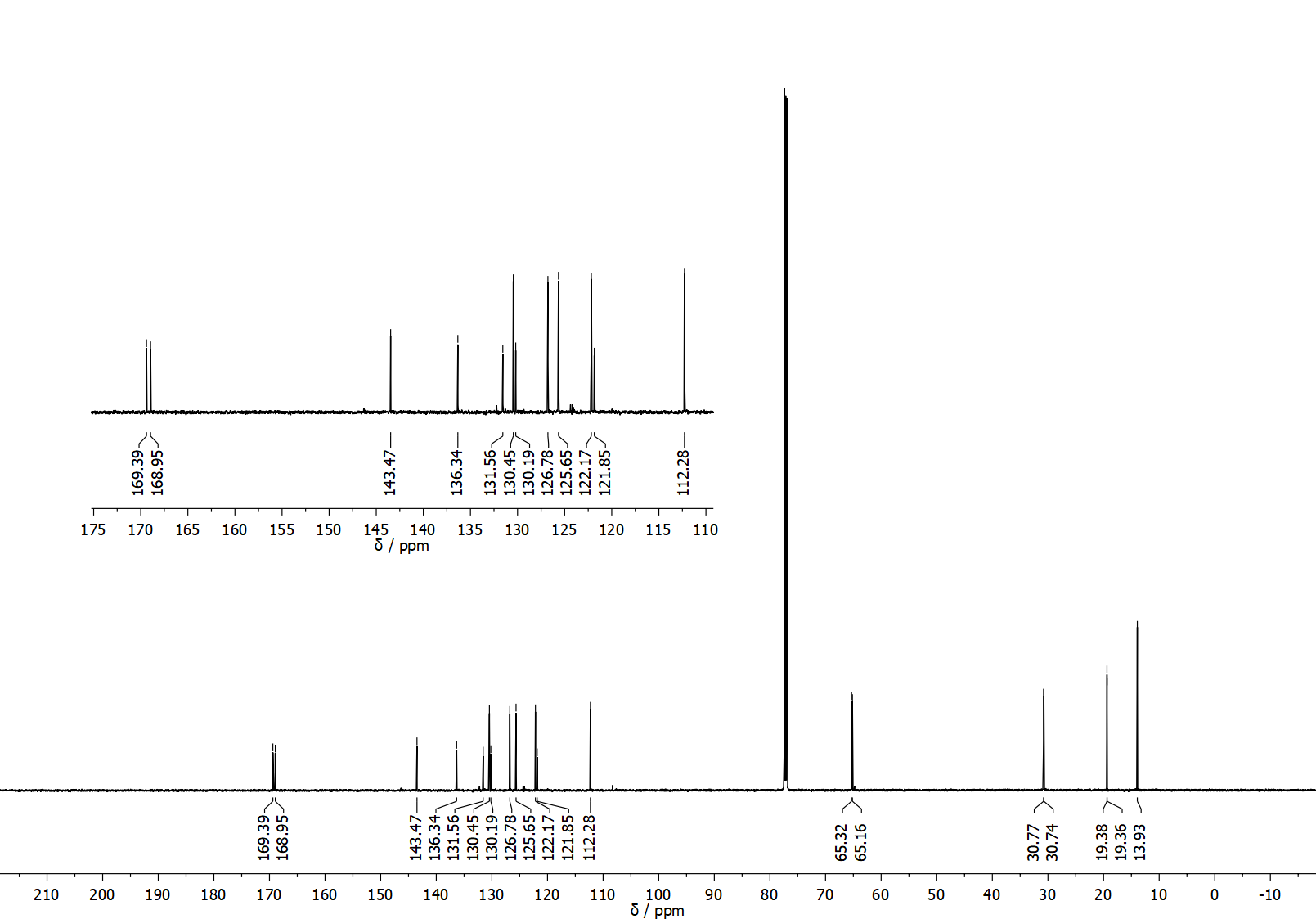


**Figure S20**. ^13^C NMR 151 MHz of **10** in CDCl_3._


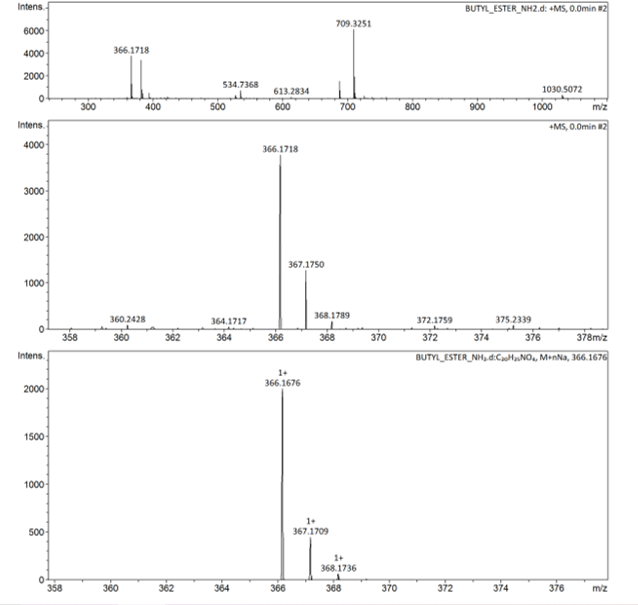


**Figure S21**. ESI-HRMS of **10**, top) experimental spectra, middle) zoom and bottom) simulated spectra.

## **3.8 Characterization of 11**


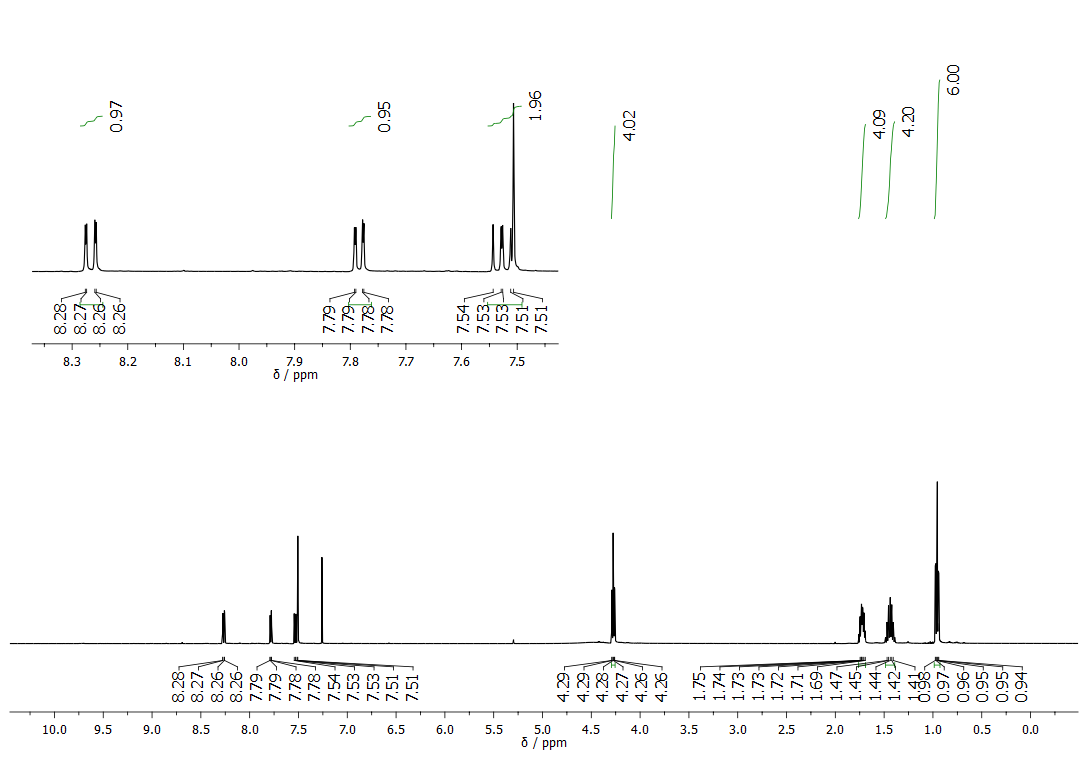


**Figure S22**. ^1^H NMR 400 MHz of **11** in CDCl_3_.


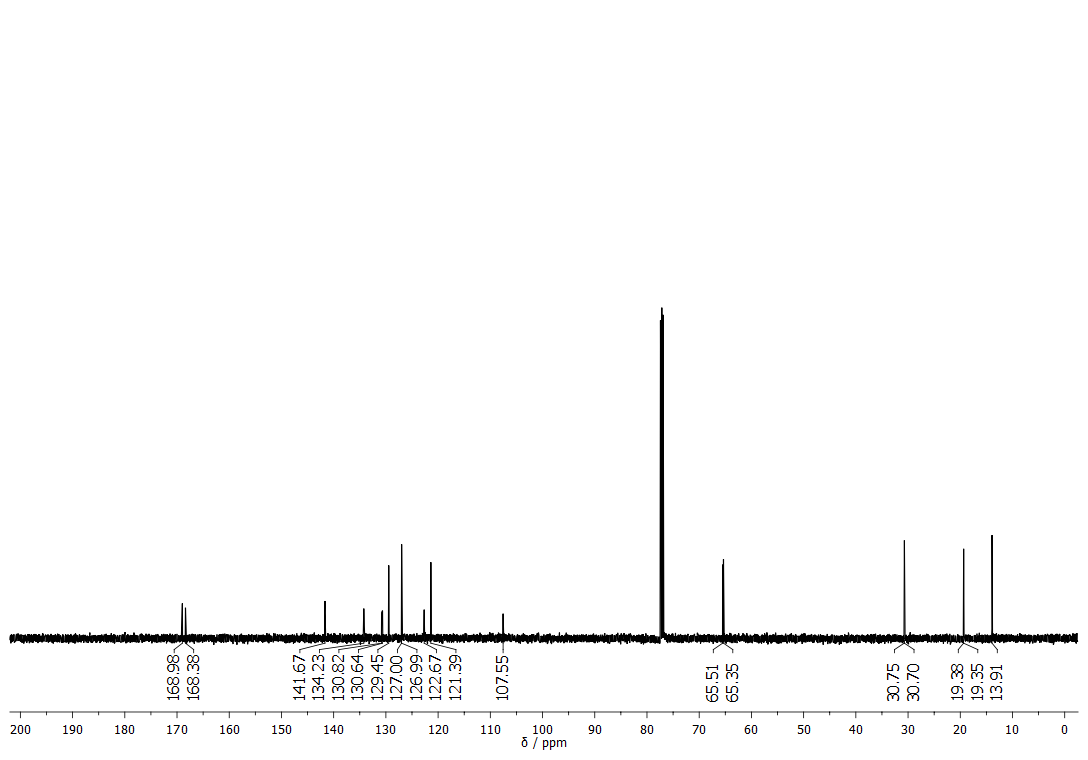


**Figure S23**. ^13^C NMR 126 MHz of **11** in CDCl_3._


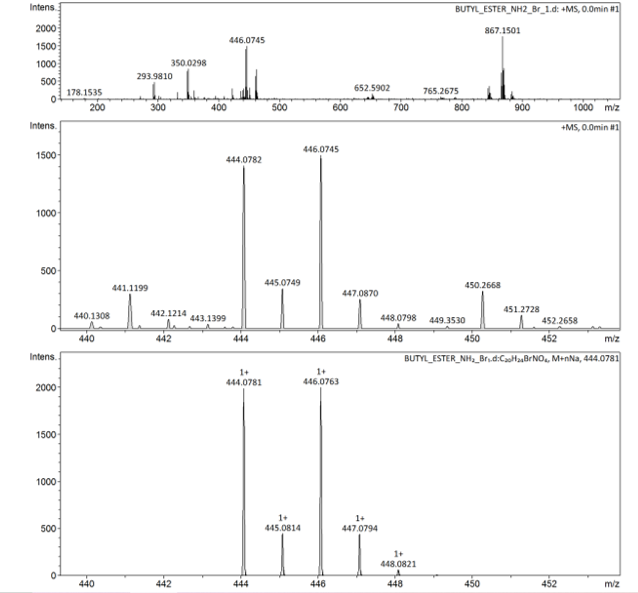


**Figure S24**. ESI-HRMS of **11**, top) experimental spectra, middle) zoom and bottom) simulated spectra.

## **3.9 Characterization of 12**


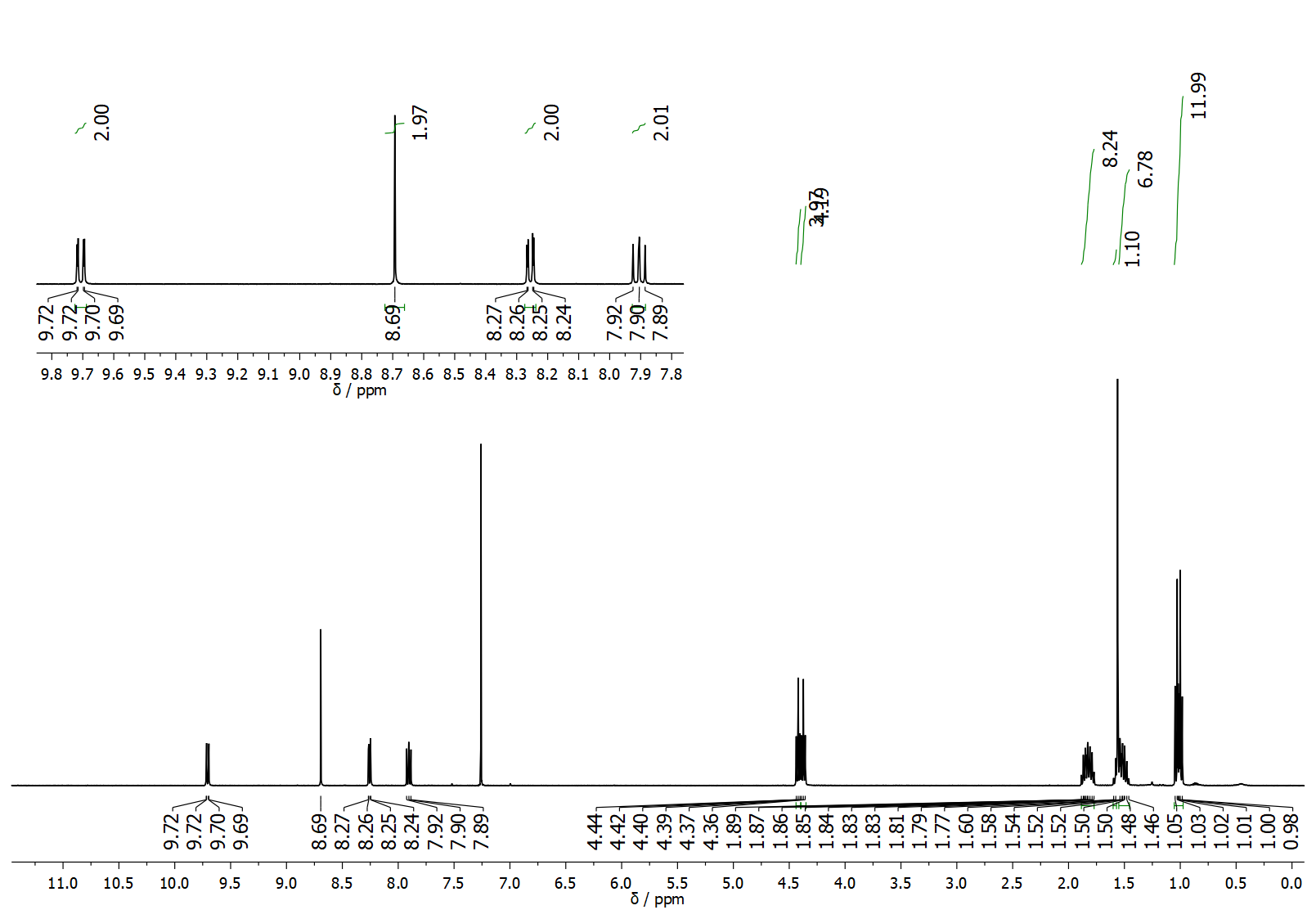


**Figure S25**. ^1^H NMR 400 MHz of **12** in CDCl_3_


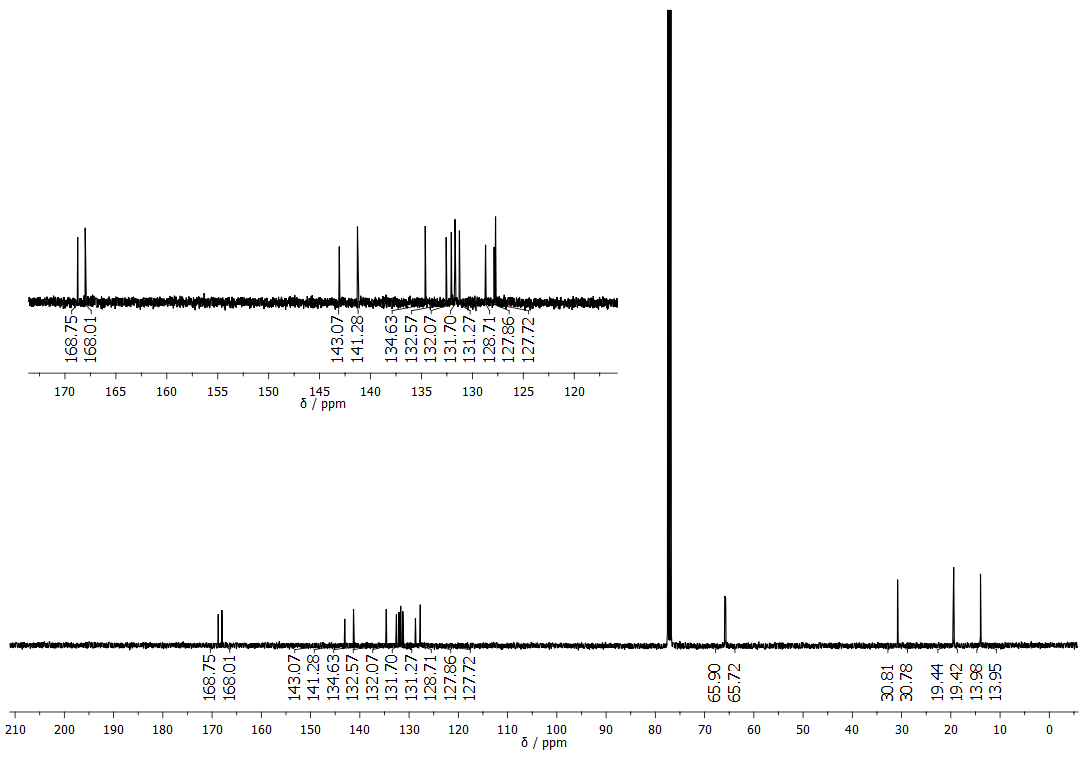


**Figure S26**. ^13^C NMR 101 MHz of **12** in CDCl_3._


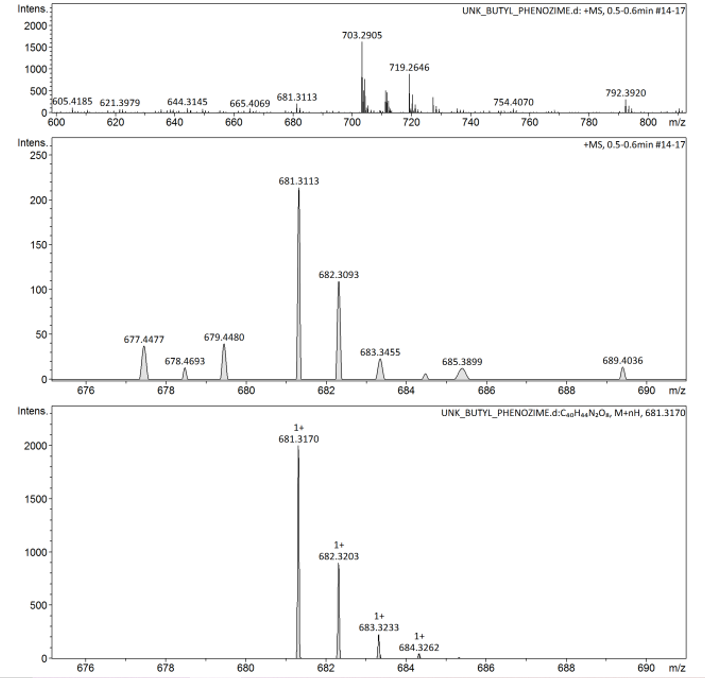


**Figure S27**. ESI-HRMS of **12**, top) experimental spectra, middle) zoom and bottom) simulated spectra.

## **3.10 Characterization of 1c**


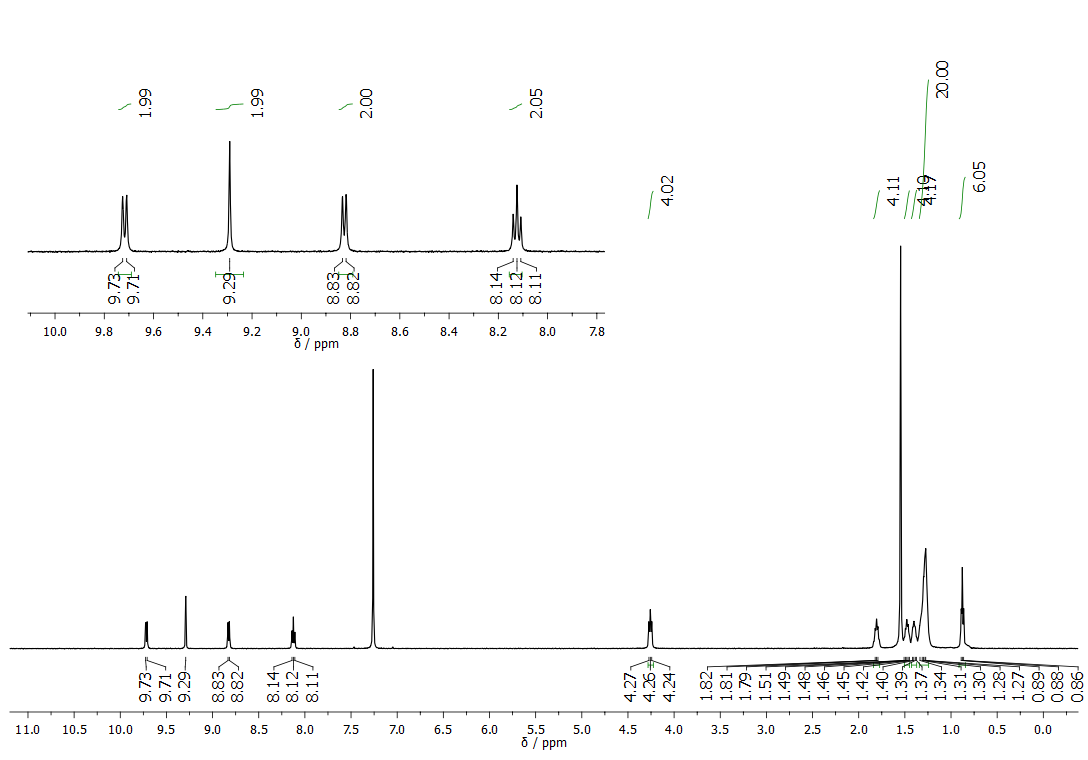


**Figure S28**. ^1^H NMR 500 MHz of **1c** in CDCl_3_


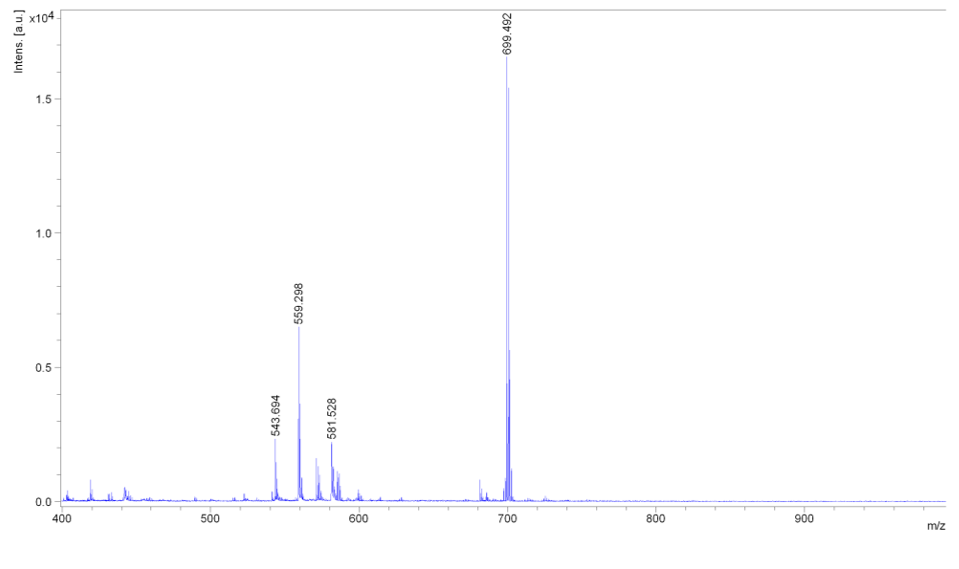


**Figure S29**. MALDI-LRMS of **1c**.

## **3.11 Characterization of 1d**


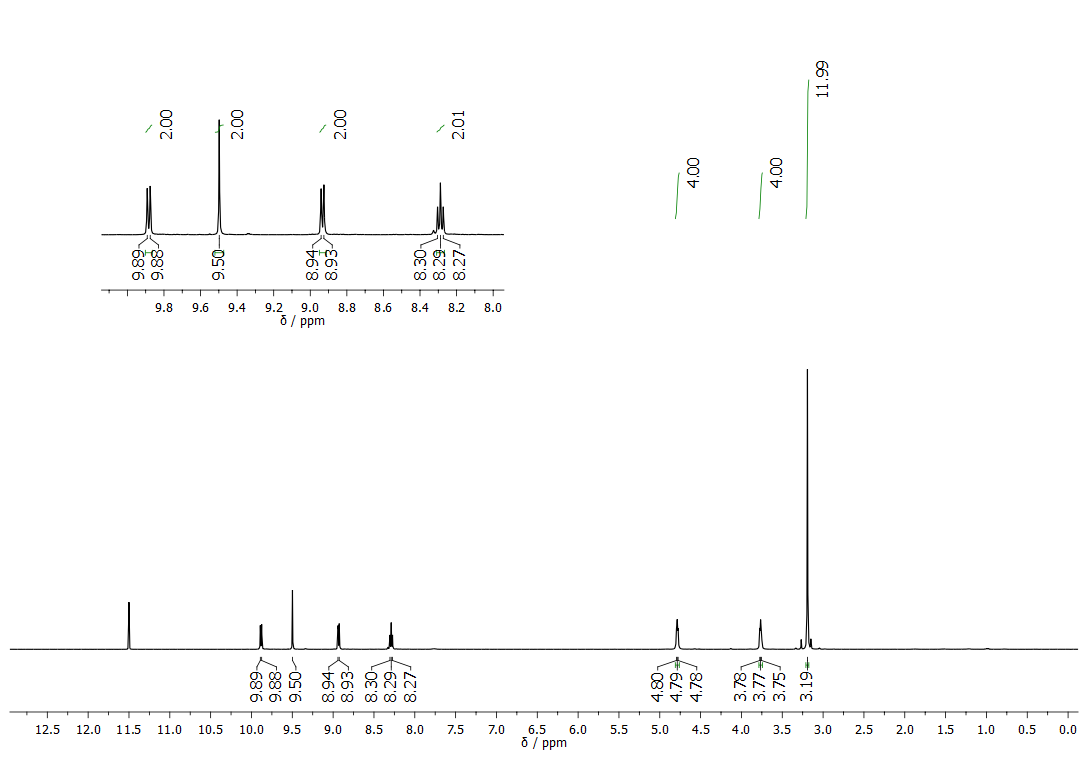


**Figure S30**. ^1^H NMR 500 MHz of **1d** in TFA-*d*1.


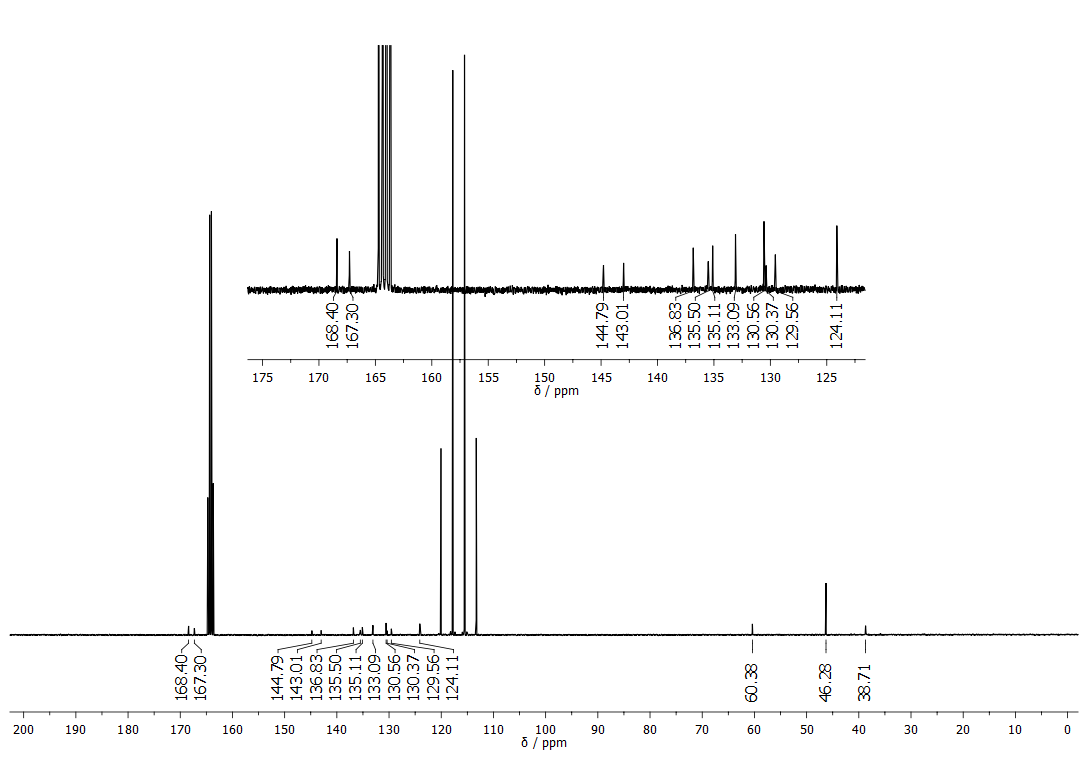


**Figure S31**. ^13^C NMR 126 MHz of **1d** in TFA-*d*1.


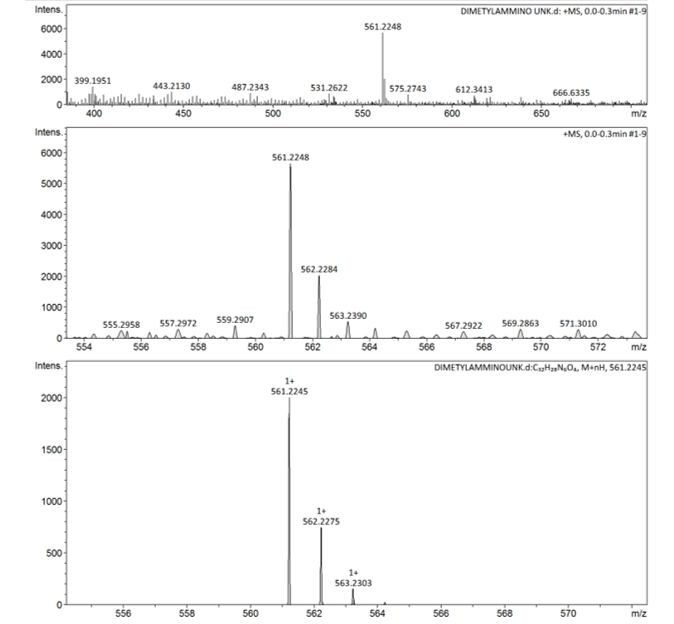


**Figure S32**. ESI-HRMS of **1d**, top) experimental spectra, middle) zoom and bottom) simulated spectra.

## **3.12 Characterization of 1e**


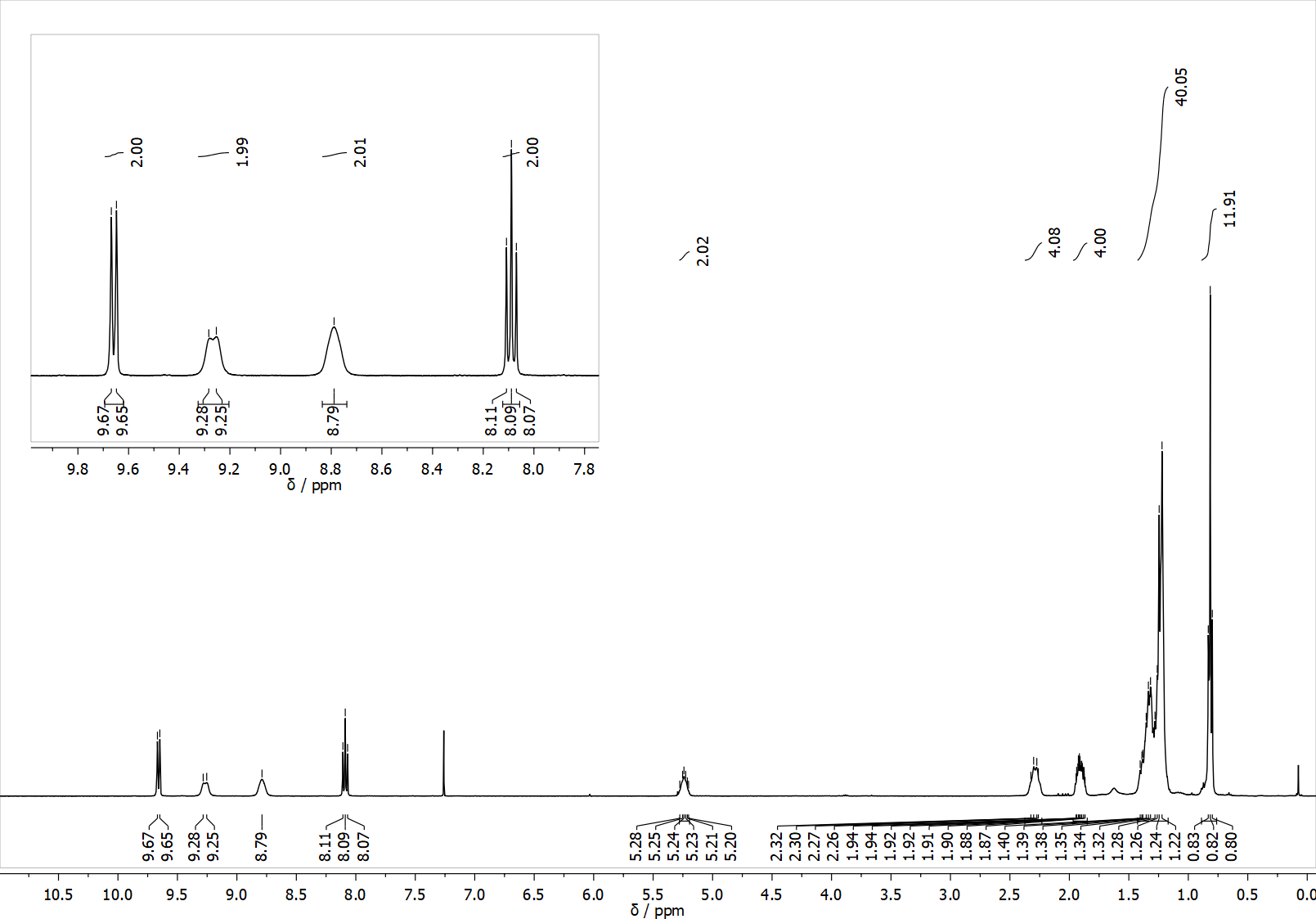


**Figure S33**. ^1^H NMR 400 MHz of **1e** in CDCl_3_.


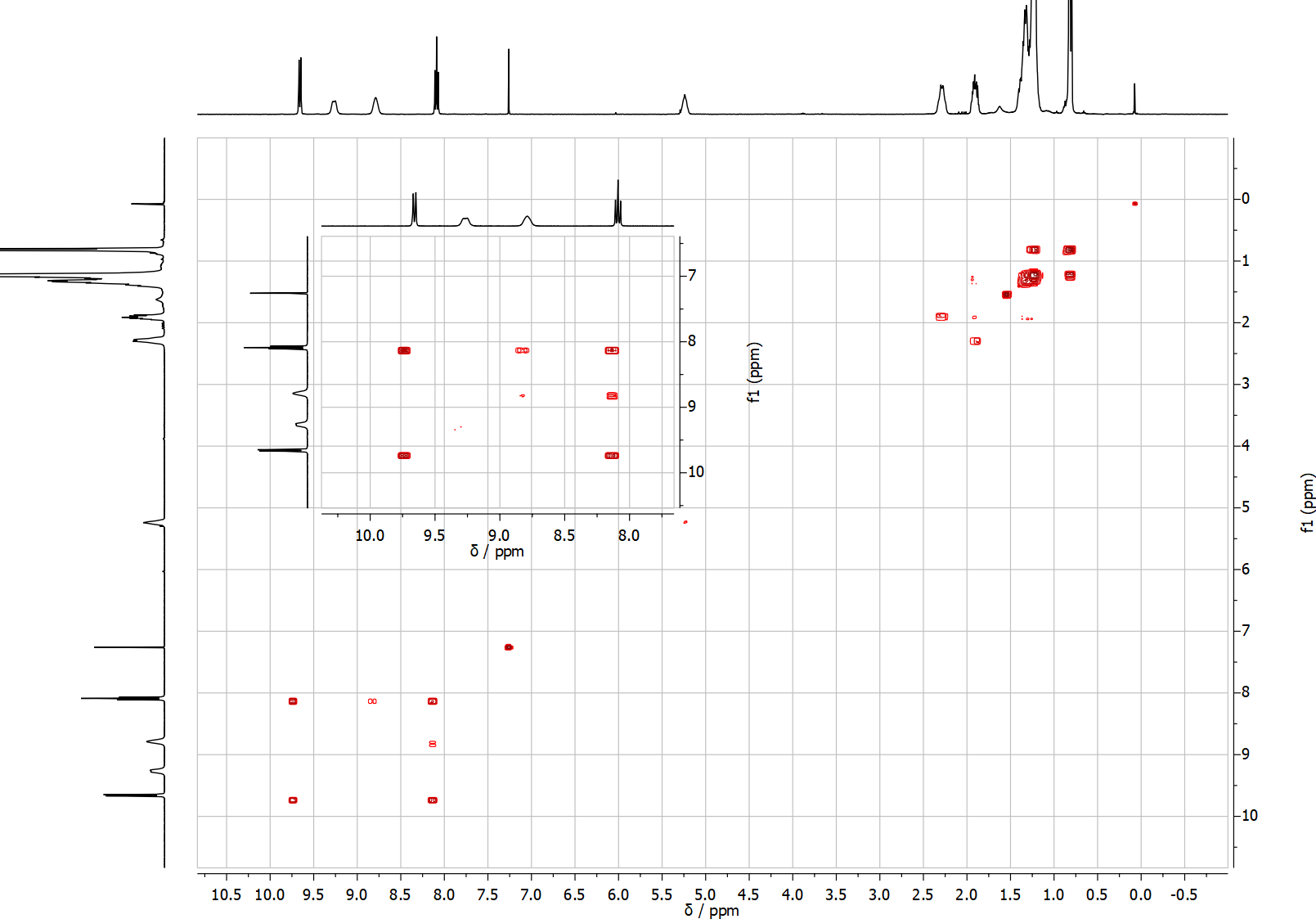


**Figure S34**. ^1^H-^1^H COSY NMR 400 MHz of **1e** in CDCl_3_.


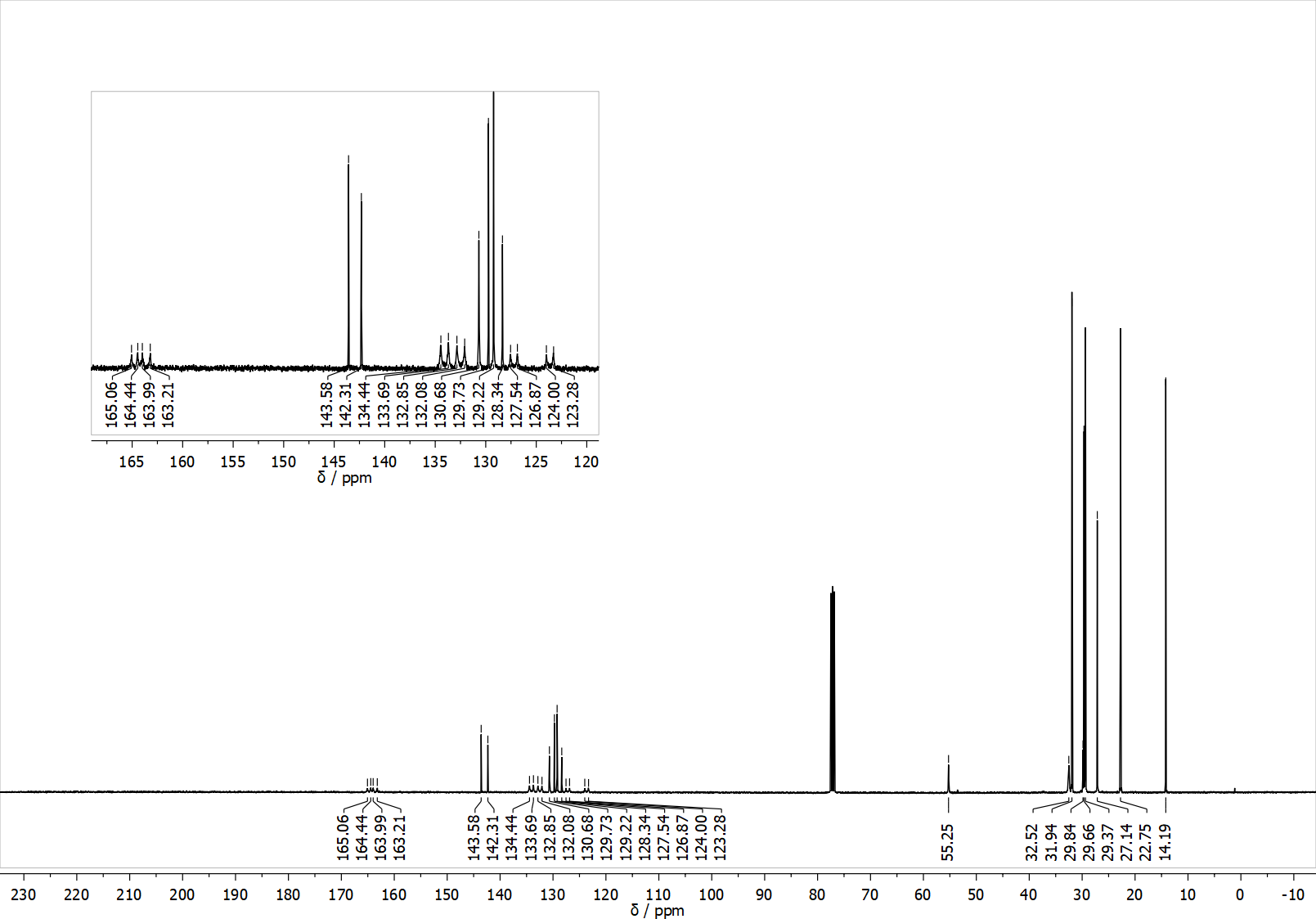


**Figure S35**. ^13^C NMR 101 MHz of **1e** in CDCl_3_.


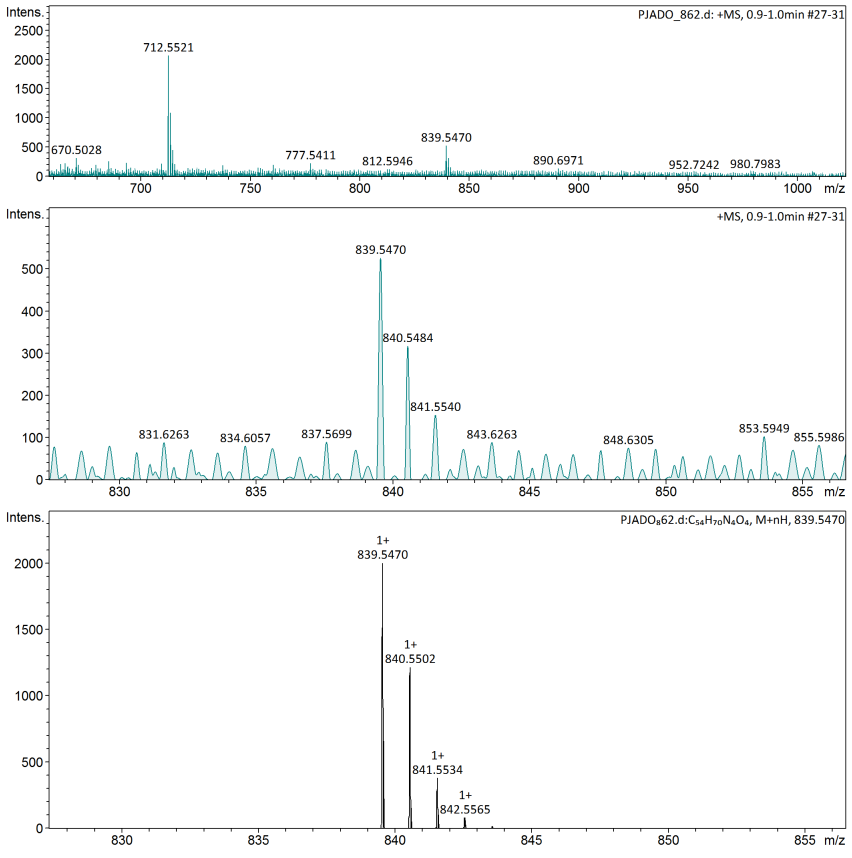


**Figure S36**. ESI-HRMS of **1e**, top) experimental spectra, middle) zoom and bottom) simulated spectra.

## **3.13 Characterization of 1f**


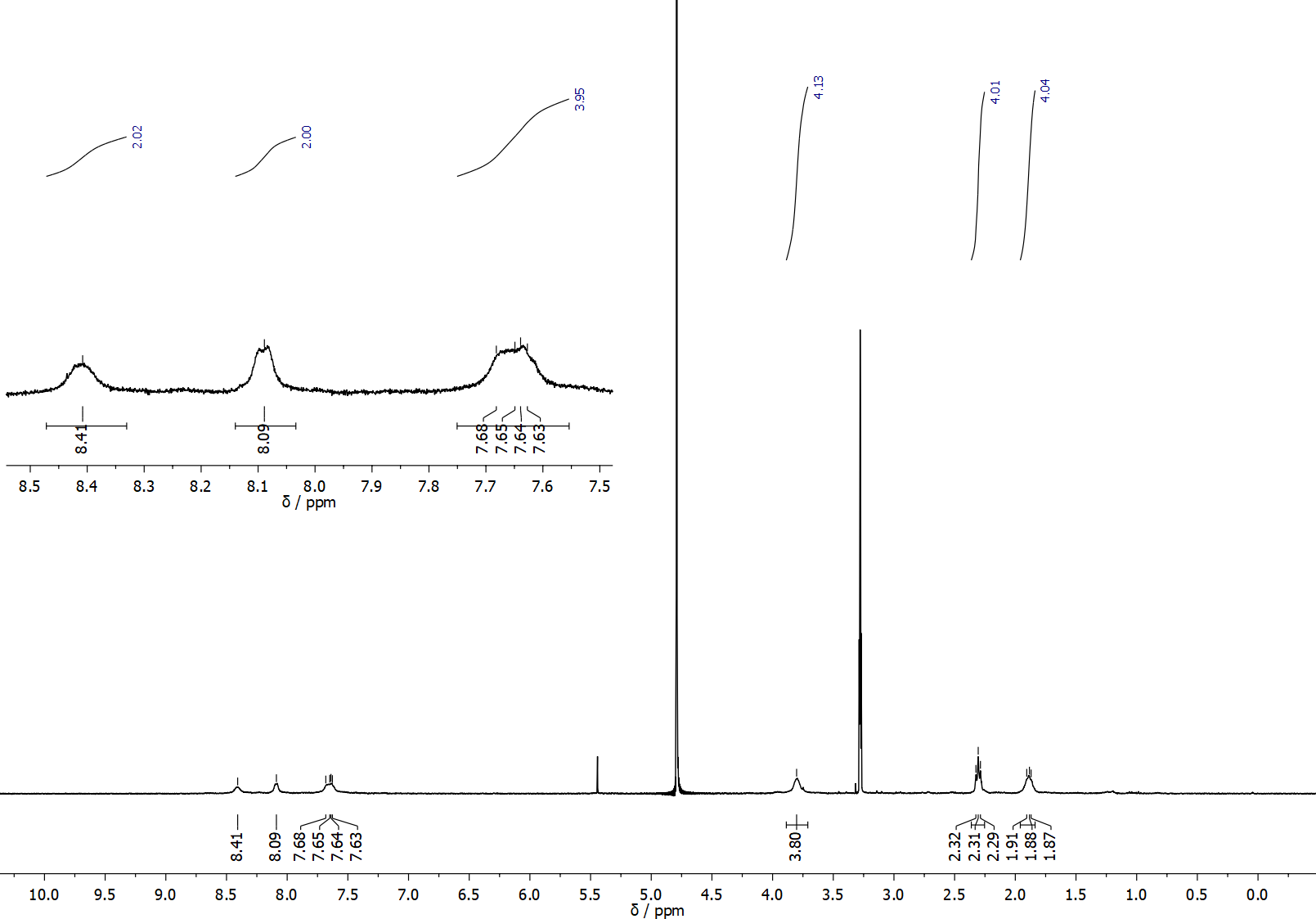


**Figure S37**. ^1^H NMR 400 MHz of **1f** in D_2_O/MeOD 1/1 Cs_2_CO_3_ 50 mM.


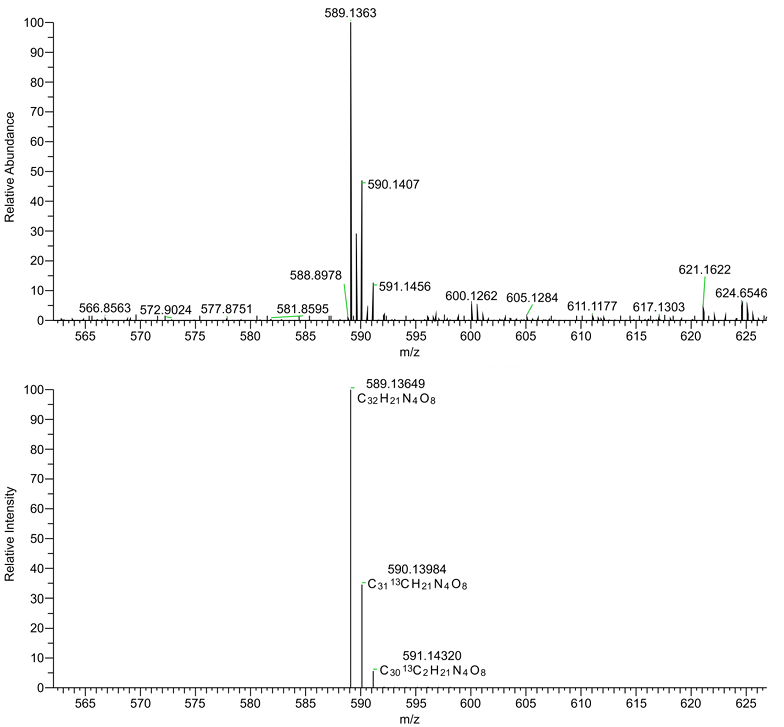


**Figure S38**. ESI-HRMS of **1f**, top) experimental spectra, and bottom) simulated spectra.

# **4 Reduced states NMR characterization**


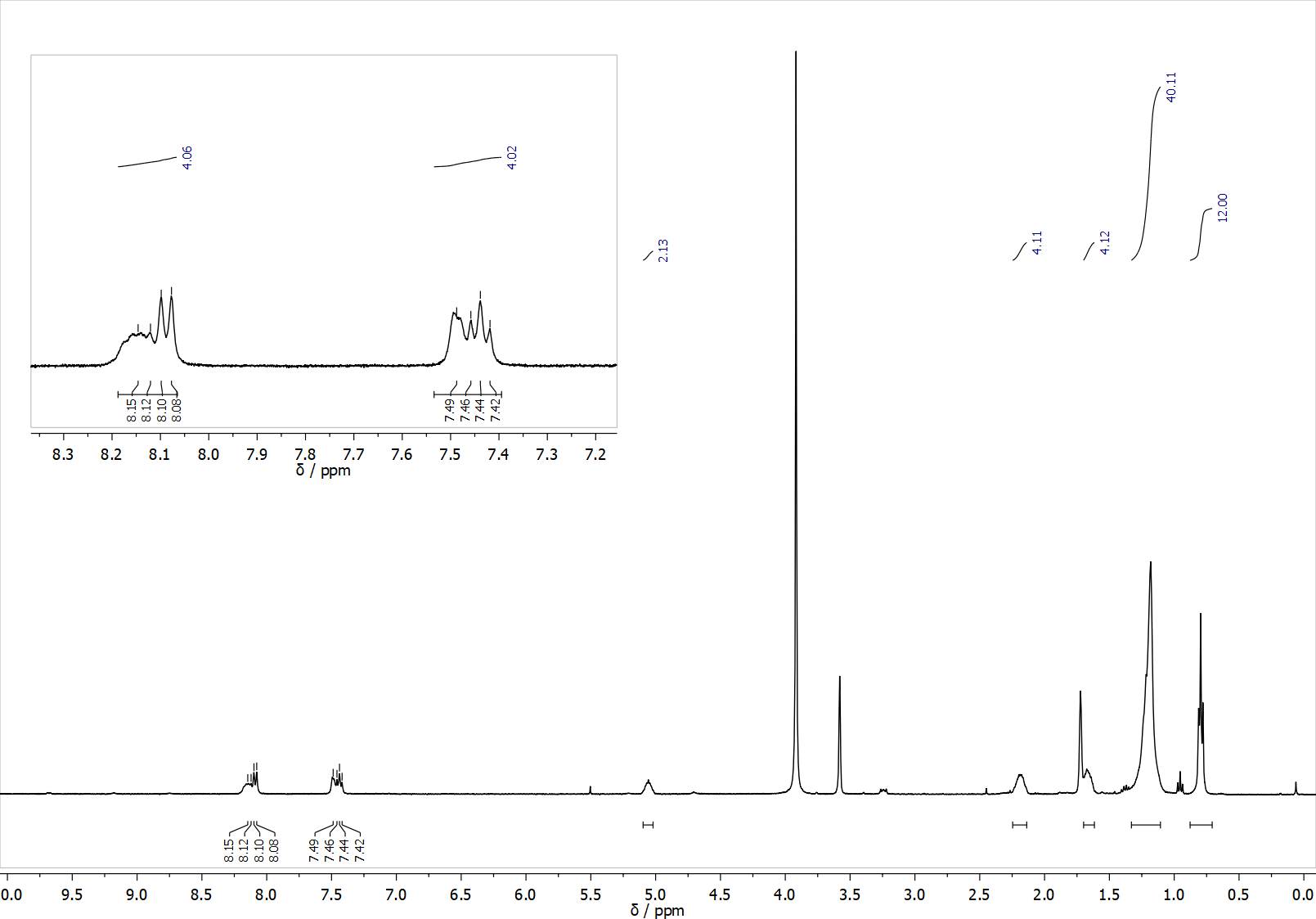


**Figure S39**. ^1^H-NMR 400 MHz of **1e** in degassed (Ar) THF-*d8*/D_2_O 3/1 containing an excess of Na_2_S_2_O_4_.


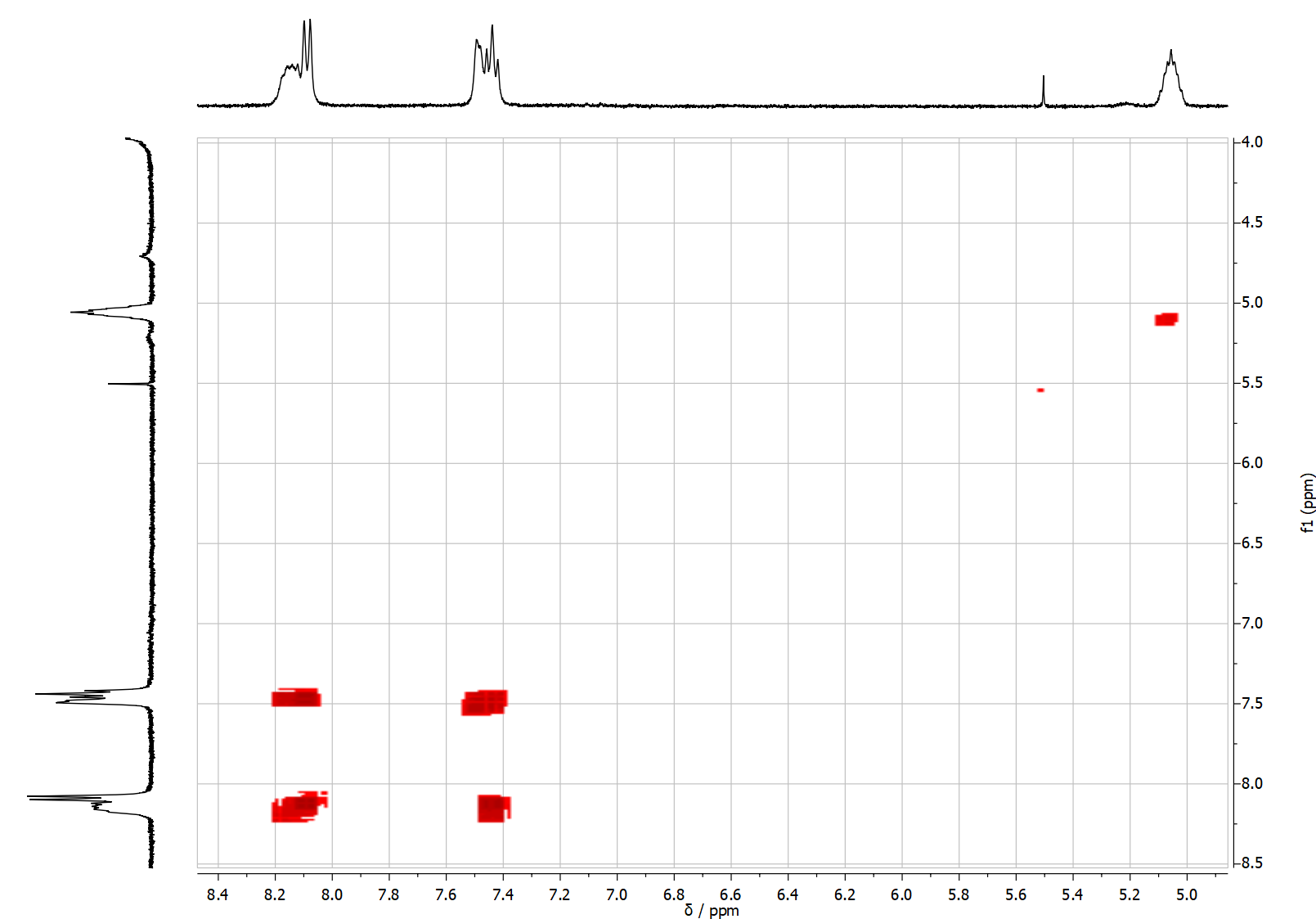


**Figure S40**. ^1^H-^1^H-COSY NMR 400 MHz of **1e** in degassed (Ar) THF-*d8*/D_2_O 3/1 containing an excess of Na_2_S_2_O_4_.


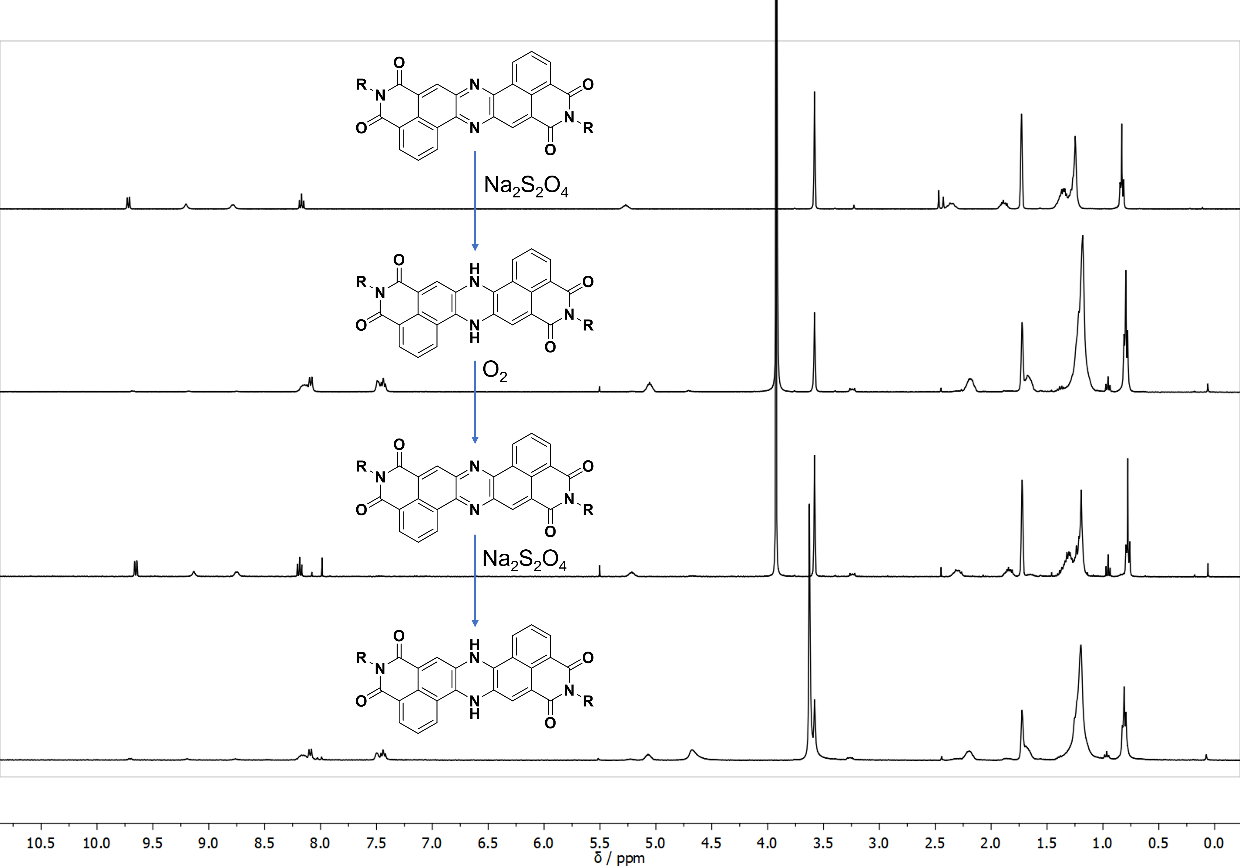


**Figure S41**. Reduction/Oxidation cycles in THF-*d*8/D_2_O mixture. From top to bottom: 1) **1e** in THF-*d*8; 2) Reduction performed in THF-*d*8 and D_2_O (3:1) using a large excess of Na_2_S_2_O_4_ as reductant; 3) Complete oxidation observed after 20h in the same sealed NMR tube; 4) Reduction performed on the same tube by adding again an excess of Na_2_S_2_O_4_. See above for more details.


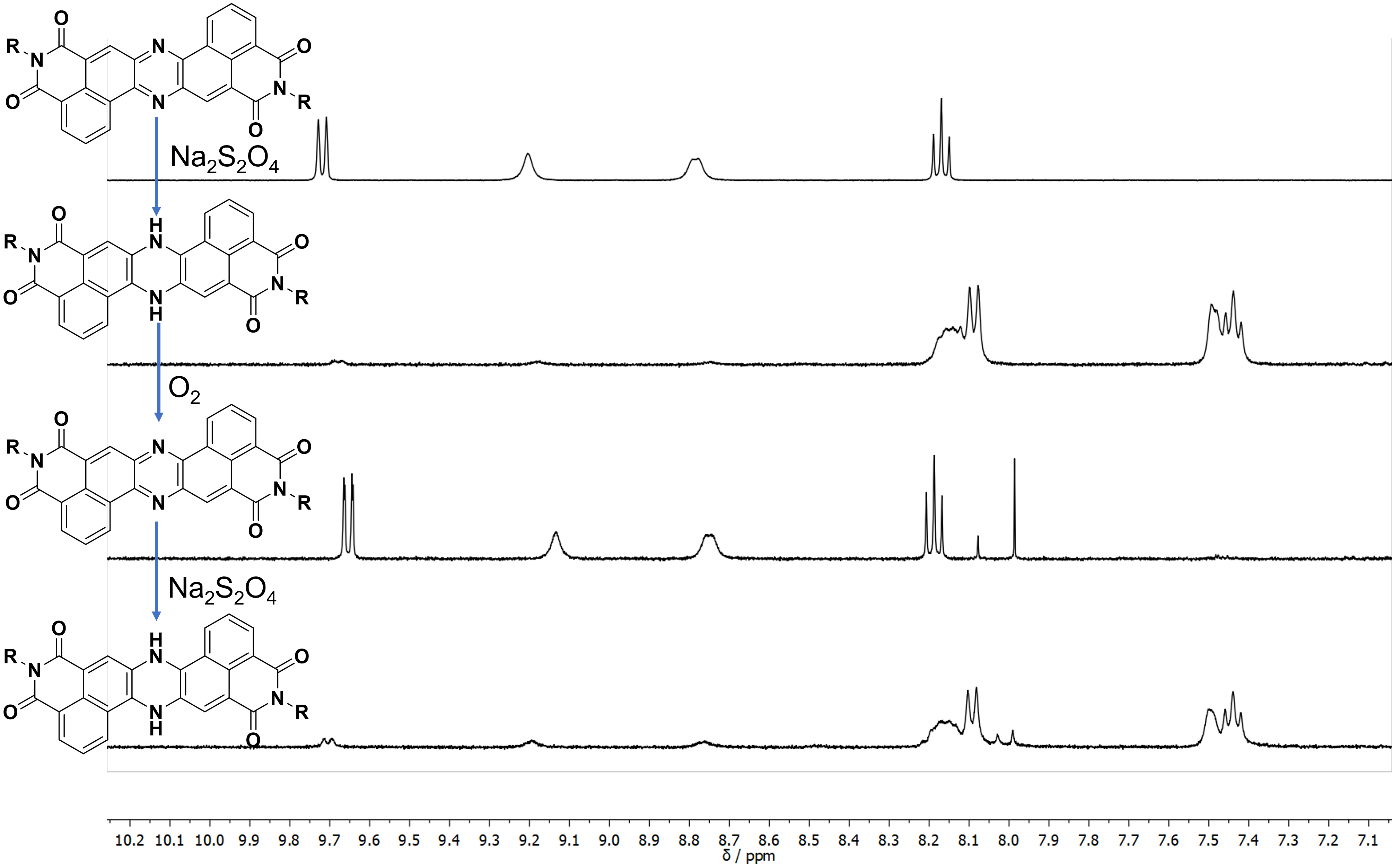


**Figure S42**. Zoom on the aromatic region for the Reduction/Oxidation cycles in THF-*d*8/D_2_O mixture.


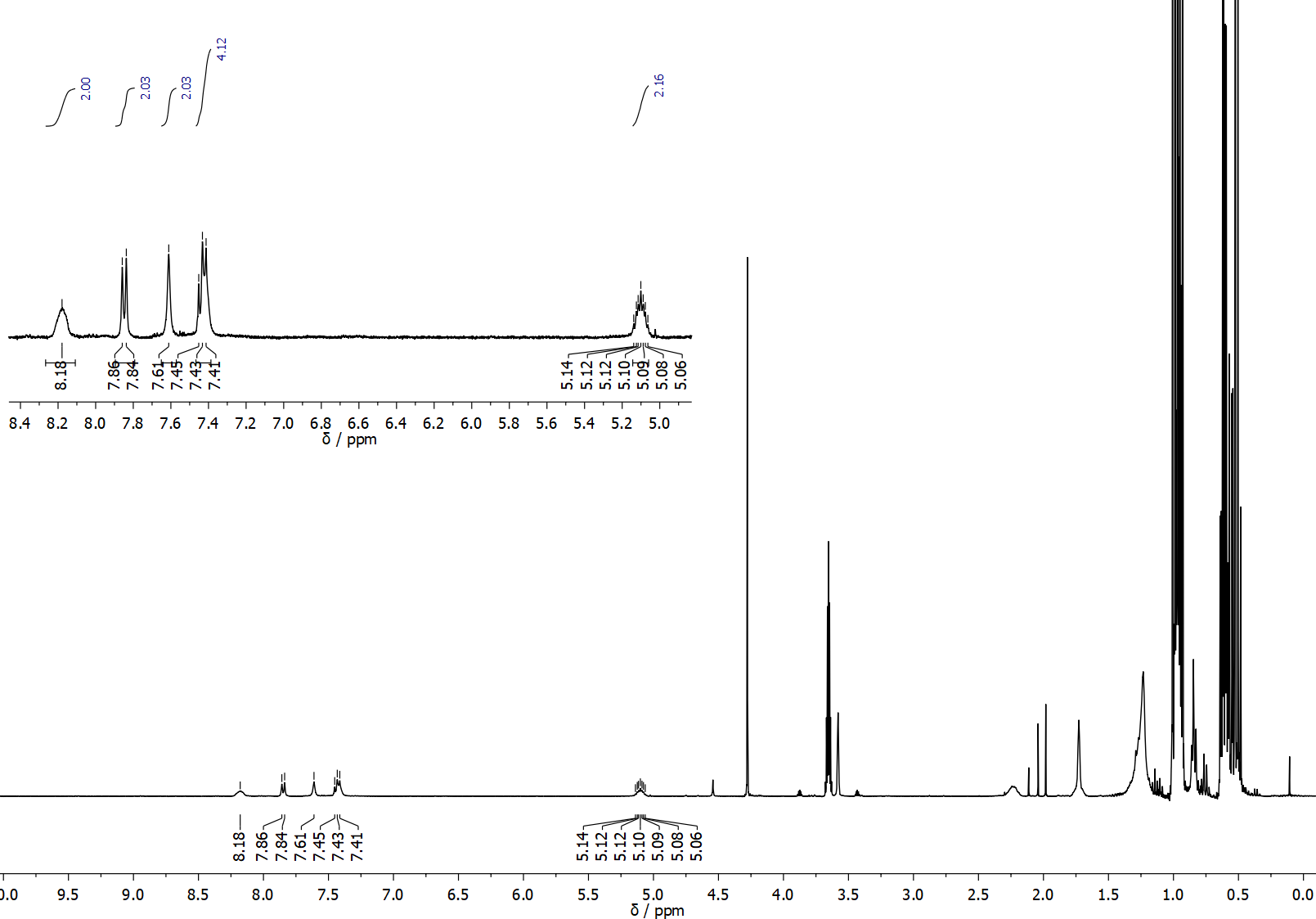


**Figure S43**. ^1^H-NMR 400 MHz of **1e** in degassed (Ar) THF-*d8* containing an excess of Et_3_SiH with catalytic Pd(OAc)_2_.


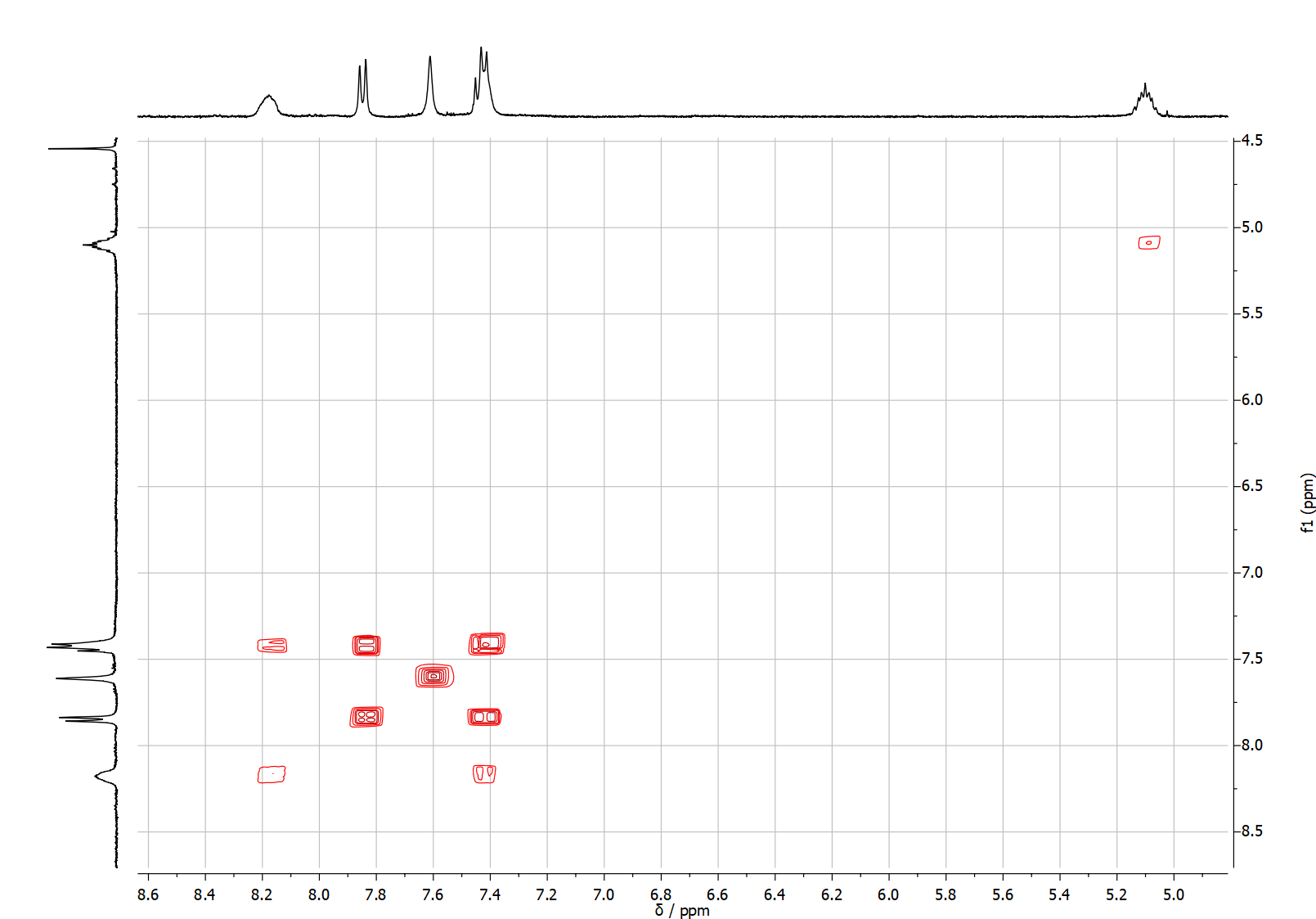


**Figure S44**. ^1^H-^1^H-COSY NMR 400 MHz of **1e** in degassed (Ar) THF-*d8* containing an excess of Et_3_SiH with catalytic Pd(OAc)_2_.


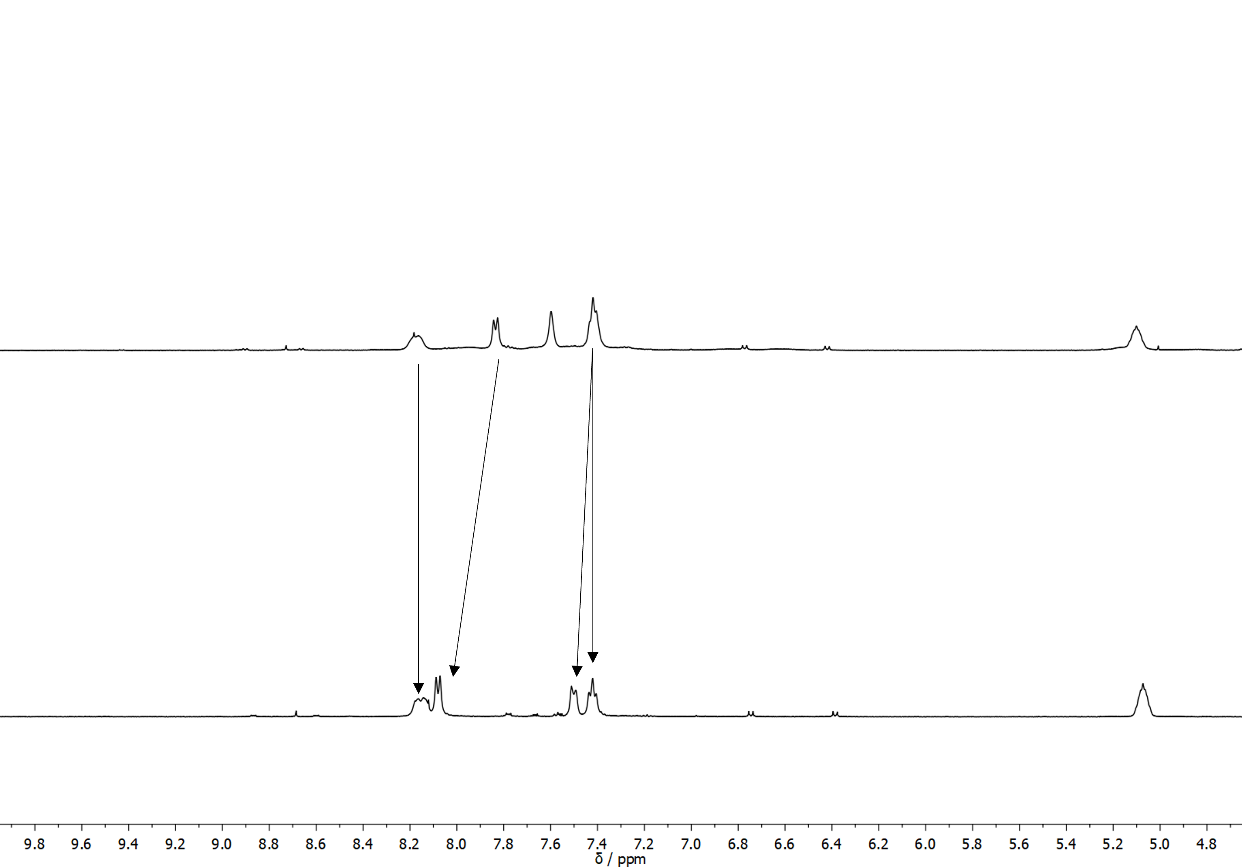


**Figure S45**. Top) ^1^H NMR 500 MHz of the aromatic region of **1e** in degassed (Ar) THF-*d8* containing an excess of Et_3_SiH with catalytic Pd(OAc)_2_. Bottom) following addition of D_2_O, NH signal disappears.


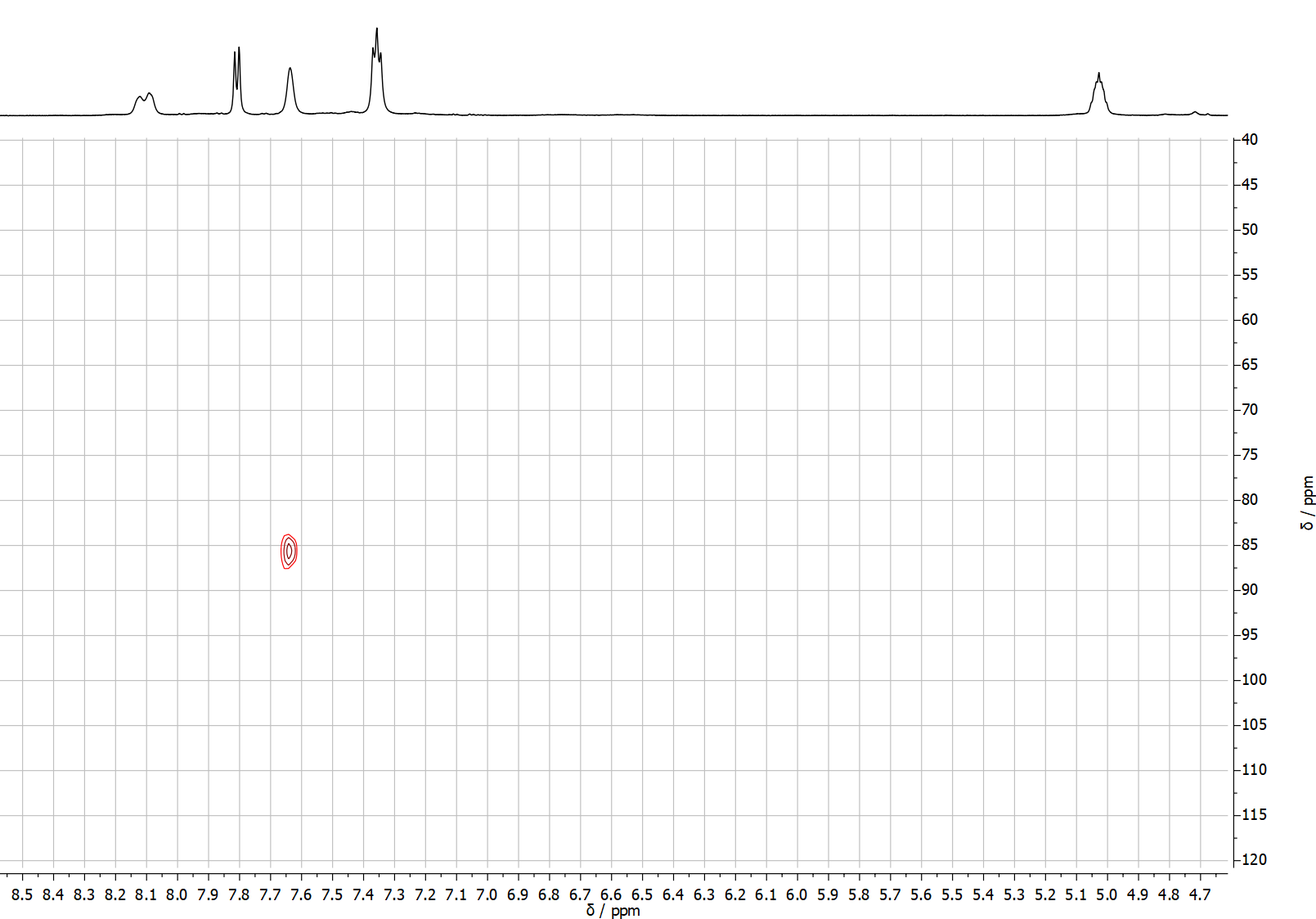


**Figure S46**. ^1^H-^15^N-HSQC NMR 600 MHz of **1e** in degassed (Ar) THF-*d8* containing an excess of Et_3_SiH with catalytic Pd(OAc)_2_.


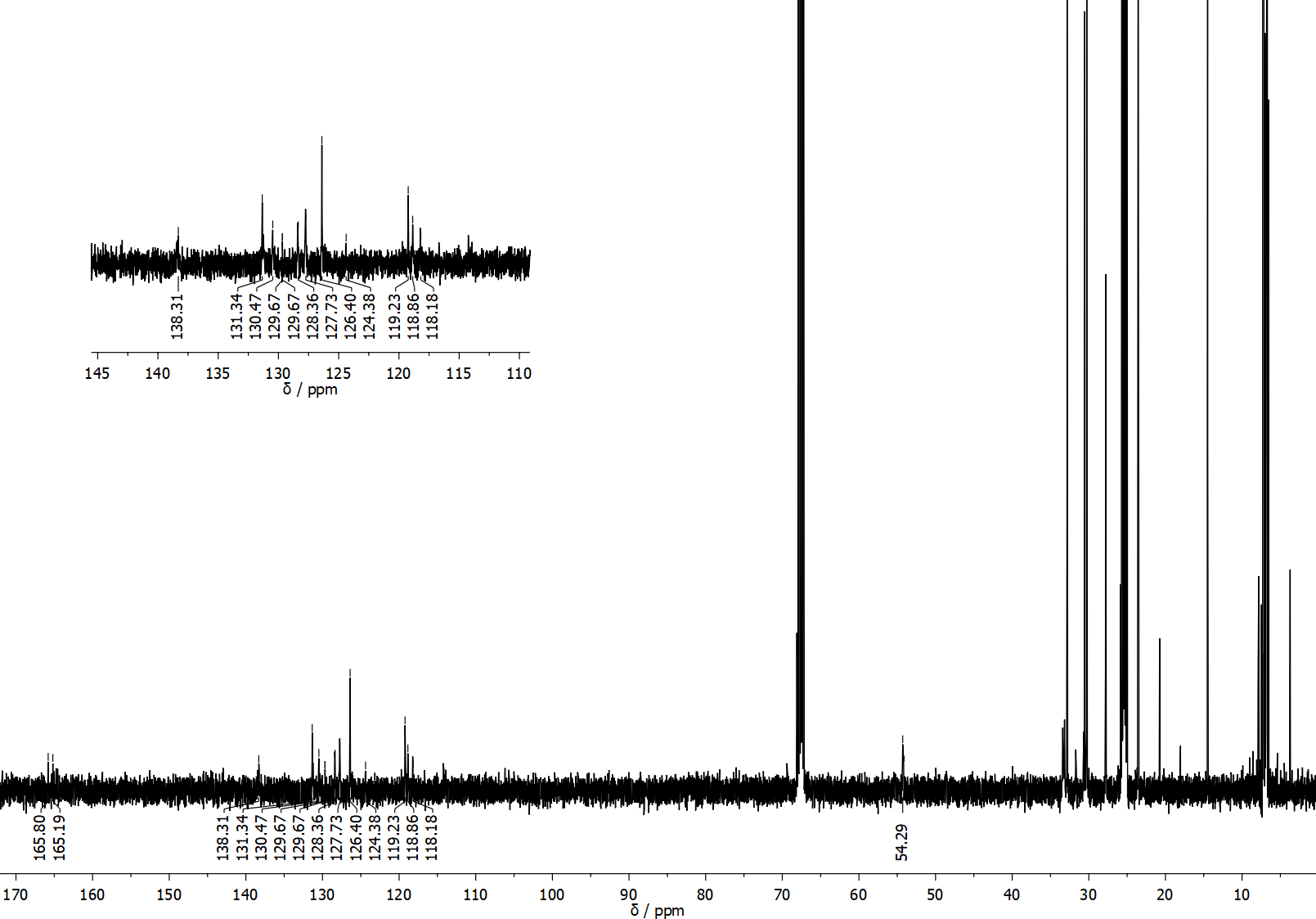


**Figure S47**. ^13^C NMR 126 MHz of **1e** in degassed (Ar) THF-*d8* containing an excess of Et_3_SiH with catalytic Pd(OAc)_2_.


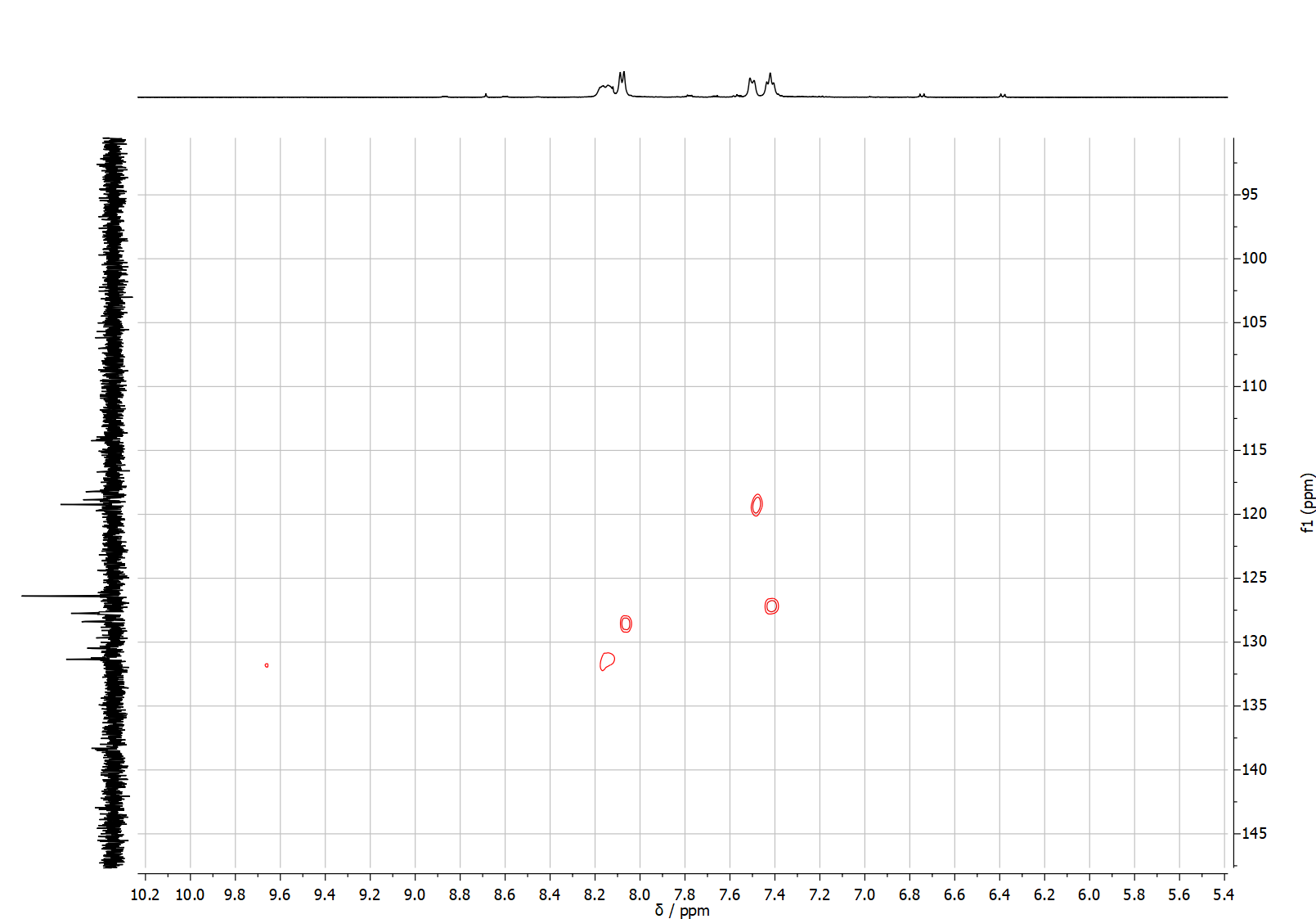


**Figure S48**. ^1^H-^13^C HSQC NMR of the aromatic part of **1e** in degassed (Ar) THF-*d8* containing an excess of Et_3_SiH with catalytic Pd(OAc)_2_ after addition of D_2_O.


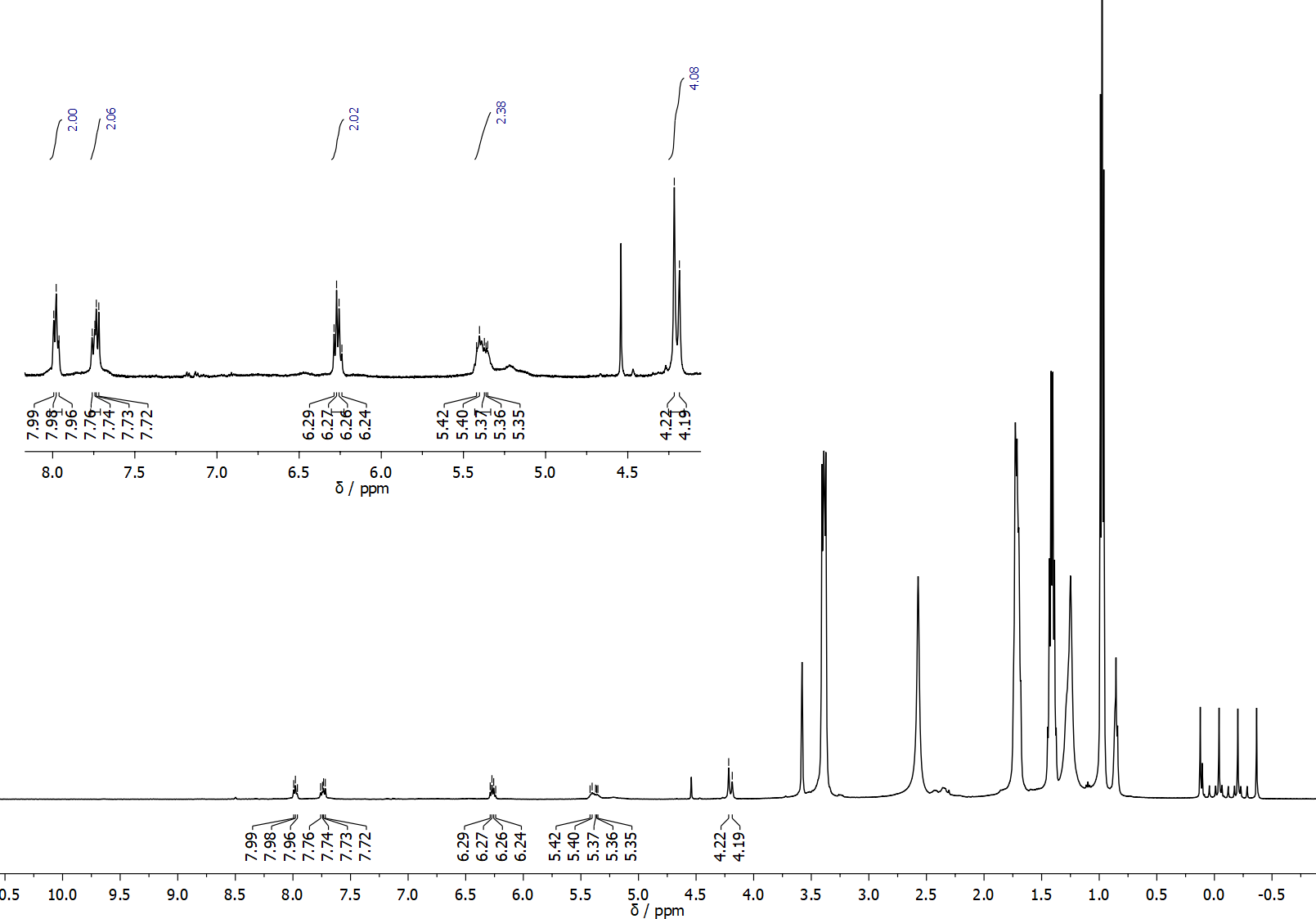


**Figure S49**. ^1^H-NMR 500 MHz of **1e** in degassed (Ar) THF-*d8* containing 3 equivalents of TBABH_4_.


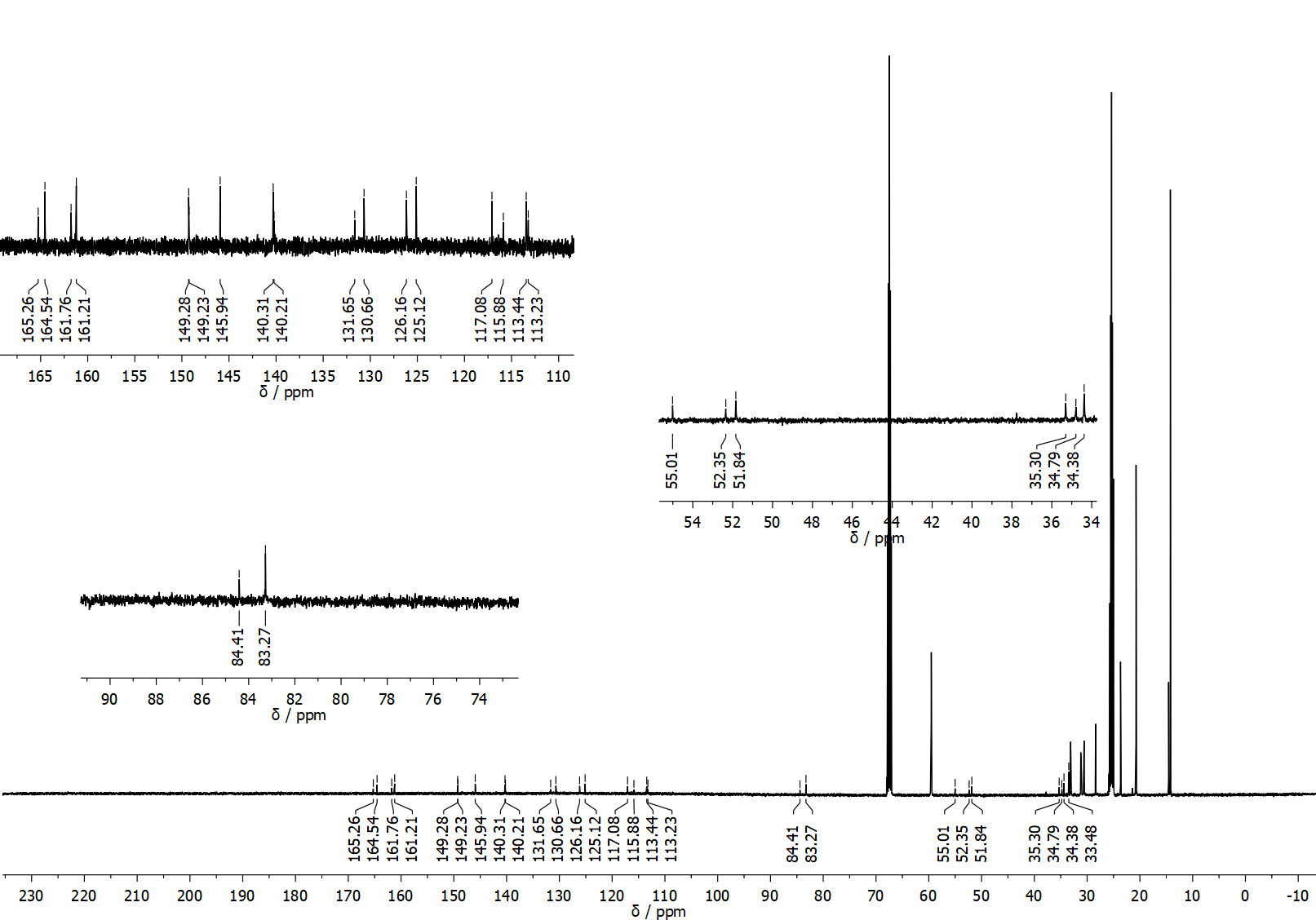


**Figure S50**. ^13^C-NMR 126 MHz of **1e** in degassed (Ar) THF-*d8* containing 3 equivalents of TBABH_4_.


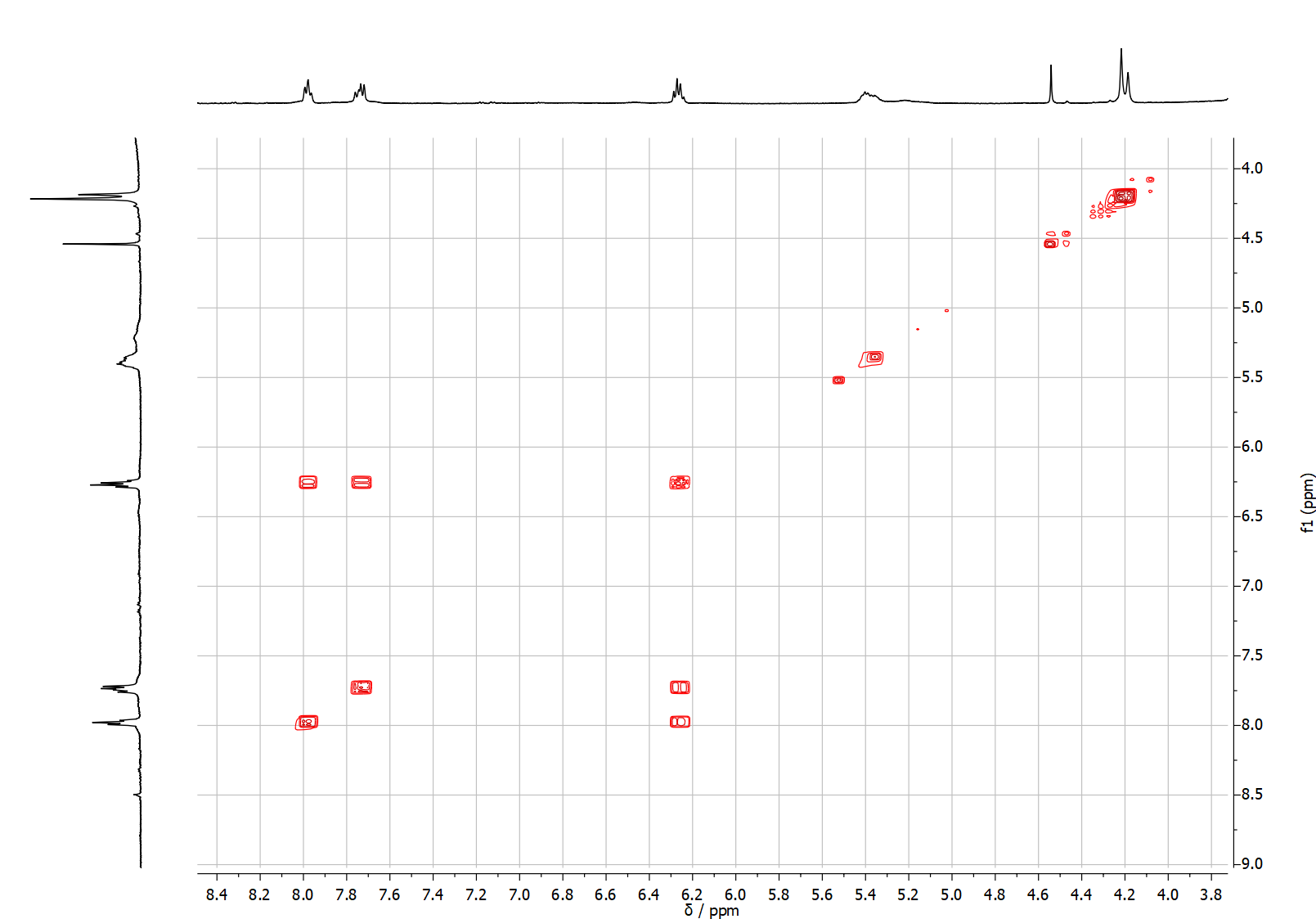


**Figure S51**. ^1^H-^1^H-COSY NMR 500 MHz of **1e** in degassed (Ar) THF-*d8* containing 3 equivalents of TBABH_4_.


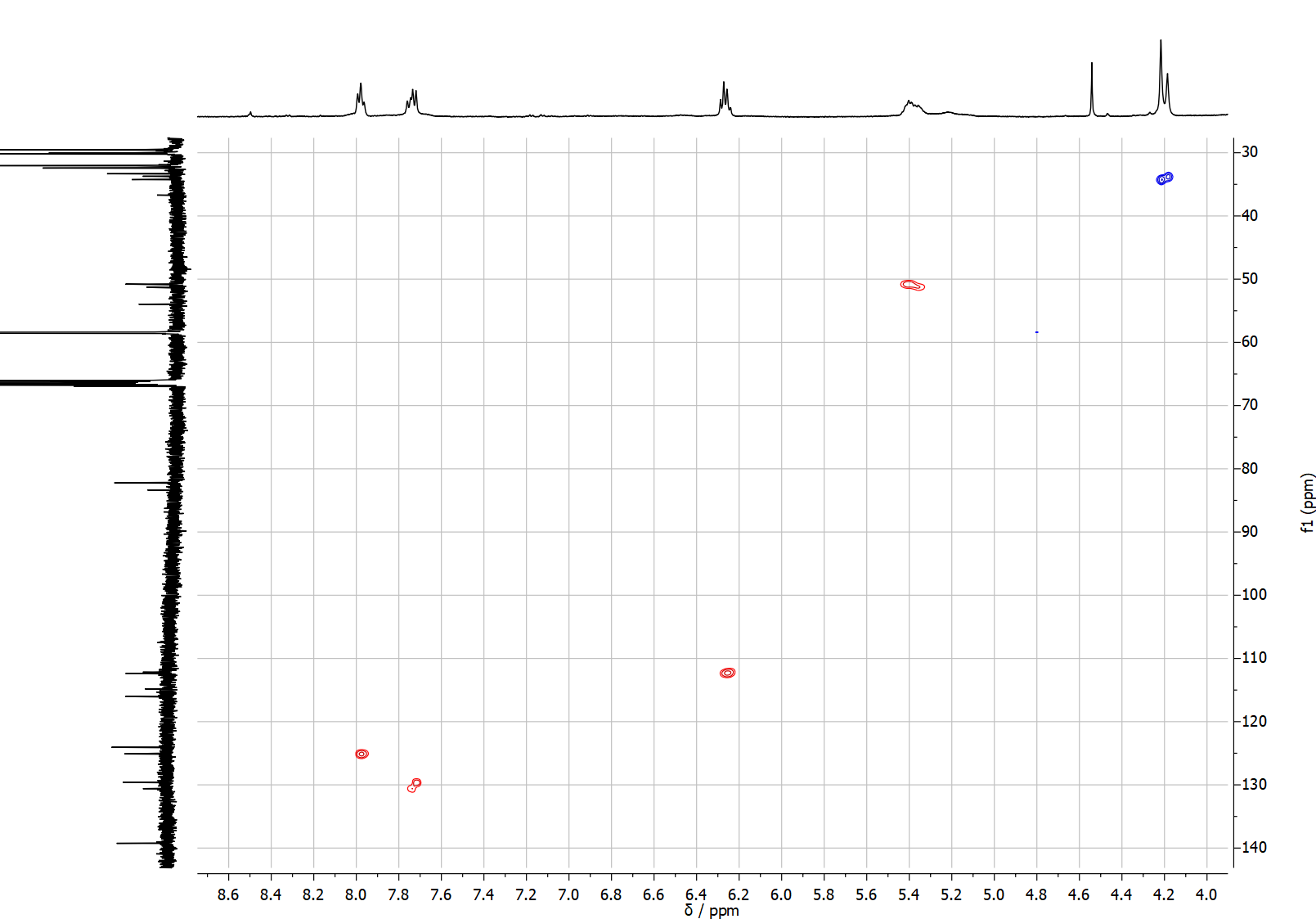


**Figure S52**. ^1^H-^13^C-HSQC NMR of **1e** in degassed (Ar) THF-*d8* containing 3 equivalents of TBABH_4_.


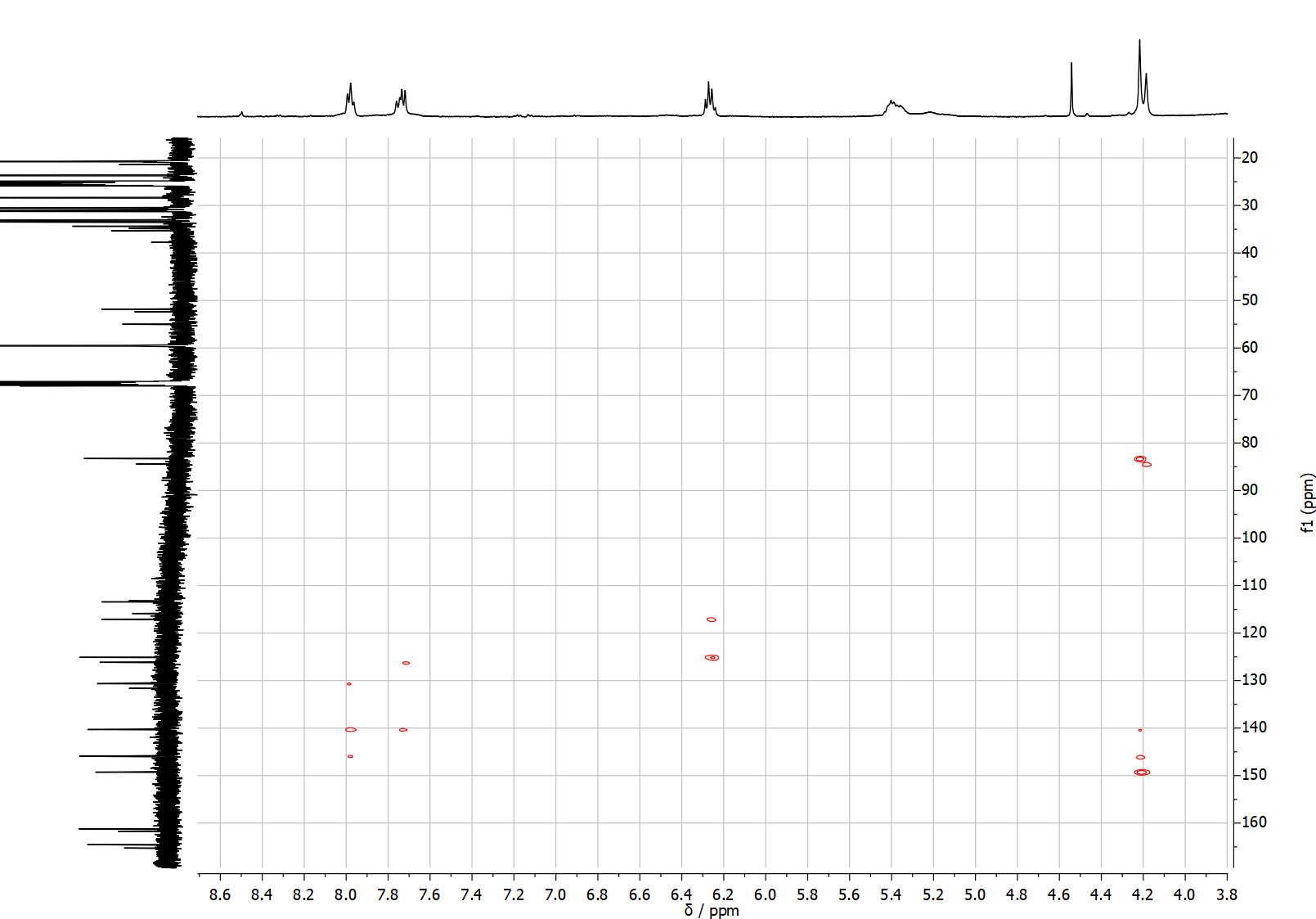


**Figure S53**. ^1^H-^13^C-HMBC NMR of **1e** in degassed (Ar) THF-*d8* containing 3 equivalents of TBABH_4_.

# **5 Photophysical characterization**


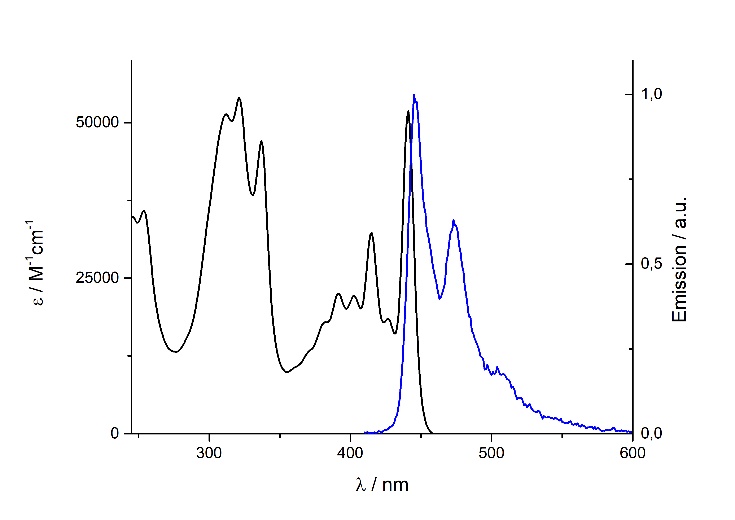


**Figure S54.** Absorption (black) λ_abs(max)_= 441 nm and fluorescence spectrum (blue) λ_em(max)_= 447 nm (exc = 400 nm) of **1c** in air equilibrated CHCl_3_ at room temperature.


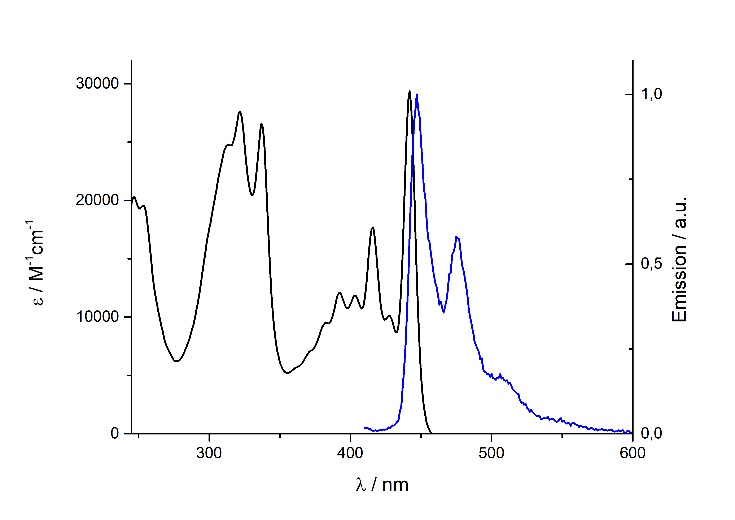


**Figure S55.** Absorption (black) λ_abs(max)_= 442 nm, and fluorescence spectrum (blue), λ_em(max)_= 446 nm (exc = 400 nm) of **1a** in air equilibrated CHCl_3_ at room temperature.

**Figure S56.** Absorption (black) λ_abs(max)_= 442 nm and fluorescence spectrum (blue) λ_em(max)_= 446 nm (exc = 400 nm) of **1e** in air equilibrated THF at room temperature.


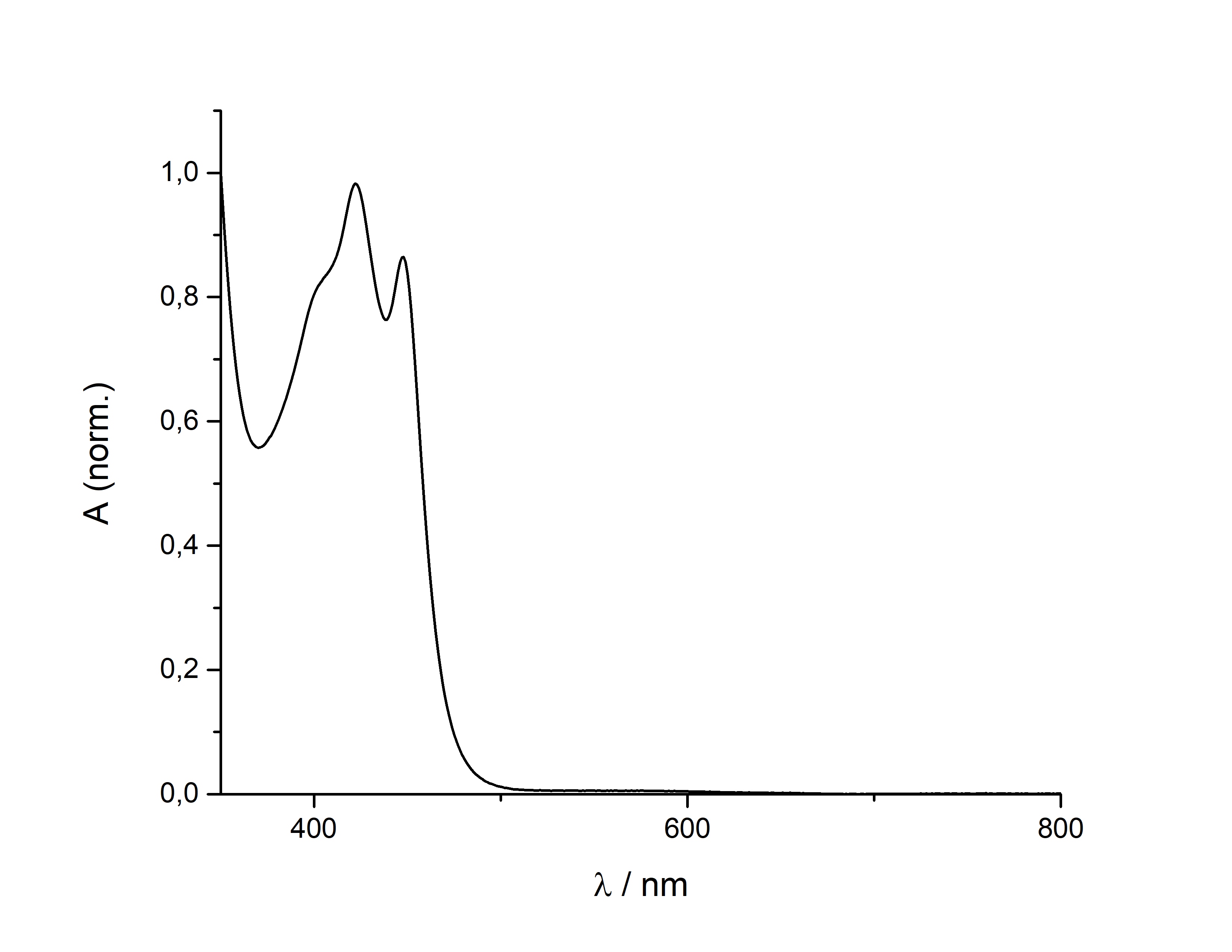


**Figure S57.** Absorption (black) λ_abs(max)_= 422 nm of **1f** in air equilibrated H_2_O containing 50 mM Cs_2_CO_3_ at room temperature.

**Figure S58.** Absorption (black) λ_abs(max)_= 600 nm, fluorescence (blue) λ_em(max)_= 636 nm (exc = 600 nm), and excitation (red) λ_ex(max)_= 570 nm (emm = 636 nm), spectrum of **1e^2H-^** in THF containing 10 mM TBABH_4_ under N_2_ at room temperature. The excitation spectrum confirms that the observed emission originates from the same electronic transition and excluding contributions from residual neutral species or emissive impurities.

**Figure S59.** Fluorescence emission-excitation 3D map of **1e^2H-^** (100 μM) in THF containing 10 mM TBABH_4_ under N_2_ at room temperature. The excitation–emission 3D map shows a single emissive manifold over the investigated spectral range, supporting the presence of one dominant emissive species and the absence of multiple emitting states.

**Figure S60.** Time-resolved emission decay (excitation at 402 nm, analysis at 448 nm) of **1e** in THF solution measured by TC-SPC.

**Figure S61.** Time-resolved emission decay (excitation at 635 nm, analysis at 650 nm) of **1e^2H^**^-^ in THF solution measured by TC-SPC. Time-correlated single photon counting (TCSPC) measurements reveal a nanosecond-scale excited-state lifetime (τ = 3.8 ns), consistent with fluorescence from a singlet excited state. The monoexponential decay profile further supports assignment of the emission to a single singlet excited state, with no evidence of long-lived triplet or charge-separated species.

# **6 Electrochemical characterizations**


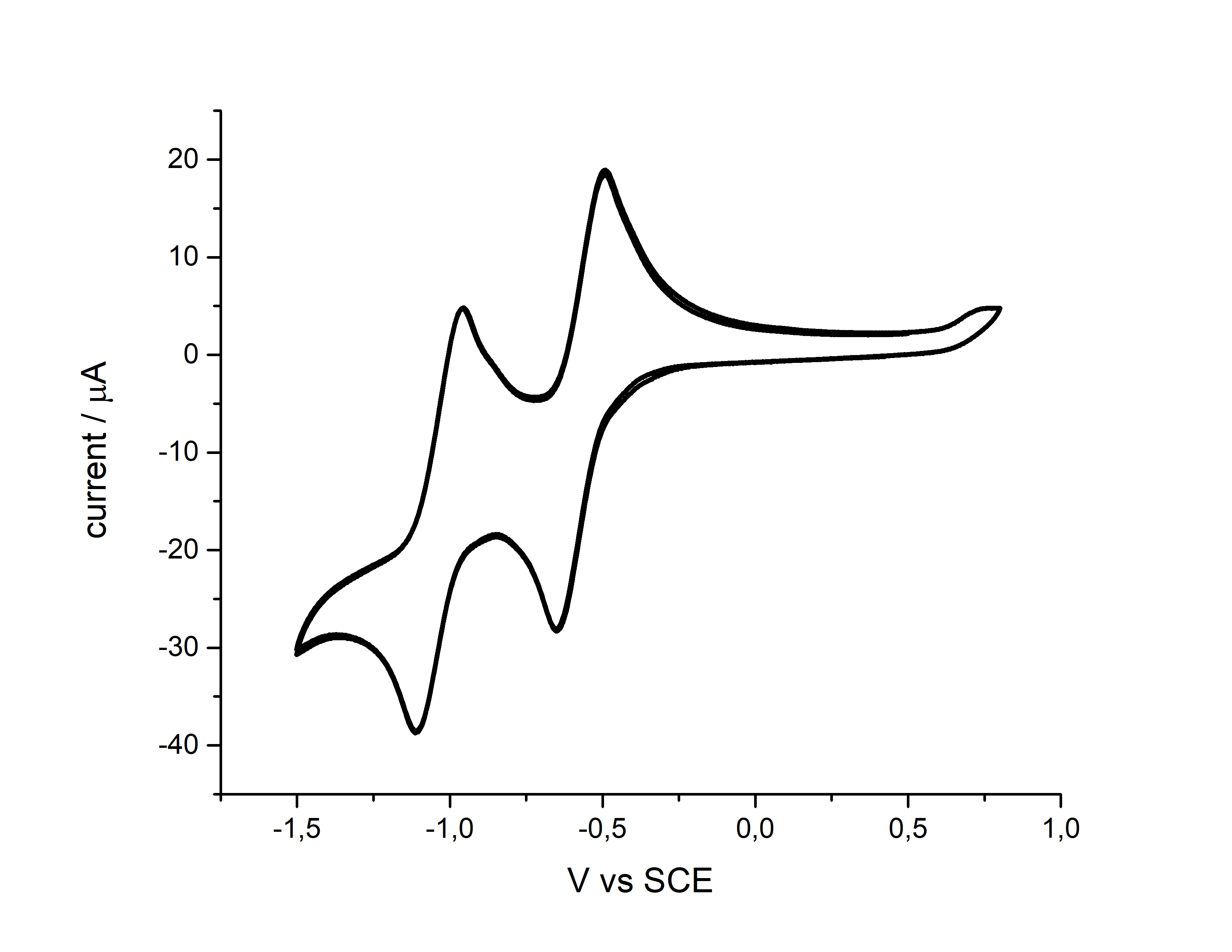


**Figure S62.** Cyclic voltammograms of **1e** in THF (ca. 1 mM). Scan rates: 0.1 V s^-1^. TBAPF_6_ (0.1 M) is used as a supporting electrolyte.


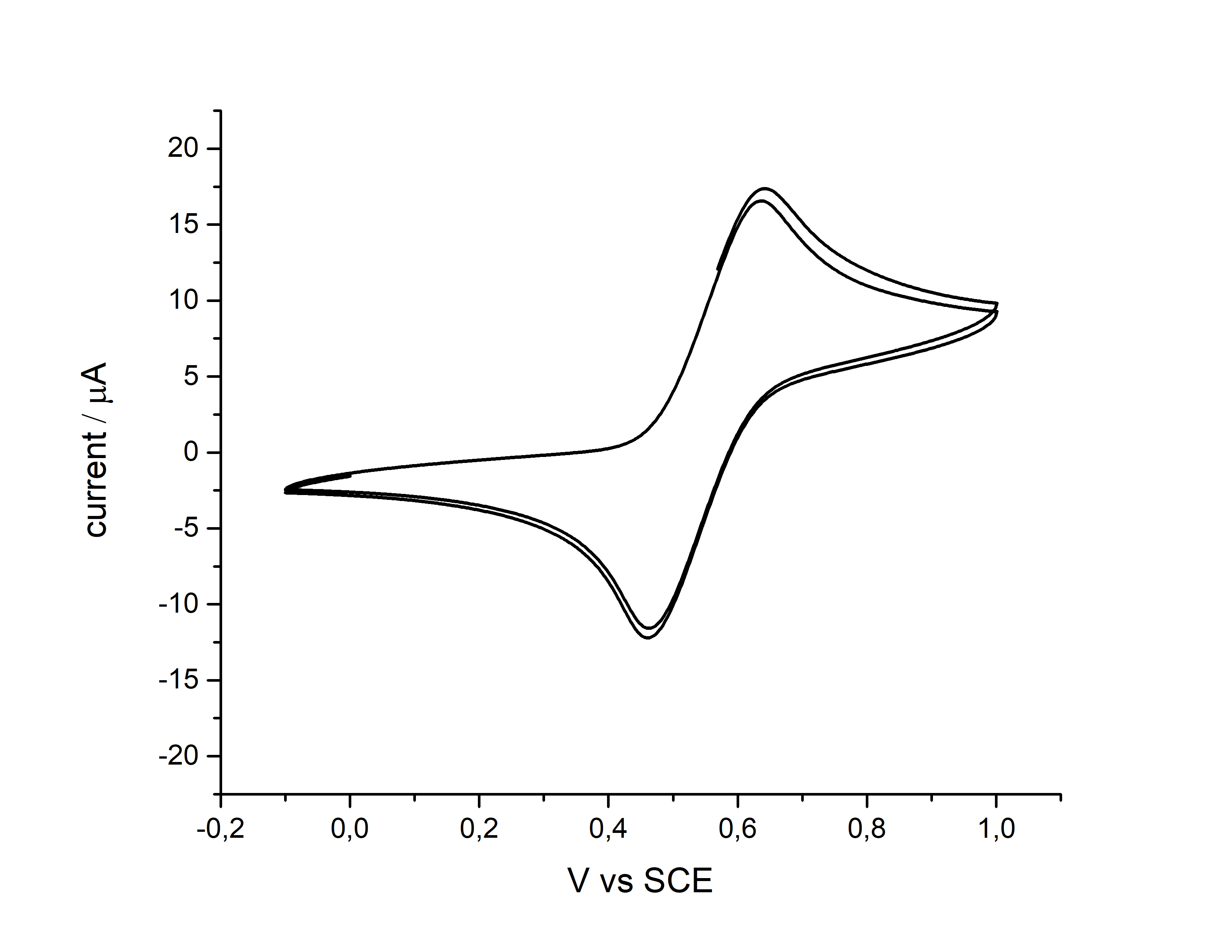


**Figure S63.** Cyclic voltammograms of **Fc** in THF (ca. 1 mM). Scan rates: 0.1 V s^-1^. TBAPF_6_ (0.1 M) is used as a supporting electrolyte E_1/2_= +0.55 V.

**Figure S64.** Cyclic voltammograms of **1e^2H-^** in THF (ca. 1 mM) with 20 equiv TBABH_4_. Scan rates: 0.1 V s^-1^. TBAPF_6_ (0.1 M) is used as a supporting electrolyte.

**Figure S65.** Cyclic voltammograms of **1e^2H-^** in THF (ca. 1 mM) with 20 equiv TBABH_4_. Scan rates: 0.1 V s^-1^. TBAPF_6_ (0.1 M) is used as a supporting electrolyte.

**Table S1.** Summary of excited- and ground-state photoredox properties in THF

|  | λmax (abs) (nm) | λmax (emm) (nm) | E_00_ (eV) | τ (ns) | *E*(3/3^.−^) vs SCE | *E*(3^.+^/3) |
| --- | --- | --- | --- | --- | --- | --- |
| **1e** | 442 | 446 | 2.8 | 4.4 | -0.51 | - |
| **1e^2H-^** | 600 | 636 | 2.0 | 3.8 | -1.18 | 0.90 (peak) |

# **7 Crystallographic characterizations**

Data collections for **1a** and **1b** were performed at the XRD2 beamline of the Elettra Synchrotron, Trieste (Italy).^[7]^ The crystals were dipped in NHV oil (Jena Bioscience, Jena, Germany) and mounted on the goniometer head with kapton loops (MiTeGen, Ithaca, USA). Complete datasets were collected at 100 K or 298 K (nitrogen stream supplied through an Oxford Cryostream 700) through the rotating crystal method. Data were acquired using monochromatic wavelength of 0.620 Å on Pilatus 6M hybrid-pixel area detector (DECTRIS Ltd., Baden-Daettwil, Switzerland). The diffraction data were indexed, integrated and scaled using XDS.^[8]^ Two different datasets, collected from random orientations of the same crystal, have been merged to obtain complete sets of data, using CCP4-Aimless code.^[9,10]^ The structures were solved by the dual space algorithm implemented in the SHELXT code.^[11]^ Fourier analysis and refinement were performed by the full-matrix least-squares methods based on F^2^ implemented in SHELXL (Version 2018/3)^[12]^. The Coot program was used for modeling.^[13]^ Anisotropic thermal motion refinement have been used for all atoms with occupancies greater than 50%. Geometry and thermal motion parameters restrains (SIMU, DFIX, SADI and FLAT) have been used to refine disordered solvent molecules. No constrains have been imposed on atoms of diimide polyaromatic backbones, to detect structural differences among models. Comparison between **1a** and **1b** PAH cores show equivalent bond lengths (within experimental errors, i.e.  three sigma standard deviations). Hydrogen atoms were included at calculated positions with isotropic U_factors_ = 1.2•U_eq_ or U_factors_ = 1.5•U_eq_ for methyl groups (U_eq_ being the equivalent isotropic thermal factor of the bonded non hydrogen atom). Electron density in **1a** packing voids couldn't be modeled because they are associated with heavily disordered solvent molecules. These contributions correspond to one methanol for each **1a** phenazine diimide molecule and they have been removed with Platon SQUEEZE^[14]^ routine (16 e^-^/cell squeezed in 57 Å^3^ volume voids in **1a** at 100K and 10 e^-^/cell squeezed in 61 Å^3^ volume voids in **1a** at 298K). Pictures were prepared using Ortep-3^[15]^ and CCDC Mercury^[16]^ software. Essential crystal and refinement data are reported below (Table S2 - contribution of disordered solvent removed by the SQUEEZE procedure is included).

**1** and **2** have been crystallized in triclinic centrosymmetric space group *P* -1 with one molecule per cell (Figure S82). Molecules barycenter lay on crystallographic inversion centers. **1a** and **1b** phenazine diimide core are almost flat (distance of core atoms from the mean plane are d < 0.17 Å) as expected on a system with extensive π electrons delocalization. No phase transition has been found for **1a** crystals upon cooling from 298K to 100K (Table S2). Crystal packing in **1a** show extensive hydrophobic interactions with multiple CH•••π contacts (average d_CH•••π_ = 3.7(1) Å and Λ_C-H,π_ = 70°) and poor π•••π stacking (closer ring centroids at d_π•••π_ = 4.0(1) Å, with ~2 Å slippage), while the opposite is found in **1b** (no CH•••π detected; closer ring centroids at d_π•••π_ = 3.361(1) Å, with 0.09 Å slippage). Dichlorobenzene and methanol solvent molecules have been found in crystal packing voids of **1a** and only part of them could be modeled since they adopt multiple conformations (due to the presence of poorly specific CH•••π contacts).

**Table S2**. Crystallographic data and refinement details for **1a** and **1b**.

|  | **1a at 100K** | **1a at 298K** | **1b at 100K** |
| --- | --- | --- | --- |
| CCDC Number | 2280881 | 2280882 | 2280883 |
| Chemical Formula | C_48_H_42_N_4_O_4_  ∙^7^/_2_(C_6_H_4_Cl_2_)∙^3^/_2_(CH_4_O) | C_48_H_42_N_4_O_4_  ∙^7^/_2_(C_6_H_4_Cl_2_)∙^3^/_2_(CH_4_O) | C_32_H_26_N_4_O_4_ |
| Formula weight | 1301.39 g/mol | 1301.39 g/mol | 530.57 g/mol |
| Temperature | 100(2) K | 298(2) K | 100(2) K |
| Wavelength | 0.620 Å | 0.620 Å | 0.620 Å |
| Crystal system | Triclinic | Triclinic | Triclinic |
| Space Group | *P* -1 | *P* -1 | *P* -1 |
| Unit cell dimensions | *a* = 9.334(2) Å | *a* = 9.402(2) Å | *a* = 4.716(1) Å |
|  | *b* = 10.741(2) Å | *b* = 10.902(2) Å | *b* = 10.922(2) Å |
|  | *c* = 17.503(4) Å | *c* = 17.705(4) Å | *c* = 12.609(3) Å |
|  | *α* = 86.46(3)° | *α* = 85.43(3)° | *α* = 107.74.46(3)° |
|  | *β* = 78.17(3)° | *β* = 78.75(3)° | *β* = 93.69(3)° |
|  | *γ* = 73.98(3)° | *γ* = 74.24(3)° | *γ* = 96.23(3)° |
| Volume | 1650.8(6) Å^3^ | 1712.4(7) Å^3^ | 611.6(2) Å^3^ |
| Z | 1 | 1 | 1 |
| Density (calculated) | 1.309 g·cm^-3^ | 1.262 g·cm^-3^ | 1.440 g·cm^-3^ |
| Absorption coefficient | 0.242 mm^-1^ | 0.233 mm^-1^ | 0.073 mm^-1^ |
| F(000) | 676 | 676 | 278 |
| Theta range  for data collection | 1.7° to 30.0° | 2.0° to 29.0° | 1.5° to 31.1° |
| Index ranges | -15 ≤ h ≤ 15,  -16 ≤ k ≤ 16,  -28 ≤ l ≤ 28 | -14 ≤ h ≤ 14,  -16 ≤ k ≤ 16,  -27 ≤ l ≤ 27 | -7 ≤ h ≤ 7,  -18 ≤ k ≤ 18,  -19 ≤ l ≤ 19 |
| Reflections collected | 74505 | 75704 | 28620 |
| Independent reflections  (data with I>2σ(I)) | 13765 (12208) | 12847 (8474) | 5320 (3889) |
| Resolution | 0.62 Å | 0.64 Å | 0.60 Å |
| Data multiplicity  (max resltn) | 5.14 (3.70) | 5.54 (4.52) | 4.85 (2.89) |
| I/σ(I) (max resltn) | 12.27 (7.86) | 5.31 (2.32) | 11.74 (5.10) |
| R_merge_ (max resltn) | 0.0637 (0.0900) | 0.0993 (0.3116) | 0.0663 (0.1460) |
| Data completeness  (max resltn) | 95.0% (86.7%) | 93.9% (88.8%) | 90.1% (75.3%) |
| Refinement method | Full-matrix  least-squares on F^2^ | Full-matrix  least-squares on F^2^ | Full-matrix  least-squares on F^2^ |
| Data / restraints / parameters | 13765 / 39 / 483 | 12847 / 225 / 483 | 5320 / 0 / 182 |
| Goodness-of-fit on F^2^ | 1.037 | 1.013 | 1.041 |
| Δ/σ_max_ | 0.000 | 0.004 | 0.001 |
| Final R indices [I>2σ(I)] | R_1_ = 0.1008  wR_2_ = 0.2906 | R_1_ = 0.0981  wR_2_ = 0.2403 | R_1_ = 0.0582  wR_2_ = 0.1628 |
| R indices (all data) | R_1_ = 0.1068  wR_2_ = 0.2984 | R_1_ = 0.1244  wR_2_ = 0.2626 | R_1_ = 0.0793  wR_2_ = 0.1792 |
| Largest diff. peak and hole | 1.705 and -0.876 eÅ^-3^ | 0.550 and -0.403 eÅ^-3^ | 0.436 and -0.454 eÅ^-3^ |
| R.M.S. deviation  from mean | 0.133 eÅ^-3^ | 0.079 eÅ^-3^ | 0.080 eÅ^-3^ |
|  |  |  |  |

*R*_1_ = Σ ||*F*o|–|*F*c|| / Σ |*F*o|*, wR*_2_ = {Σ [*w*(*F*o^2^ – *F*4 )^2^] / Σ [*w*(*F*o^2^ )^2^]}^½^

**A)
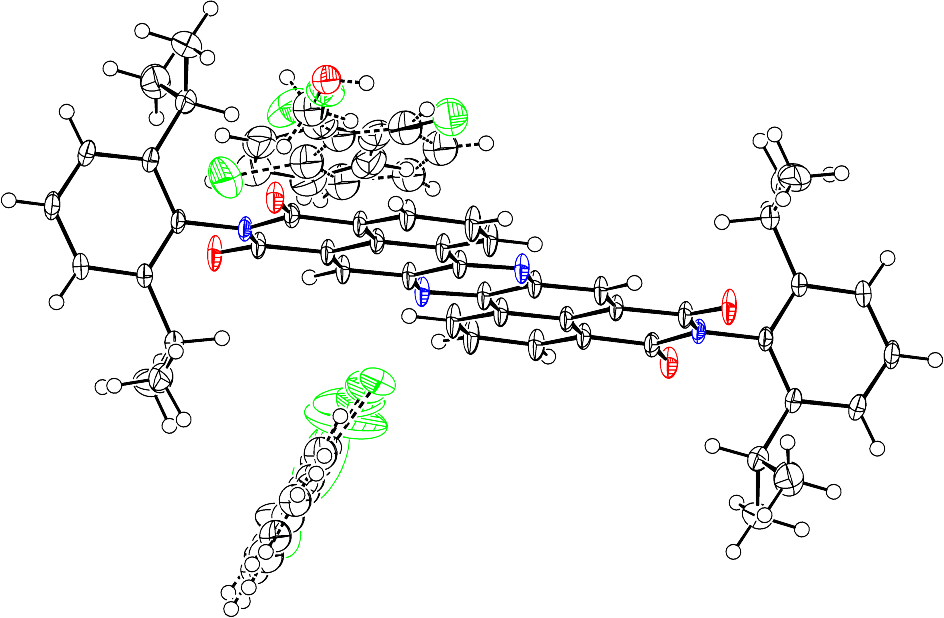
**

**B)
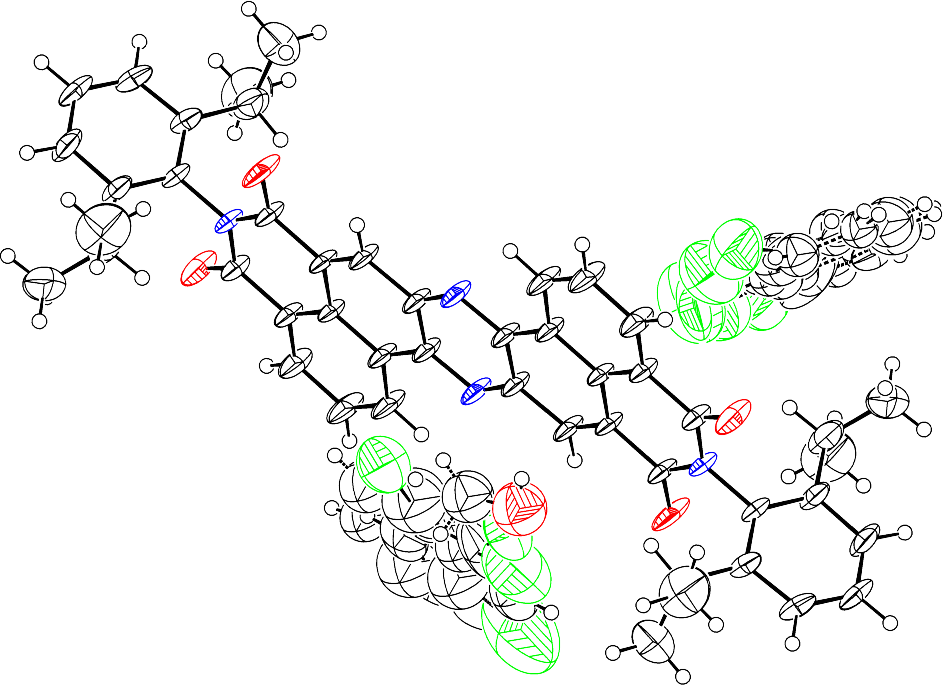
**

**C)
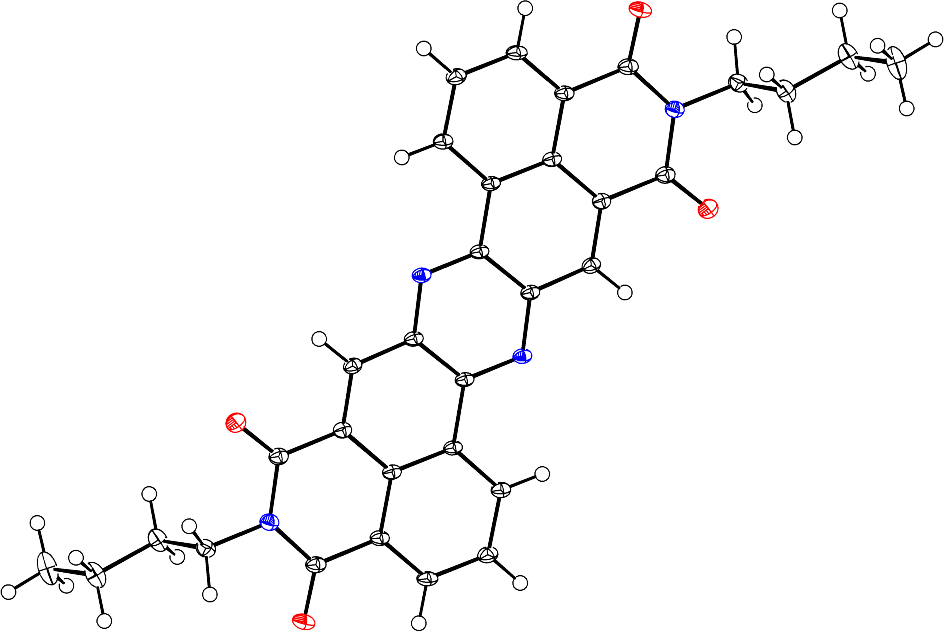
 Figure S66.** Ellipsoids representation of (A) **1a** at 100K, (B) **1a** at 298K and (C) **1b** at 100K molecular structures (50% probability – disordered solvent for **1a** is shown).

# **8 DFT calculations**

All geometry optimizations were performed at M062x functional^[17]^ using def2-TZVP^[18]^ basis set with continuum solvation model. For the continuum solvation model, THF was used as solvent via keywords SCRF=(IEFPCM, solvent=tetrahydrofuran). NBO analysis was also performed using the NBO version 3.1 program^[19]^ to estimate natural charges on the molecules computed. In all the structures optimized, frequency calculations were carried out to ensure that are true relative minima of energy. TD-DFT calculations were performed at the same level of theory to compute the electronic transitions of the optimised structures. All the computations were carried out using the Gaussian-16 suit of programs.^[20]^

**
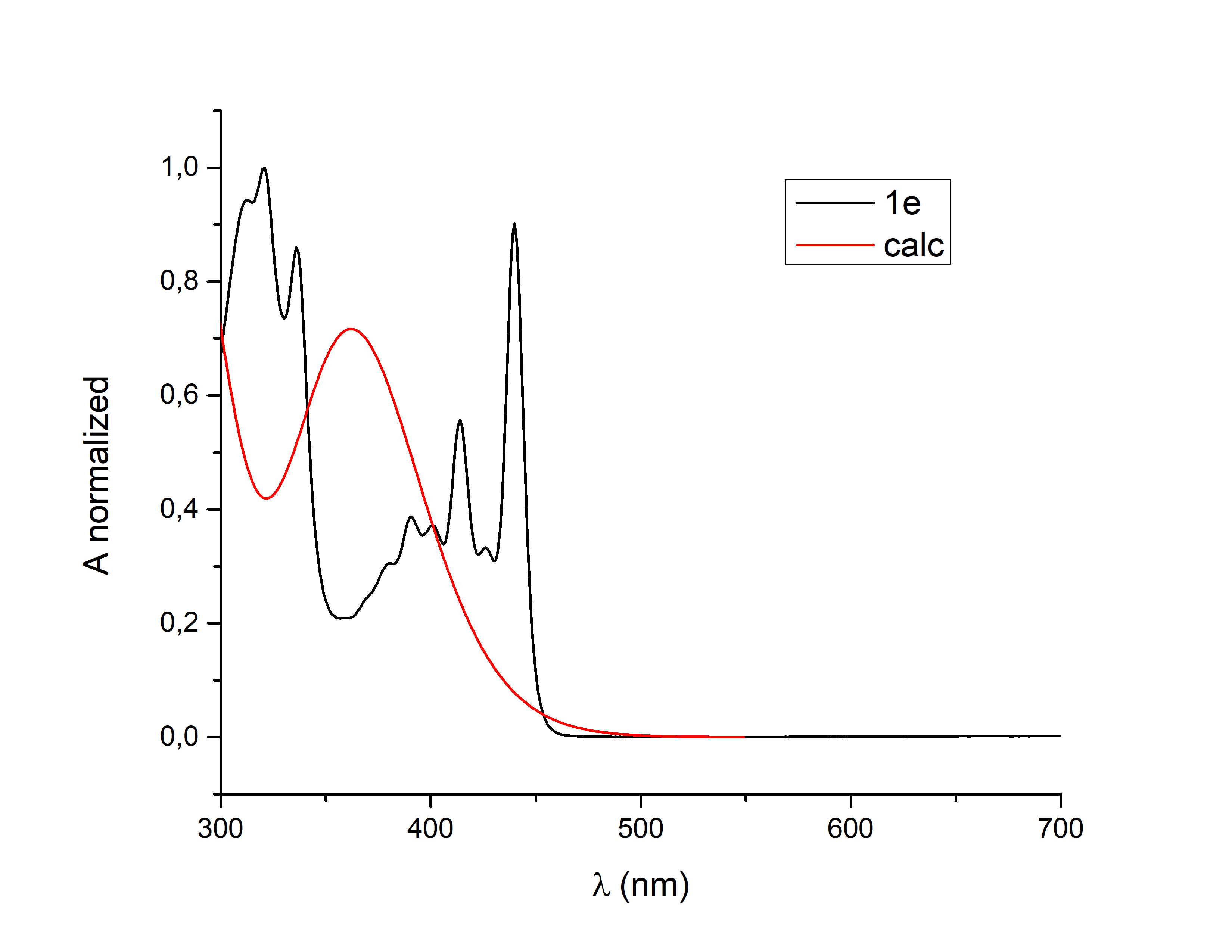
**

**Figure S67.** Simulated (red) and experimental (black) UV-Vis absorption spectra of **1e** in THF.

**
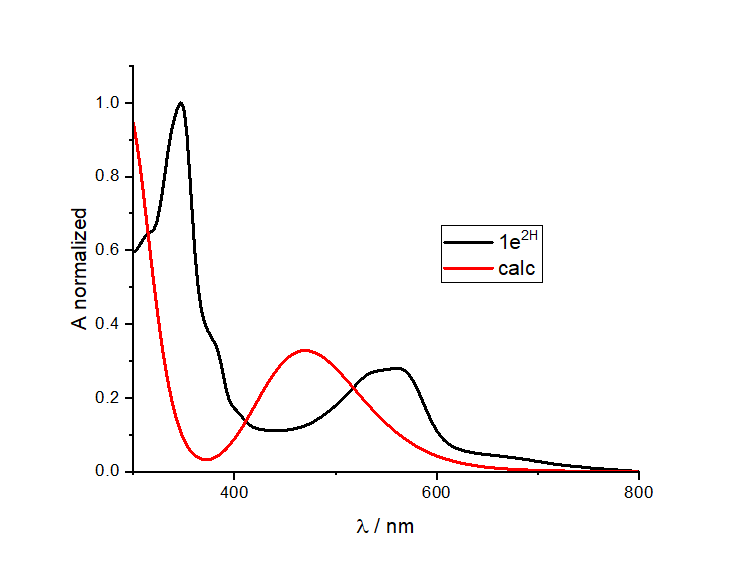
**

**Figure S68.** Simulated (red) and experimental (black) UV-Vis absorption spectra of **1e^2H^** in THF.

**
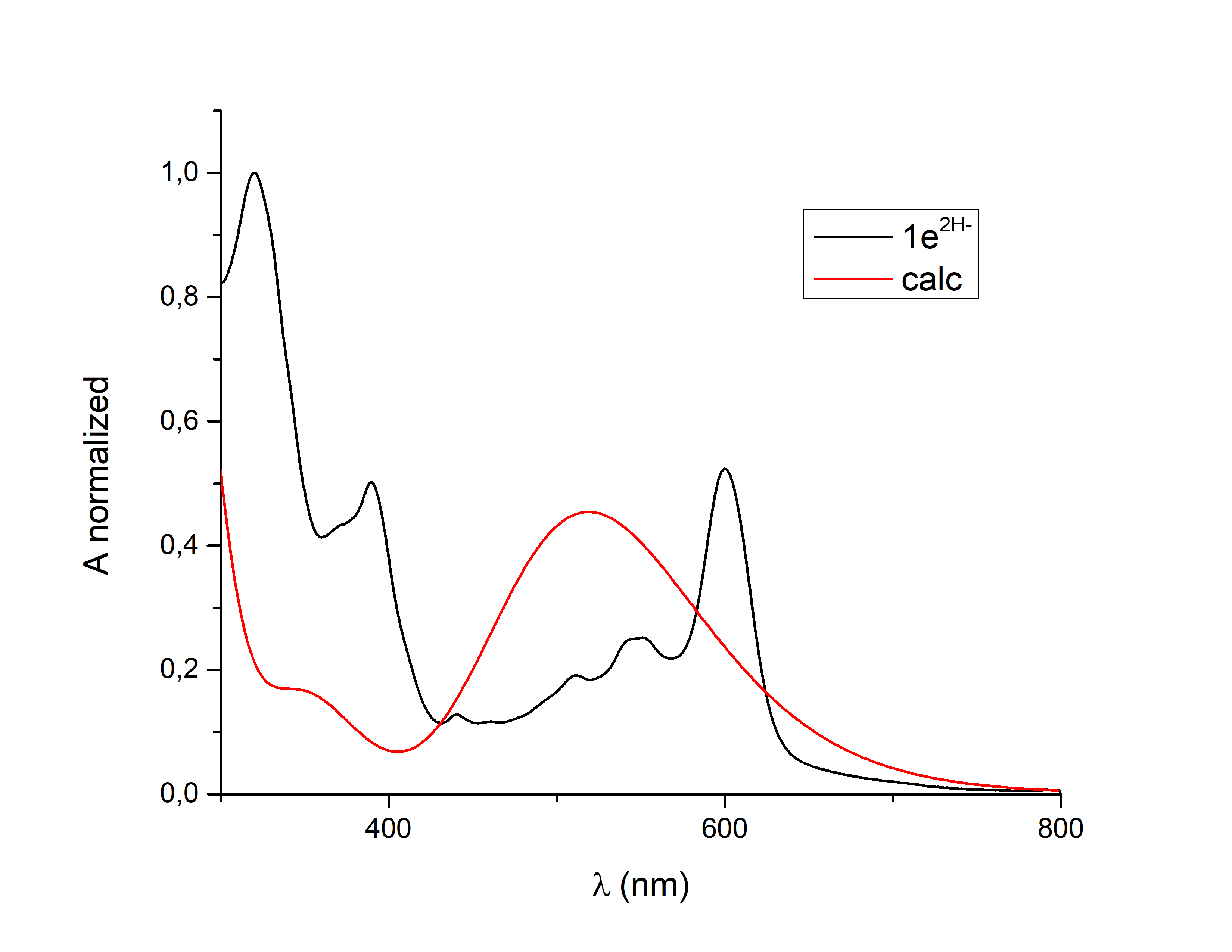
**

**Figure S69.** Simulated (red) and experimental (black) UV-Vis absorption spectra of **1e^2H-^** in THF.

**Cartesian coordinates of the optimized structures**

**1**; 62 atoms; singlet; neutral; Electronic energy: -1193956.39187

C 2.55796600 -1.05791800 0.08601600

C 1.22589200 -0.53213300 0.04361500

C 3.62402100 -0.23180700 -0.00032700

C 1.02268900 0.87064300 -0.09083600

C 2.17215700 1.75151600 -0.18243100

C 3.45816400 1.18829800 -0.13595300

C 2.03408400 3.13881400 -0.31393800

C -1.22589200 0.53213300 -0.04361500

C -1.02268900 -0.87064300 0.09083600

C 4.98830700 -0.82065900 0.04881200

C 5.94725200 1.45707200 -0.17315000

C 4.57989000 2.02354700 -0.22144300

C 3.14818000 3.94745500 -0.39737000

H 3.03283000 5.01774300 -0.49863500

C 4.42618100 3.39000500 -0.35095600

H 5.30755500 4.01336000 -0.41465400

N 6.05643400 0.06842200 -0.04237700

N -0.20152000 1.37791200 -0.13212400

N 0.20152000 -1.37791200 0.13212400

O 6.94135200 2.14181300 -0.24166000

O 5.17992600 -2.00904600 0.16216400

H 1.03960000 3.56172700 -0.34807000

H 2.69013700 -2.12666300 0.18837100

C -2.55796600 1.05791800 -0.08601600

C -2.17215700 -1.75151600 0.18243100

H -2.69013700 2.12666300 -0.18837100

C -3.62402100 0.23180700 0.00032700

C -3.45816400 -1.18829800 0.13595300

C -2.03408400 -3.13881400 0.31393800

C -4.57989000 -2.02354700 0.22144300

H -1.03960000 -3.56172700 0.34807000

C -3.14818000 -3.94745500 0.39737000

C -4.42618100 -3.39000500 0.35095600

H -3.03283000 -5.01774300 0.49863500

H -5.30755500 -4.01336000 0.41465400

C -5.94725200 -1.45707200 0.17315000

C -4.98830700 0.82065900 -0.04881200

N -6.05643400 -0.06842200 0.04237700

O -6.94135200 -2.14181300 0.24166000

O -5.17992600 2.00904600 -0.16216400

C 7.38537000 -0.48851600 0.00472700

C 8.01496000 -0.63851400 1.22889100

C 8.00698100 -0.86369900 -1.17453300

C 9.29429500 -1.17537000 1.27191000

H 7.50442100 -0.33667300 2.13444700

C 9.28634000 -1.39994800 -1.12525100

H 7.49043400 -0.73528600 -2.11708300

C 9.92932900 -1.55560700 0.09628900

H 9.79392800 -1.29619200 2.22386800

H 9.77982400 -1.69581700 -2.04145500

H 10.92658000 -1.97396400 0.13204200

C -7.38537000 0.48851600 -0.00472700

C -8.00698100 0.86369900 1.17453300

C -8.01496000 0.63851400 -1.22889100

C -9.28634000 1.39994800 1.12525100

H -7.49043400 0.73528600 2.11708300

C -9.29429500 1.17537000 -1.27191000

H -7.50442100 0.33667300 -2.13444700

C -9.92932900 1.55560700 -0.09628900

H -9.77982400 1.69581700 2.04145500

H -9.79392800 1.29619200 -2.22386800

H -10.92658000 1.97396400 -0.1320420

**Dihydrophenazine**; 64 atoms; singlet; neutral; Electronic energy: -1194699.43953

C 2.58924200 -1.05007400 0.07894500

C 1.28499000 -0.51054500 0.03844400

C 3.64202400 -0.20389200 -0.00747300

C 1.07220900 0.88986800 -0.10075100

C 2.18427500 1.78555400 -0.19810300

C 3.47211800 1.21007800 -0.14285400

C 2.05545500 3.17544600 -0.33877000

C -1.28499000 0.51054500 -0.03844400

C -1.07220900 -0.88986800 0.10075100

C 5.01741300 -0.80413400 0.04396000

C 5.97306800 1.46070000 -0.16833500

C 4.59753200 2.03236300 -0.22266900

C 3.17883700 3.96793800 -0.41435700

H 3.07286900 5.03791600 -0.52112300

C 4.45202300 3.39905100 -0.35481800

H 5.33806300 4.01655200 -0.41288100

N 6.08327000 0.07160200 -0.04326200

N -0.20118600 1.30125400 -0.12960800

N 0.20118600 -1.30125400 0.12960800

O 6.95112600 2.15611900 -0.23074300

O 5.16309900 -1.99365400 0.15622600

H 1.08766700 3.65537200 -0.39479300

H 2.74688000 -2.11578300 0.17877900

C -2.58924200 1.05007400 -0.07894500

C -2.18427500 -1.78555400 0.19810300

H -2.74688000 2.11578300 -0.17877900

C -3.64202400 0.20389200 0.00747300

C -3.47211800 -1.21007800 0.14285400

C -2.05545500 -3.17544600 0.33877000

C -4.59753200 -2.03236300 0.22266900

H -1.08766700 -3.65537200 0.39479300

C -3.17883700 -3.96793800 0.41435700

C -4.45202300 -3.39905100 0.35481800

H -3.07286900 -5.03791600 0.52112300

H -5.33806300 -4.01655200 0.41288100

C -5.97306800 -1.46070000 0.16833500

C -5.01741300 0.80413400 -0.04396000

N -6.08327000 -0.07160200 0.04326200

O -6.95112600 -2.15611900 0.23074300

O -5.16309900 1.99365400 -0.15622600

C 7.41412500 -0.49171600 0.00499800

C 8.03033400 -0.66007000 1.23264800

C 8.03858300 -0.84931600 -1.17722100

C 9.30712100 -1.20164600 1.27529900

H 7.51543400 -0.36857200 2.13902500

C 9.31561400 -1.38962200 -1.12579100

H 7.53060600 -0.70462400 -2.12186900

C 9.94806300 -1.56541000 0.09805800

H 9.80045800 -1.33766500 2.22818000

H 9.81541700 -1.67221100 -2.04247900

H 10.94393500 -1.98652800 0.13436100

C -7.41412500 0.49171600 -0.00499800

C -8.03858300 0.84931600 1.17722100

C -8.03033400 0.66007000 -1.23264800

C -9.31561400 1.38962200 1.12579100

H -7.53060600 0.70462400 2.12186900

C -9.30712100 1.20164600 -1.27529900

H -7.51543400 0.36857200 -2.13902500

C -9.94806300 1.56541000 -0.09805800

H -9.81541700 1.67221100 2.04247900

H -9.80045800 1.33766500 -2.22818000

H -10.94393500 1.98652800 -0.13436100

H -0.37689000 2.30142400 -0.22860100

H 0.37689000 -2.30142400 0.22860100

**Meisenheimer complex**; 64 atoms; singlet; doubled negative charged; Electronic energy: -1194856.04588

C 2.56912600 -1.22716900 0.04153800

C 1.21862300 -0.56686500 0.02212200

C 3.71855200 -0.26354000 -0.03519900

C 1.06191400 0.82288200 -0.10328300

C 2.23120900 1.70701800 -0.20841200

C 3.53732100 1.11906500 -0.16146000

C 2.08680500 3.07151000 -0.33883300

C -1.21862300 0.56686500 -0.02212200

C -1.06191400 -0.82288200 0.10328300

C 5.00947600 -0.81250100 0.01526300

C 6.00035100 1.47553000 -0.19708000

C 4.64583400 2.00202700 -0.24528100

C 3.19637100 3.92479100 -0.42380700

H 3.05524100 4.99243200 -0.52403300

C 4.46130500 3.38234500 -0.37327700

H 5.34279800 4.00828300 -0.43014400

N 6.10226700 0.10161700 -0.06738100

N -0.16086700 1.35775100 -0.12240100

N 0.16086700 -1.35775100 0.12240100

O 7.01518800 2.17303200 -0.26281800

O 5.27458900 -2.02266000 0.12470400

H 1.08465200 3.47924000 -0.37088900

H 2.63611600 -1.84445200 0.94450700

C -2.56912600 1.22716900 -0.04153800

C -2.23120900 -1.70701800 0.20841200

H -2.63611600 1.84445200 -0.94450700

C -3.71855200 0.26354000 0.03519900

C -3.53732100 -1.11906500 0.16146000

C -2.08680500 -3.07151000 0.33883300

C -4.64583400 -2.00202700 0.24528100

H -1.08465200 -3.47924000 0.37088900

C -3.19637100 -3.92479100 0.42380700

C -4.46130500 -3.38234500 0.37327700

H -3.05524100 -4.99243200 0.52403300

H -5.34279800 -4.00828300 0.43014400

C -6.00035100 -1.47553000 0.19708000

C -5.00947600 0.81250100 -0.01526300

N -6.10226700 -0.10161700 0.06738100

O -7.01518800 -2.17303200 0.26281800

O -5.27458900 2.02266000 -0.12470400

C 7.42232900 -0.45422500 -0.00409800

C 8.03361000 -0.63266200 1.22684700

C 8.08102300 -0.80787900 -1.17139800

C 9.31214500 -1.16939600 1.29102200

H 7.50107200 -0.35183300 2.12664600

C 9.35952300 -1.34441800 -1.10639600

H 7.58556500 -0.66109900 -2.12268900

C 9.97694500 -1.52636600 0.12463100

H 9.78843300 -1.31049900 2.25273200

H 9.87308800 -1.62171800 -2.01800800

H 10.97340600 -1.94607200 0.17488100

C -7.42232900 0.45422500 0.00409800

C -8.08102300 0.80787900 1.17139800

C -8.03361000 0.63266200 -1.22684700

C -9.35952300 1.34441800 1.10639600

H -7.58556500 0.66109900 2.12268900

C -9.31214500 1.16939600 -1.29102200

H -7.50107200 0.35183300 -2.12664600

C -9.97694500 1.52636600 -0.12463100

H -9.87308800 1.62171800 2.01800800

H -9.78843300 1.31049900 -2.25273200

H -10.97340600 1.94607200 -0.17488100

H -2.60641600 1.94704800 0.78505100

H 2.60641600 -1.94704800 -0.78505100

| **1** | |
| --- | --- |
| **HOMO**  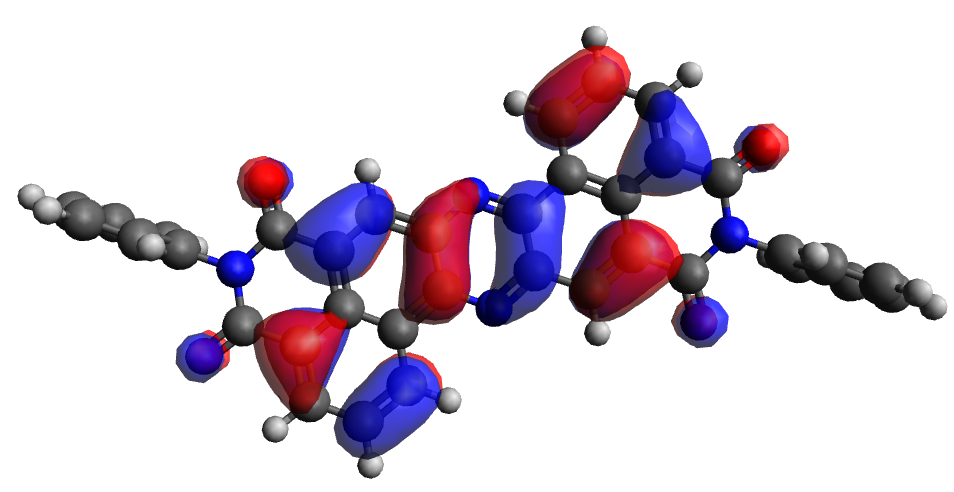  -8.02 eV | **LUMO**  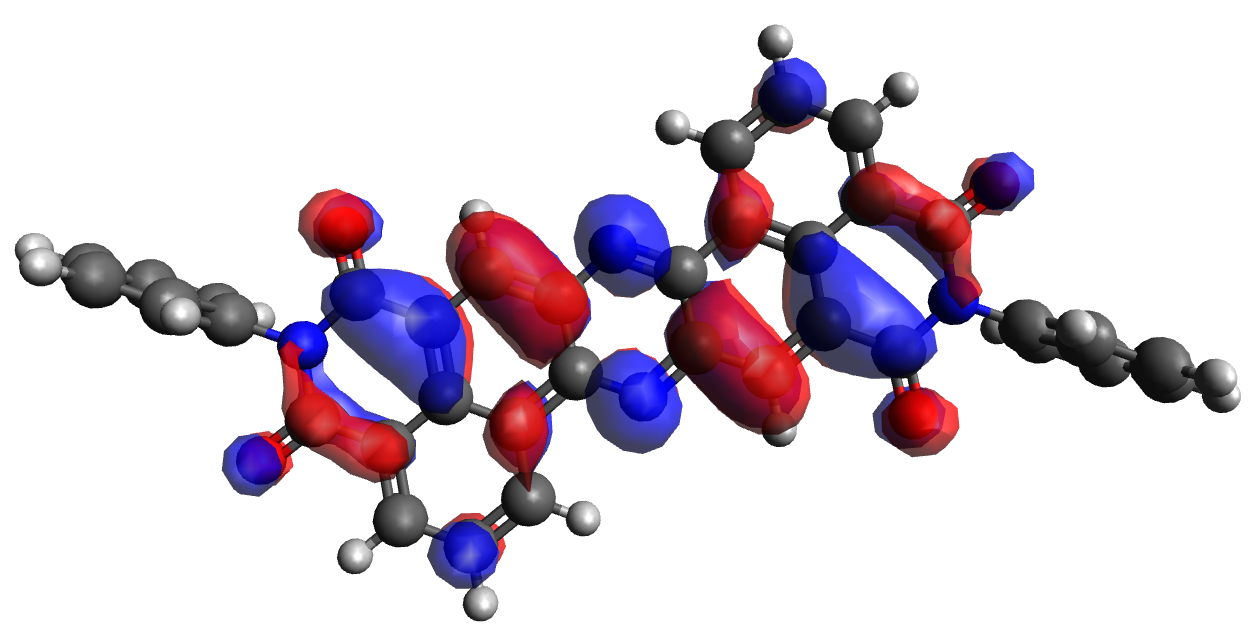  -2.76 eV |
| HOMO-LUMO gap = 5.33 eV | |

**Figure S70.** Selected frontier molecular orbitals and relative energies**.**

# **9 References**

[1] R. Kumar, M. Taddei, V. Petropoulos, M. Russo, F. Vernuccio, G. Cerullo, D. Polli, A. Nenov, N. Demitri, M. Prato, M. Maiuri, J. Dosso, *J. Mater. Chem. C* **2025**, *13*, 2681–2688.

[2] N. F. Fedko, V. F. Anikin, *J. Org. Pharm. Chem.* **2015**, *13*, 12–15.

[3] Y. Tian, H. Yang, X. Li, Y. Wang, Y. Teng, D. Yin, *Org. Lett.* **2021**, *23*, 3782–3787.

[4] X. Zhang, Y. Song, M. Liu, H. Li, H. Sun, M. Sun, H. Yu, *Dye. Pigment.* **2019**, *160*, 799–805.

[5] C. Zhang, K. Ji, X. Wang, H. Wu, C. Liu, *Chem. Commun.* **2015**, *51*, 8173–8176.

[6] L. D. Wescott, D. L. Mattern, *J. Org. Chem.* **2003**, *68*, 10058–10066.

[7] A. Lausi, M. Polentarutti, S. Onesti, J. R. Plaisier, E. Busetto, G. Bais, L. Barba, A. Cassetta, G. Campi, D. Lamba, A. Pifferi, S. C. Mande, D. D. Sarma, S. M. Sharma, G. Paolucci, *Eur. Phys. J. Plus* **2015**, *130*, 1–8.

[8] W. Kabsch, *Acta Crystallogr. Sect. D Biol. Crystallogr.* **2010**, *66*, 125–132.

[9] J. Agirre, M. Atanasova, H. Bagdonas, C. B. Ballard, A. Baslé, J. Beilsten-Edmands, R. J. Borges, D. G. Brown, J. J. Burgos-Mármol, J. M. Berrisford, P. S. Bond, I. Caballero, L. Catapano, G. Chojnowski, A. G. Cook, K. D. Cowtan, T. I. Croll, J. Debreczeni, N. E. Devenish, K. Yamashita, et al., *Acta Crystallogr. Sect. D Struct. Biol.* **2023**, *79*, 449–461.

[10] P. R. Evans, G. N. Murshudov, *Acta Crystallogr. Sect. D Biol. Crystallogr.* **2013**, *69*, 1204–1214.

[11] G. M. Sheldrick, *Acta Crystallogr. Sect. A Found. Crystallogr.* **2015**, *71*, 3–8.

[12] G. M. Sheldrick, *Acta Crystallogr. Sect. C Struct. Chem.* **2015**, *71*, 3–8.

[13] P. Emsley, B. Lohkamp, W. G. Scott, K. Cowtan, *Acta Crystallogr. Sect. D Biol. Crystallogr.* **2010**, *66*, 486–501.

[14] A. L. Spek, *Acta Crystallogr. Sect. C Struct. Chem.* **2015**, *71*, 9–18.

[15] L. J. Farrugia, *J. Appl. Crystallogr.* **2012**, *45*, 849–854.

[16] C. F. MacRae, I. Sovago, S. J. Cottrell, P. T. A. Galek, P. McCabe, E. Pidcock, M. Platings, G. P. Shields, J. S. Stevens, M. Towler, P. A. Wood, *J. Appl. Crystallogr.* **2020**, *53*, 226–235.

[17] Y. Zhao, D. G. Truhlar, *Theor. Chem. Account.* **2008**, *120*, 215–241.

[18] F. Weigend, *Phys. Chem. Chem. Phys*. **2006**, *8*, 1057–1065

[19] E. D. Glendening, A. E. Reed, J. E. Carpenter, and F. Weinhold, NBO Version 3.1.

[20] Gaussian 16, Revision C.01, M. J. Frisch, G. W. Trucks, H. B. Schlegel, G. E. Scuseria, M. A. Robb, J. R. Cheeseman, G. Scalmani, V. Barone, G. A. Petersson, H. Nakatsuji, X. Li, M. Caricato, A. V. Marenich, J. Bloino, B. G. Janesko, R. Gomperts, B. Mennucci, H. P. Hratchian, J. V. Ortiz, A. F. Izmaylov, J. L. Sonnenberg, D. Williams-Young, F. Ding, F. Lipparini, F. Egidi, J. Goings, B. Peng, A. Petrone, T. Henderson, D. Ranasinghe, V. G. Zakrzewski, J. Gao, N. Rega, G. Zheng, W. Liang, M. Hada, M. Ehara, K. Toyota, R. Fukuda, J. Hasegawa, M. Ishida, T. Nakajima, Y. Honda, O. Kitao, H. Nakai, T. Vreven, K. Throssell, J. A. Montgomery, Jr., J. E. Peralta, F. Ogliaro, M. J. Bearpark, J. J. Heyd, E. N. Brothers, K. N. Kudin, V. N. Staroverov, T. A. Keith, R. Kobayashi, J. Normand, K. Raghavachari, A. P. Rendell, J. C. Burant, S. S. Iyengar, J. Tomasi, M. Cossi, J. M. Millam, M. Klene, C. Adamo, R. Cammi, J. W. Ochterski, R. L. Martin, K. Morokuma, O. Farkas, J. B. Foresman, and D. J. Fox, Gaussian, Inc., Wallingford CT, 2016.
